# Supplementary material for: Five centuries of consanguinity, isolation, health, and conflict in Las Gobas: A Northern Medieval Iberian necropolis
Source: Sci Adv. 2024 Aug 28;10(35):eadp8625. doi: 10.1126/sciadv.adp8625 (PMC11352919; doi:10.1126/sciadv.adp8625)
Supplement: 20240828-1 [file sciadv.adp8625.v1.pdf]

GENETICS

Five centuries of consanguinity, isolation, health, and conflict in Las Gobas: A Northern Medieval Iberian necropolis

Ricardo Rodríguez-Varela[1,2]*, Reyhan Yaka[1,2]†, Zoé Pochon[1,2]†, Iban Sanchez-Pinto[3,4], José Luis Solaun[3,4], Thijessen Naidoo[1,2,5], Benjamin Guinet[1,6], Patxi Pérez-Ramallo[7,8], Vendela Kempe Lagerholm[1,2], Violeta de Anca Prado[1], Cristina Valdiosera[9], Maja Krzewińska[1,2], Lourdes Herrasti[10], Agustín Azkarate[3,4], Anders Götherström[1,2]*

Copyright © 2024 The Authors, some rights reserved; exclusive licensee American Association for the Advancement of Science. No claim to original U.S. Government Works. Distributed under a Creative Commons Attribution License 4.0 (CC BY).

Between the 8th and 11th centuries CE, the Iberian Peninsula underwent profound upheaval due to the Umayyad invasion against the Visigoths, resulting in population shifts and lasting demographic impacts. Our understanding of this period is hindered by limited written sources and few archaeogenetic studies. We analyzed 33 individuals from Las Gobas, a necropolis in northern Spain, spanning the 7th to 11th centuries. By combining archaeological and osteological data with kinship, metagenomics, and ancestry analyses, we investigate conflicts, health, and demography of these individuals. We reveal intricate family relationships and genetic continuity within a consanguineous population while also identifying several zoonoses indicative of close interactions with animals. Notably, one individual was infected with a variola virus phylogenetically clustering with the northern European variola complex between ~885 and 1000 CE. Last, we did not detect a significant increase of North African or Middle East ancestries over time since the Islamic conquest of Iberia, possibly because this community remained relatively isolated.

INTRODUCTION

During the medieval period, the Iberian Peninsula became a melting pot of ideas and lifestyles. The region witnessed the convergence of various cultures, religions, and ethnicities creating a rich and diverse social tapestry. The genetic impact of the interaction between these diverse groups is underexplored. Recent data provided a first glance of the influence of Islamic conquest on the Iberian gene pool (*1*). However, the timing and scale of the processes influencing the genetic makeup of the Iberian population over time and across different regions remain uncertain, with a notable dearth of archaeogenetic studies focusing on the Middle Ages in the Iberian Peninsula.

Migrations between North Africa and the Iberian Peninsula have been documented since at least the Neolithic (*2*–*4*). The Phoenician, Greek, and Carthaginian colonies, followed by the Roman Empire, played crucial roles in amplifying the genetic exchange from North Africa to the Iberian Peninsula, especially along its southern and eastern shores (*1*, *5*). The arrival of Berber populations from North Africa during the Umayyad Caliphate's conquest of the Iberian Peninsula (711 to 726 CE) exerted a pronounced genetic influence on most parts of Iberia (*1*, *6*). Between 711 and 1492 CE, diverse Islamic states characterized by distinct political and ethnic origins ruled different extensions of Iberia marked by a continual shifting of borders with the emerging Christian kingdoms in the north (*7*–*9*). Despite the geopolitical and sociocultural boundaries remaining strong between Christians and Muslims (Fig. 1) (*10*), the impact of the North African gene flow during this period remains traceable today (*11*). Bycroft *et al.* (*11*) revealed that the North African ancestry is present in all current Iberian populations, with lower levels in the Basque region and areas corresponding to the 14th century Crown of Aragon. However, this ancestry does not correlate with proximity to North Africa or extended Islamic control. The highest levels, up to 11%, are found in Galicia and Portugal (*11*).

Situated in northern Spain, the rural necropolis of Las Gobas (Fig. 1) spans a time transect from the 7th to the 11th centuries, encompassing individuals who lived both before and during the Islamic control of most of Iberia. This offers the opportunity to study how the dynamic regional development was mirrored in a small

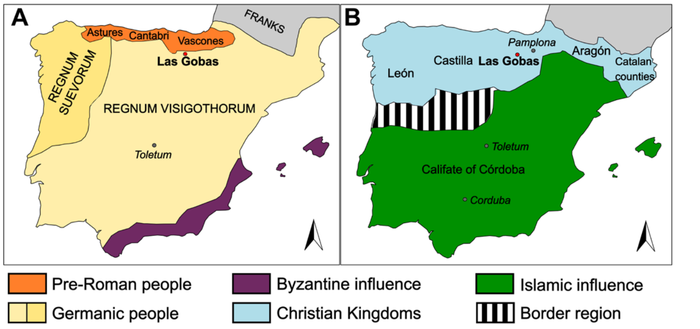

Fig. 1. Map of the Iberian Peninsula. In red, the location of the necropolis of Las Gobas. City names are shown in italics. (**A**) The Iberian Peninsula around the year 569 CE. (**B**) The different kingdoms and border with the Caliphate of Córdoba in the 10th century [maps based on (*103*)].

[1]Centre for Palaeogenetics, Stockholm, Sweden. [2]Department of Archaeology and Classical Studies, Stockholm University, Stockholm, Sweden. [3]Departamento de Geografía, Prehistoria y Arqueología, University of the Basque Country, UPV/EHU, Vitoria-Gasteiz, Spain. [4]GPAC, C. I. Micaela Portilla, University of the Basque Country, UPV/EHU, Vitoria-Gasteiz, Spain. [5]Ancient DNA Unit, Science for Life Laboratory, Stockholm, Sweden. [6]Department of Bioinformatics and Genetics, Swedish Museum of Natural History, Stockholm, Sweden. [7]Department of Archaeology and Cultural History, NTNU University Museum, Trondheim, Norway. [8]isoTROPIC Research Group, Department of Archaeology, Max Planck Institute of Geoanthropology, Jena, Germany. [9]Universidad de Burgos, Departamento de Historia, Geografía y Comunicaciones, Burgos, Spain. [10]Departamento de Antropología, Sociedad de Ciencias Aranzadi, Donostia-San Sebastián, Spain.
*Corresponding author. Email: ricardo.rodriguez.varela@arklab.su.se (R.R.-V.); anders.gotherstrom@arklab.su.se (A.G.)
†These authors contributed equally to this work.

rural community throughout a key period in shaping the genetic structure of the Iberian Peninsula. Specifically, it allows for an in-depth exploration of the genetic impact of sociocultural conflicts and population interactions within this rural medieval Christian community. Furthermore, it enables us to investigate the dissemination of pathogenic microbes during these interactions and glean insights into the potential diseases prevalent among these populations, shedding light on their way of life. Archaeogenetics, by filling critical gaps in information for this period and region, becomes a powerful tool in unraveling aspects of ancestry, health, and social interactions influenced by historical events.

Las Gobas is part of the extensive complex of medieval cave settlements in Iberia, a topic of long-standing debate concerning their temporal origins and intended purposes in European historiography. The designation "Las Gobas" refers to a cluster of 13 artificial caves meticulously carved into a rocky outcrop, spanning from the mid-6th century to the 11th century CE (*12*, *13*). This cluster includes two churches and variously sized single-room cavities.

Archaeological investigations (*14*) reveal that the necropolis consists of two distinct periods. Phase I (seventh–ninth centuries CE) is characterized by mixed habitation of cave architecture and free-standing structures, featuring a church (Las Gobas-6) and a cemetery containing 22 burials (Fig. 2). This phase, marked by the construction of new domestic structures outside the church, shows an active habitat with clear evidence of daily life and activities.

In phase II (10th–11th centuries CE), the settlement shed its residential character, preserving only its role as a cemetery and place of worship. A total of 19 burials have been unearthed from this latter period (Fig. 2 and table S1). Phase II begins with the abandonment of the site as a living area, as indicated by sedimentation layers and the remains of an adult cow found in a silo repurposed as a waste dump. Radiocarbon dating places this transition in the second half of the ninth century CE. The shift from a residential area to a funerary space and the eventual relocation of inhabitants to a new settlement in Laño clearly demarcate the two phases in the history of this cemetery at Las Gobas (*14*).

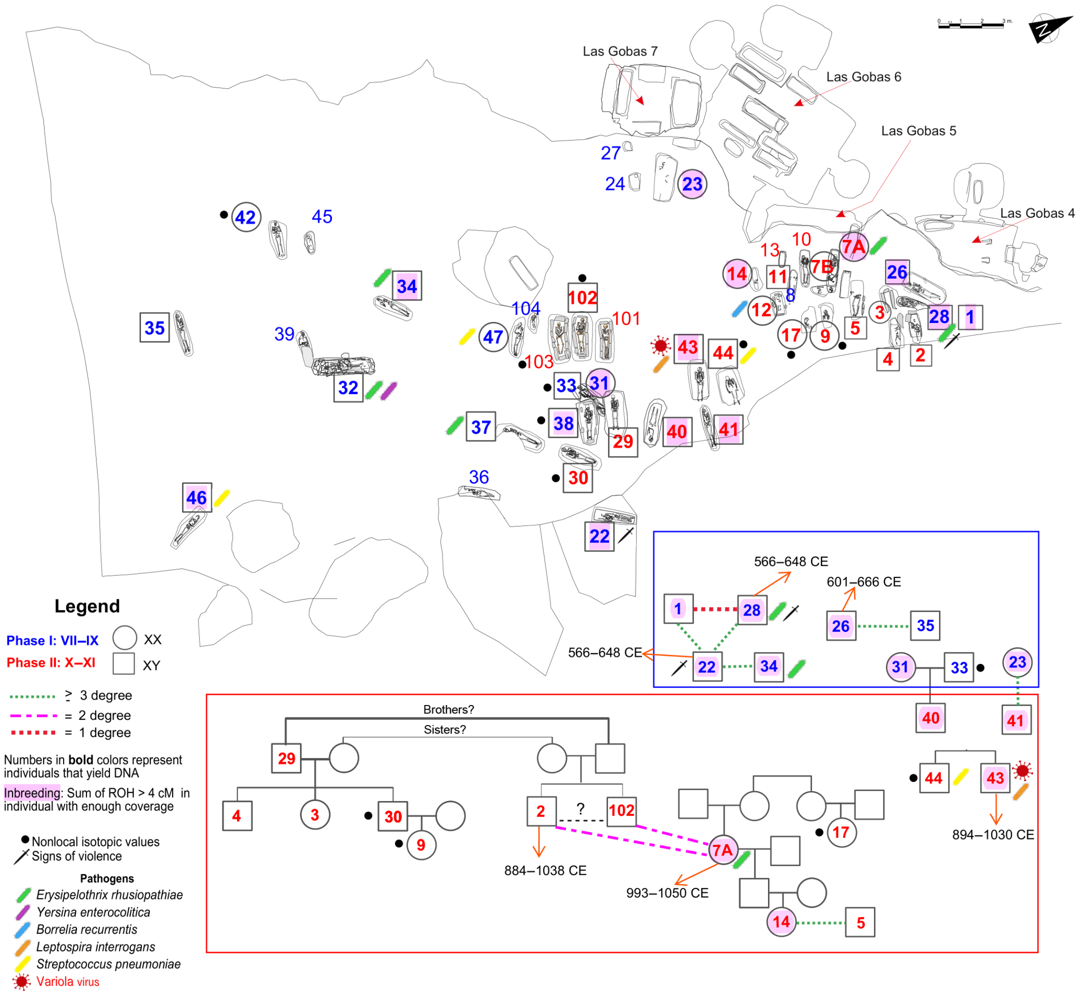

Fig. 2. Kinship and burial distributions at Las Gobas. We represent here one of the possible pedigrees of the related individuals according to their autosomal, uniparental, archaeological, and anthropological data (see tables S1 to S4). The inbreeding information is listed in table S5, while the isotopic and osteological information was taken from (*15*, *16*), respectively. Each bacteria species is uniquely represented by a distinct color. In addition, the variola virus is symbolized with a virus symbol. In the individual with a direct radiocarbon date, we display the 2-sigma date interval.

Earlier bioarchaeological investigations have underscored the distinctive nature of this archaeological site. Noteworthy among these are isotopic studies suggesting a shift in dietary practices over time and moderate mobility patterns (*15*). Particularly noteworthy is a recent work (*16*), which focuses on examining the skeletal remains of adult males showcasing severe cranial injuries inflicted by swords, indicative of past violent episodes.

Here, we analyze the genomes of 33 individuals buried in Las Gobas, which encompass five centuries of political, demographic, and social changes in the Iberian Peninsula. Using an archaeogenetic strategy, we delve into the genetic origin, consanguinity, and kin relations of these individuals. Furthermore, we pioneer the exploration of the infectious diseases landscape within an early Medieval Iberian community using a metagenomic approach. Last, we conducted absolute radiocarbon dating that allows us to shed light on two individuals from the first phase of the necropolis showing clear evidence of trauma generated by a violent episode. Our results are integrated in a holistic perspective with previous components such as osteological and isotopic analyses and thus combinedly will help to understand the origin and functionality of the site.

RESULTS

Genomic analyses

We generated whole-genome sequence data for 48 skeletal elements belonging to 37 individuals. Four individuals with a coverage <0.01× were removed, and the remaining 33 were sequenced to a coverage of 0.015× to 5.37× (median = 1, mean = 1.27) (table S2). All sequenced genomic libraries presented short fragment length, clear damage patterns, and negligible levels of contamination, indicating that the data generated here are endogenous and authentic (table S2).

Sex identification and uniparental markers

We genetically identified the biological sex of each of the 33 individuals following the method described in (*17*), with clear assignments obtained for all individuals as 11 females (XX) and 22 males (XY) (table S2). Of the 22 males, all but three showed the Y chromosome haplogroup R1b1a1b (R-M269) or one of its related subbranches (table S3), a genetic lineage that has been prevalent in Europe since the Bronze Age (*18*, *19*). The specific haplogroup of two of the three individuals could not be determined due to limited data, while individual 37 was found to have the haplotype L1b1, which is relatively uncommon in Europe and can be found in Middle Eastern populations today (*20*). On the other hand, the mitochondrial haplogroups exhibited higher diversity, representing all major European lineages including H, J, I, U, K, HV, and T (table S2).

Violence, kinship, and extended family structures

From phase I of the necropolis (direct radiocarbon dated between 610 and 907 cal CE), 21 burials have been recovered, of which 14 are males, 5 are females, and 2 are infants. We retrieved genomic data from 15 of these individuals (11 males and 4 females) and included them in the kinship analyses. Our results indicated that only six of them were not related (≥4 degree) to any other individual analyzed here. Among the related individuals, we found a first-degree relation between two males, individual 1 and individual 28, with the kinship results suggesting that they were full siblings (table S4). These potential brothers are at least third-degree related to individual 22.

Individuals 22 and 28 present several traumas caused by sword on their skulls (see fig. S1, A and B). Osteological analysis suggests that at least one of them (individual 28) died from these injuries (fig. S1B) (*16*) (see notes 3 and 5 in table S1). All this points to violent episodes during an early phase of occupation of Las Gobas as both individuals have the oldest direct radiocarbon dates (566 to 648 cal CE; table S1).

Individual 22 was also a third-degree relative of individual 34. Another pair of males, individuals 26 and 35, were also third-degree related to each other, probably cousins on the paternal side (Fig. 2 and table S4). All of them were associated with the oldest layer in the necropolis. Note that some burials functioned as hinges between phase I and phase II, suggesting continuity between both phases. This is well represented by the only family trio in which individuals from phase I (33 and 31) had a son (individual 40) associated with phase II. In addition, individual 23 (from phase I) is a third-degree relative of individual 41, who is also part of phase II (Fig. 2 and table S4).

We documented an increase of genetically related individuals during phase II (direct radiocarbon date from 979 to 1036 cal CE) when the Las Gobas community relocated to the valley floor as a farming community. From this phase, there were a total of 19 burials: 11 males, 3 females, and 5 infants. We retrieved genomic data from 18 of these individuals (11 males and 7 females) with at least 15 of them related to other individuals from Las Gobas. This included a three-generation family with genomic data from seven members, two of them (individual 2 and 102) are also second or third degree related to individual 7A from another family group (Fig. 2 and table S4). Two brothers having children with two sisters would be one of the possible pedigrees for this family that agrees with our uniparental and kinship results and also with the available archaeological, radiocarbon, and osteological data (Fig. 2 and table S1). We noted that due to the low coverage of individuals 2 and 102, the kinship relation between them could not be determined (Fig. 2). Although they overlap in more than 10,000 Single Nucleotide Polymorphisms (SNPs) with the other individuals, the relations involving these individuals should be taken with caution. However, they could probably be brothers as they seem equally related to the rest of the individuals (table S4).

Consanguinity

The frequency and length of runs of homozygosity (ROH) rise in correlation with the level of genealogical proximity between the two parents allowing for investigating the degree of inbreeding (*21*). We use the software hapROH (*22*), which identifies ROH in individuals with coverage ≥0.3× using reference haplotypes, to calculate and visualize the degree of consanguinity in all the Las Gobas and reference ancient Iberian individuals with >400,000 SNPs of the 1240K (*23*) sites covered (table S5). Several individuals presented high levels of consanguinity, especially in the first phase of Las Gobas. ROH between 4 and 8 centimorgan (cM) suggests ancestral relatedness in both phases (Fig. 3A and table S5). We also found three individuals (31, 34, and 38) from phase I and individual 41 from phase II with long ROH fragments (Fig. 3, A and B). The sum of ROH fragments > 20 cM suggest that the parents of these individuals were closely related (Fig. 3B). Our results indicate that within the reference ancient Iberian individuals (table S5), levels of inbreeding (sum of ROH fragments > 20 cM) were also present in one Iberian Islamic individual (I7457) (table S5).

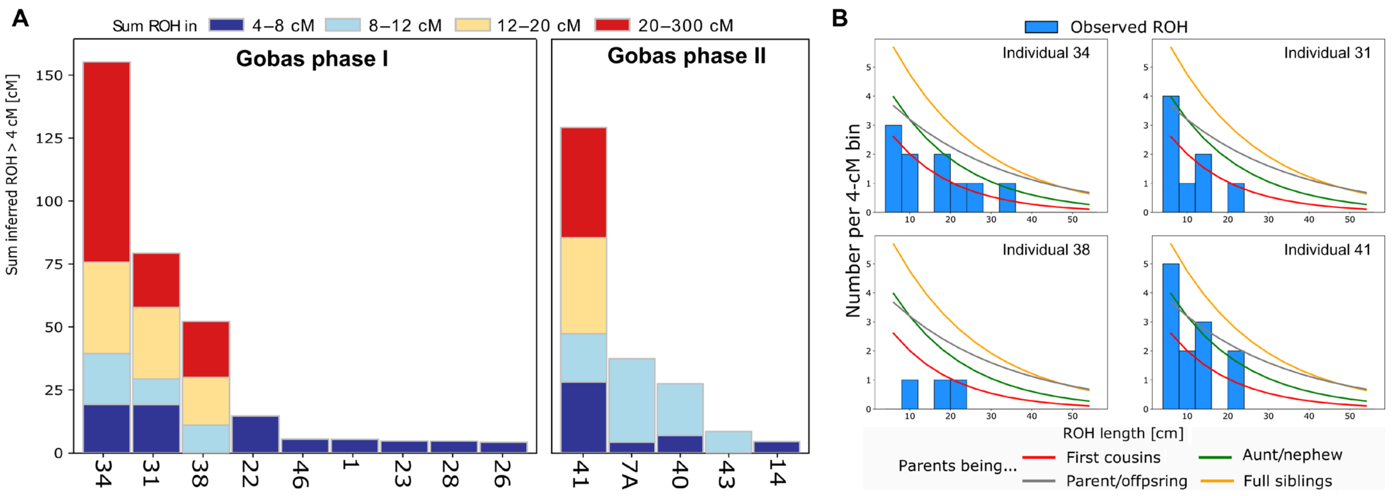

Fig. 3. Runs of homozygosity (ROH). (**A**) ROH analysis for individuals from Las Gobas, exhibiting cumulative ROH lengths exceeding 4 cM and coverage of more than 400,000 SNPs from the 1240K dataset (*23*). The numbers on the *x* axis represent individual codes. (**B**) ROH lengths and expected densities of ROH for certain degrees of parental relationships (*22*) for the Las Gobas individual with the highest sum of inferred ROH.

Metagenomic analyses

In total, we identify six pathogens in the population of Las Gobas (table S6). Four pathogenic species were detected based on aMeta's authentication scores (*24*): *Erysipelothrix rhusiopathiae*, *Leptospira interrogans*, *Streptococcus pneumoniae*, and *Yersinia enterocolitica*. Since viruses might be harder to detect due to low coverage, and because database configuration can affect the number of taxReads assigned to the species level, we investigated the *k*-mers/reads ratio of pathogenic organisms detected with KrakenUniq (*25*) to assess their presence (see Materials and Methods). We further identified two additional pathogens namely *Borrelia recurrentis* (ratio = 16.6) and variola virus (ratio = 7.9) (table S6) under the aMeta's default thresholds (200 taxReads and 1000 *k*-mers). Because of database configuration in National Center for Biotechnology Information (NCBI), *B. recurrentis* only exhibited 2 reads at the species level (taxReads), but actually 359 when encompassing the lower taxonomic nodes and 5958 *k*-mers. The variola virus was just under the thresholds with 195 taxReads, 202 reads, and 1597 *k*-mers. Given its historical interest, individual 43 infected with variola virus was subjected to another round of shotgun sequencing.

To identify potential host-associated microbes and pathogens mentioned above using aMeta, we used an elimination process. Authentication scores to evaluate true presence and ancient status were generated by the aMeta pipeline (*24*) based on nine metrics (edit distance, edit distance of ancient reads, deamination on both read ends, mean read length, postmortem deamination score, average nucleotide identity, amount of reads, and evenness of coverage, the latter adding two points). Through this method, we detected 56 microbial organisms with an authentication score of ≥8 (see Materials and Methods and figs. S2 and S3). However, 41 were filtered out (fig. S3) as they could not be disentangled from environmental contamination. Among the remaining 15 (fig. S2), 12 were associated with the human oral microbiome (*26*–*28*) (among which one is a potentially asymptomatic pathogen), and the remaining 3 were pathogenic.

S. pneumoniae, the potentially asymptomatic pathogen, was authenticated in individuals 44, 46, and 47 (figs. S4 to S6) but could not be validated in individual 43 based on authentication plots. This bacterium, present in the oral microbiome of about 10% of adults and more children, can cause respiratory infections, pneumonia, and bacteremia (*29*).

The three remaining identified pathogenic species by aMeta are *E. rhusiopathiae*, confirmed in individuals 28, 32, 34, and 37 from phase I and in individual 7A from phase II (range of breadth of coverage 2.26 to 92.44%), *L. interrogans* in individual 43, and *Y. enterocolitica* in individual 32 (tables S6 to S8 and figs. S2 and S7 to S14). These pathogens are responsible for distinct diseases: erisypeloid, a skin infection transmitted through wound contact with infected animal material; leptospirosis, a potentially fatal disease leading to organ failure, typically contracted from contact with contaminated water; and yersiniosis, a severe form of gastroenteritis, respectively. The consistent coverage of genes associated with virulence, such as *inv*, *yst*, and *myf*, supports the pathogenic nature of the identified *Y. enterocolitica* (figs. S15 to S17). Competitive mapping further validates our results for these three pathogens (table S7).

In the case of *E. rhusiopathiae*, two of the five candidates had confident high coverage (47.7 and 92.4%), while the remaining three had low coverage (less than 10%). The latter three samples were included in the phylogenetic analysis to test their phylogenetic signal leading to a clear *E. rhusiopathiae* origin (see Materials and Methods). Overall, all samples formed a well-supported monophyletic clade [ultrafast bootstrap support (100%)/approximate Likelihood-Ratio Test (aLRT) (87%)] clustering with an *E. rhusiopathiae* pig sample from Russia (GCF_003725505.1) (fig. S18). However, to infer a common origin for the five samples, we independently tested the positioning of each of the five branches (table S8). In three of five cases, the Las Gobas sequence was positioned next to the same pig sequence (GCF_003725505.1), indicating a single origin for at least these three Las Gobas samples (37, 34, and 32). For the other two (7A and 28), further capture sequencing would be required to confirm the *E. rhusiopathiae* specific subbranch origin with confidence.

We note that, although *E. rhusiopathiae* samples exhibited a declining edit distance (figs. S7 to S11), numerous reads had a high number of mismatches (4 to 10) for several individuals. This could be

due to ancient damage and/or false comapping of an additional strain. The latter can be attributed to the fact that the closest strain from the Las Gobas *E. rhusiopathiae*, present in the aMeta database, was quite distant in the phylogenetic analysis, comprising more *E. rhusiopathiae* samples. While the MEGAN Alignment Tool (MALT) (*30*) analyses classified the *E. rhusiopathiae* GCF_900637845.1 as the closest strain, the genome GCF_003725505.1, not present in the aMeta database, was the closest in the phylogeny (fig. S18).

Investigating under the aMeta threshold, we identified two additional pathogens, *B. recurrentis*, responsible for the louse-borne relapsing fever in individual 12, and an ancient variola virus (aVARV), the causative agent of smallpox in individual 43 (figs. S19 to S21). Both diseases have a high mortality rate, and *B. recurrentis* is still present in different parts of the world today, like the Horn of Africa, while smallpox has been eradicated (*31*). Competitive mapping supported the assignment of the reads to *B. recurrentis* in comparison to other pathogens of the Borrelia genus (table S7). A comparative edit distance between several reference genomes showed that the aVARV detected here was closest to the only available (almost) full consensus sequence for aVARV (VK382) (fig. S22) (*32*). After the second round of sequencing, we recovered 4228 reads for aVARV, summing up to a 53.4% breadth of coverage and 0.94× coverage when mapping to VK382.

To determine the phylogenetic position of aVARV, we constructed a tree incorporating the conserved regions of 90 poxviruses (see Materials and Methods) (Fig. 4 and fig. S23). The final alignment consisted of 103,504 sites and 8117 parsimony-informative sites. A total of 56,352 sites were covered by the aVARV from individual 43. While the results warrant cautious interpretation due to the low coverage of our sample, it shows that it clusters robustly with other ancient VARV strains identified in individuals buried in northern Europe between ~885 and 1000 CE (Fig. 4), regardless of the reference genome we align it to, be it VK382 (fig. S23) or the closest related virus to variola, the taterapox virus (fig. S24).

Genetic structure

We projected individuals from Las Gobas together with previously published ancient Iberian individuals (table S9) onto the first two principal components of modern West Eurasians and North African populations using the dataset 1240K+HO v.54.1.p1 (Fig. 5) (*23*). The principal components analysis (PCA) indicated genetic similarity among the two different phases of the necropolis, with most of the individuals placed within the range of the genetic variation observed in modern Spanish populations (Fig. 5). Except for two individuals displaying negative PC1 values (in the direction modern north Africans) and clustering near Islamic Iberian, south Iberian Visigoths, and Iberian Romans, the remaining individuals cluster close to Iron Age and early Medieval north Iberians (Fig. 5).

The PCA findings are supported by the unsupervised ADMIXTURE results (*33*), in which individuals from the two phases from Las Gobas lack significant differences in their ancestry components (Fig. 6 and table S10). As a whole, they exhibit an average proportion of the European-like (purple) component (87%), similar to that of Carolingian individuals (88%) and north (Catalonia) Iberian Visigoths (87%) (Fig. 6 and table S10). However, north Iberian Visigoths show slightly higher levels of the Western Asia/Caucasus-like (red) component (*P* value < 0.01) (Fig. 6 and table S10). Notably, more pronounced differences (*P* value < 0.001) are observed in the levels of North African and Western Asia/Caucasus-like components between Las Gobas individuals (8 and 4%, respectively) and Roman Iberians (17 and 16%), south Iberian Visigoths (29 and 9%), Islamic Iberians (24 and 14%), and Late Islamic Iberians (29 and 18%) (Fig. 6 and table S10).

The fact that the individuals classified as south Iberian Visigoths from the necropolis el Castillo de Montefrío (Granada) already presents high levels of North African ancestry (29%) (Fig. 6 and table S10) while being dated between 400 and 600 CE (several generations before the Umayyad conquest of Iberia) requires further explanation (*1*, *34*). Gene flow between Visigoths in southern Iberia

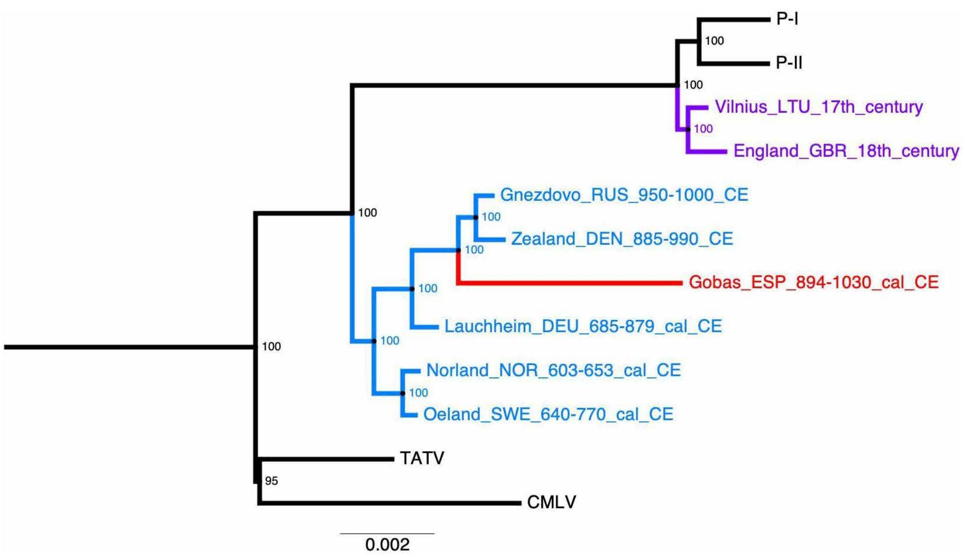

Fig. 4. Reduced maximum likelihood tree of variola virus. All samples were mapped to the consensus sequence of aVARV from a Viking from Öland (VK382). Taxon name fields indicate site/clade, three-letter country code, and date/time. Historical variola virus is shown in purple, ancient VARV in blue, and the ancient VARV from Las Gobas in red. We collapsed the modern clades and kept the closest outgroups: the taterapox virus (TATV) and the camelpox virus (CMLV) (for full tree, see fig. S22). Node confidence values (bootstrap support) are shown at each node. The scale bar shows the average number of nucleotide substitutions per site.

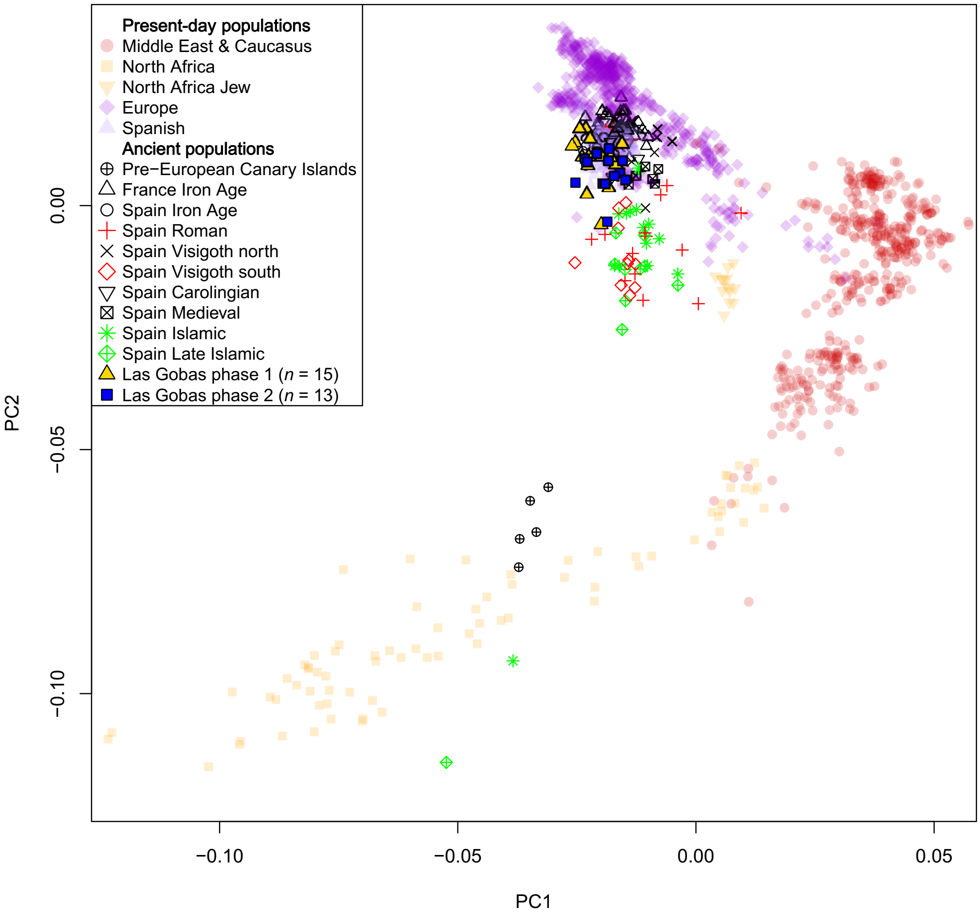

Fig. 5. PCA of Las Gobas individuals. Genetic data from Las Gobas (n = 28) and other ancient individuals projected onto the first two principal components of modern North Africans and West Eurasians.

and populations with high levels of North African ancestry (e.g., Roman elites), combined with the acculturation of the latter group, may explain the high degree of North African ancestry in early Visigoths of southern Iberia. Another explanation for the notable North African ancestry observed in some south and south-east Iberian individuals could be the earlier Punic expansion into Iberia. In addition, the long history of interactions between North African Berber communities and southern Iberia, including gradual settlement and mercantile activities, likely played an important role in shaping of the genetic landscape. This perspective is supported by extensive scholarship examining the complex processes of Islamization and settlement, suggesting gradual contacts before the Umayyad conquest of Iberia (*35*–*38*). Further archaeological and genetic research is needed to clarify these points and to explore other possible scenarios.

The two previously mentioned outliers in the PCA (individual 37, a male with the L1b1 Y chromosome haplotype from phase I dated to 773 to 892 CE, and individual 7B, a female from phase II older than 993 to 1050 CE; table S2) present some of the highest levels of North African component (23 and 13%, respectively) in the admixture analyses at K = 5 (table S10). The Umayyad conquest of southern and central parts of Iberia (711 CE) predate both individuals.

We conduct qpAdm analyses (*19*, *39*) to formally assess the extent of North African ancestry in the Las Gobas individuals. In Fig. 6B, we present the results of a two-source model, using Visigoths or Iron Age individuals from northern Iberia (*1*) and Canary Islands aborigines populations (*40*, *41*) as proxies for early Medieval northern Iberians and North Africans, respectively. This analytical approach enables us to incorporate related individuals, considering the independence of each ancient individual in the analysis.

In general, the qpAdm results align with the ADMIXTURE results, revealing relatively low levels of North African (Canary Islands aborigines) ancestry, with some individuals showing none at all (table S11). However, it is crucial to interpret these findings cautiously, as Canary Islands aborigine populations may not accurately represent the Medieval North African populations that conquered Iberia due to continuous gene flow from diverse populations in North Africa since the Canary islands were populated (*40*–*42*). Note that our qpAdm analyses exclude a population representing the Middle East/Caucasus, in contrast to the ADMIXTURE analysis where such populations were included. The exclusion is based on the unviability of models incorporating Middle East/Caucasus for most individuals. This divergence in model inclusion may contribute to potential inflation of Guanche percentages compared to ADMIXTURE results for specific individuals (see Fig. 6B).

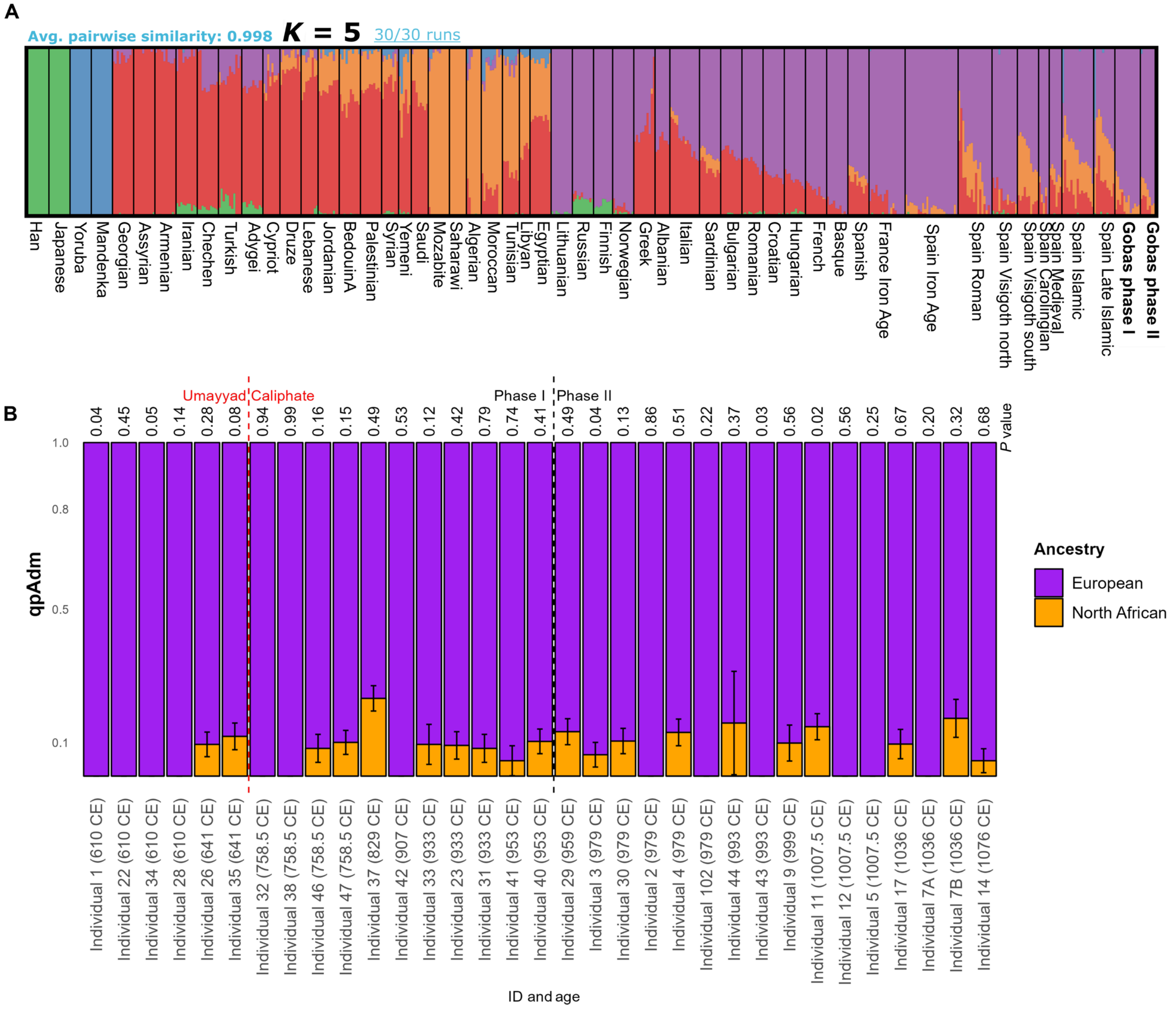

Fig. 6. Ancestry components. (**A**) ADMIXTURE analysis at $K = 5$ of 19 unrelated individuals and a selection of modern populations from the 1240K+HO dataset together with published ancient genomes (table S10). (**B**) A bar plot illustrating qpAdm results depicting the composition of European ancestry, represented by northern Iberian Visigoths or Iron Age individuals from north Iberia, and North African ancestry, represented by Canary Islands aborigines, across 33 individuals from Las Gobas. The individuals are ordered by ID and radiocarbon age on the *x* axis. The *y* axis represents qpAdm values (table S11). European and North African components are distinguished by color (purple and orange, respectively). For individuals 35 and 47, Iron Age populations from north Iberia were used instead of northern Iberian Visigoths as the latter did not provide a feasible model. Error bars indicate the upper and lower SEs. The *P* value for each analysis is displayed on the plot.

To further investigate the influence of the Islamic expansion on individuals from Las Gobas, we examined the correlation between individuals' radiocarbon ages and ancestries calculated with both methods ADMIXTURE (Fig. 7A) and qpAdm (Fig. 7B). Our findings indicate that there is no significant correlation between the age of individuals and the varying levels of these ancestries (Fig. 7).

Genomic analyses of individuals from Las Gobas, dating back to periods both before and during the Umayyad conquest, indicate that this historical event did not result in a significant increase in nonlocal genetic ancestry within this rural community over time (Fig. 7). However, we note that the six individuals exhibiting over 10% North African–like ancestry (table S10) postdate the period following the Islamic expansion in Iberia. These findings suggest that even within this North Iberian isolated rural population, there were periods of sporadic genetic exchange with populations carrying North African ancestry, with a substantial portion likely occurring during the Umayyad Caliphate. However, the detection of minor levels (<10%) of North African ancestry in certain individuals from Las Gobas predating the Islamic conquest of Iberia, coupled with the observation that Roman Iberians, and although in a

limited proportion (~4%) even Iron Age Iberians, also exhibit North African ancestry, prevents us from conclusively determining the timing and source of the North African genetic influence on individuals from Las Gobas.

To test population affinities between the individuals from Las Gobas and other groups, we divided the ancient individuals in the two different phases, removed one individual from each pair of related individuals, and performed *f*-statistics analysis. The multidimensional scaling (MDS) of pairwise f_3 results indicated that individuals from the two phases shared most genetic affinities (Fig. 8 and table S12), suggesting that Las Gobas forms a homogeneous population, likely as a result of population continuity across the centuries with relatively low levels of nonlocal gene flow. Dimension 1 of the f_3 MDS plot is probably mainly driven by an increase in North African and Middle East ancestries from left to right.

DISCUSSION

The borders of the different Islamic and Christian governments fluctuated drastically during the almost eight centuries that parts of Iberia were under Islamic rule (*7*). Over extended periods, the frontier with the Islamic-ruled territories remained proximate to Las Gobas (Fig. 1B), with well-documented Islamic influence extending as far as Pamplona, located 100 km to the northeast (*43*, *44*). However, the individuals buried at Las Gobas present levels of North African ancestry comparable to pre-Roman Iron Age Iberians, north Iberian Visigoths, and Carolingians (groups that did not have apparent interactions with North African populations). Notably, these levels are significantly lower than those observed in individuals from Roman sites, south Iberian Visigoths, and Islamic Iberians. This observation implies that the Roman influence before and the Islamic influence during the five centuries covered by the Las Gobas record did not have a substantial

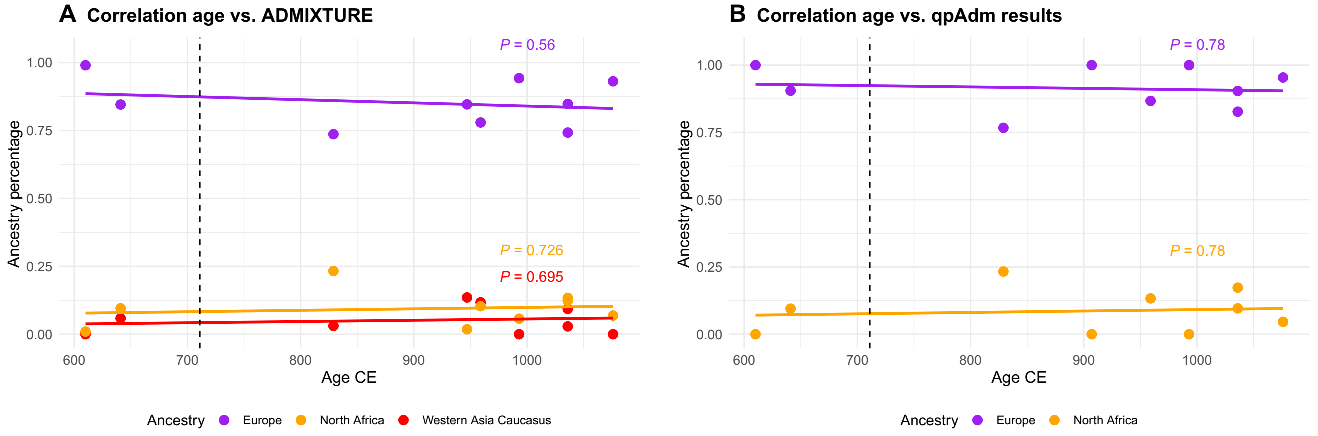

Fig. 7. Correlation between age and ancestry. Linear regression lines (solid lines) provide an overview of the trends. The *P* values for each ancestry's correlation are annotated on the plot. Related individuals and individuals that cannot be linked to a direct radiocarbon date were removed from these analyses. The vertical dashed line indicates the beginning of the Umayyad Caliphate. (**A**) The scatterplot illustrates the relationship between age (in years) and the percentage of Western Asia Caucasus (red), Europe (purple), and North Africa (orange) ancestries from the ADMIXTURE results (table S10). (**B**) The scatterplot illustrates the relationship between age (in years) and the percentage of Europe represented by north Iberian Visigoths (purple) and North Africa represented by Canary Islands aborigines (orange) ancestries from the qpAdm results (table S11).

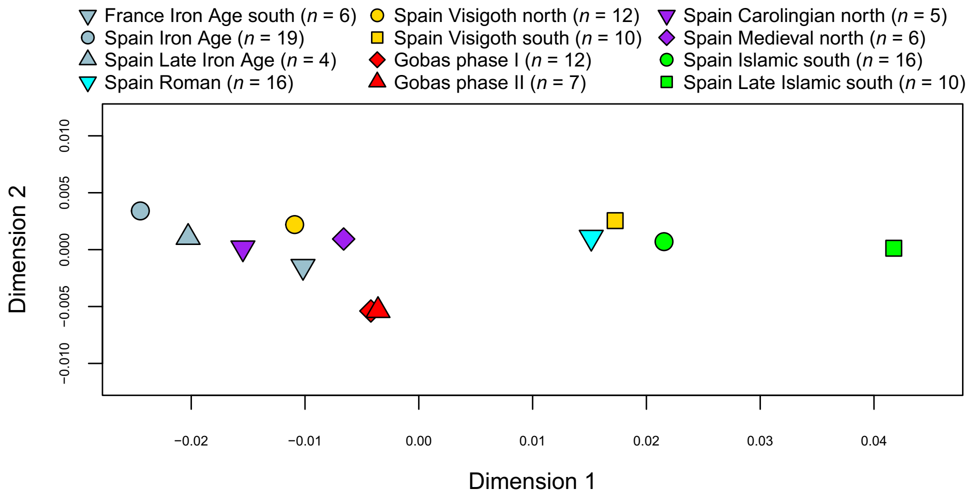

Fig. 8. *f*3-statistic MDS plot. Pairwise genetic distance matrix from inverted *f*3 values (1-*f*3) of the form *f*3 (ancient group A and ancient group B; Mbuti) between ancient Iberian groups from different periods and cultures (goodness of fit $r^2 = 0.999$).

impact on the genetic composition of this small Christian community. These findings agree with the historical sources and modern genetic data (*45*), which suggest that the northern regions of the Iberian Peninsula, particularly the Basque region, experienced comparatively lower genetic influence from North African populations during the Middle Ages and the Roman era. However, it is important to note that Roman Iberians used here as comparative data refer to individuals found in Roman sites within Iberia (*1*) and probably represent the diversity of the vast Roman Empire populations rather than local Iberians during the Roman Age (table S9).

The presence of consanguinity and violence in the early stages of the necropolis, together with the relatively small variations observed in the Y chromosome, leads to speculation about the possibility that the site was populated in the seventh century CE by a small patrilocal endogamous group that could be, for example, members of an elite with military experience. Conversely, changes in the second phase, where we have identified more kinship connections and an absence of evident sword injuries, suggest that the site has probably changed its character to that of a rural farming necropolis. In addition, there is a slight decrease in the number of individuals with high levels of inbreeding in this later phase of the Necropolis (Fig. 3 and table S5), which may reflect changes in social organization. However, we have documented genetic continuity between the two phases, and our results suggest the practice of endogamy and close interactions with animals over the five centuries under study. The geographic location of Las Gobas, surrounded by mountains and relatively distant from urban centers, likely contributes to the community's isolation. The observed high levels of consanguinity among some individuals from Las Gobas indicate cultural practices of endogamy, which could also limit the gene flow from neighboring communities. This local isolation may, over time, also result in reduced gene flow from Arabs and North Africans on a broader scale.

The metagenomic results suggest close interactions with animals, as the majority of infectious diseases detected within the Las Gobas community were zoonoses. The most common was *E. rhusiopathiae*, a skin infection that usually arises when handling meat while having open cuts (*46*, *47*). Two hypotheses can explain its presence in Las Gobas human remains: (i) It originated from the necrobiome; however, studies suggest limited soil persistence (*48*, *49*); (ii) it was a zoonotic transmission from infected animals, which is a more likely scenario. However, while the closest taxa from the five samples was a porcine strain from Russia, the origin of the host remains unclear as no strict host specificity has been described for this species (*41*).

The discovery of *Y. enterocolitica*, a food-borne pathogen, agent of yersiniosis and often associated with undercooked pork consumption, indicates the presence of this pathogen in at least one individual consistent with previous findings in ancient human remains (*50*, *51*). Other zoonotic pathogens detected in Las Gobas were *B. recurrentis*, transmitted by the human body louse, and leptospirosis, caused by *L. interrogans*. Leptospirosis is generally acquired through contact with water contaminated by the urine of infected animals.

In addition to exploring the genetic composition and health conditions of the Las Gobas community, our study confirms the pan-European distribution of smallpox in the Middle Ages (*32*) and provides valuable insights into the intriguing historical puzzle of how it entered Europe. Smallpox, with its devastating impact, poses a complex challenge in tracing its origins and spread throughout the continent. Given the infectivity and long incubation time of the disease, it is likely that smaller-scale events might have brought it to different parts of Europe at different times. However, if the spread of smallpox intensified with increased human mobility, then specific scenarios can be explored in the light of recent archaeogenetic findings. For example, the presence of aVARV in individuals buried in northern Europe during the seventh century (*32*, *52*), before the Viking Age and predating the Umayyad invasion of 710 CE, suggests that the virus was already present in Europe before the Islamic conquest of Iberia. Previous hypotheses that linked the presence of the virus in Europe with the migrations from the east around the fifth and sixth centuries better fit the archaeogenetic data (*32*, *53*). The phylogenetic clustering of the aVARV found in the individual 43 from Las Gobas with those discovered in northern European individuals, dating back to between 885 and 1000 CE, suggests that the virus may have reached the Las Gobas individual via Europe rather than through Islamic routes, which was one of the dominant ideas for how smallpox entered Iberia (*54*). Whether the Umayyad invasion was an additional factor in the spread of smallpox will eventually be clarified with early medieval VARV genomes from individuals buried in North Africa and the Middle East.

The individual infected with the aVARV virus was also afflicted by leptospirosis. Coinfections involving two different pathogens, like the one observed in individual 32 with both *E. rhusiopathiae* and *Y. enterocolitica*, were likely as common in the past as they are today (*55*).

By integrating genetic, archaeological, and historical data, we unveil the presence of an endogamous and consanguineous community in Northern Iberia. Despite centuries of turbulent regional history, this community experienced limited external gene flow. Our findings indicate that this community remained relatively isolated for at least five centuries. Last, the metagenomic analyses of the Las Gobas individuals integrated with the rest of the information gathered in this study complements the understanding of the health condition of this rural community. By focusing on site-specific pathogens, we provide a detailed view of the disease landscape at a specific time and place while also highlighting previously unreported ancient pathogens, such as *E. rhusiopathiae* and *L. interrogans*. To corroborate certain assertions made in this study and pinpoint the source and timing of North African ancestry in Iberian Peninsula populations across various epochs, additional ancient genomes from local Iberians during the Roman period, and particularly North African genomes spanning the Roman era to the 11th centuries CE, will be essential.

Last, while we can identify the detected pathogens and confirm their ancient status, their low coverage sometimes limits our capacity for further phylogenetic and gene evolution analyses. In addition, in *L. interrogans*, the observed nonperfectly decreasing edit distance pattern may reflect evolutionary differences between the modern reference genome and the ancient strains and/or the lack of appropriate reference genomes in the database. We note that despite the various statistics and quality controls shown in the study supporting the presence of *B. recurrentis* in individual 12 and *L. interrogans* in individual 43, the low coverage prevents us from confirming their presence with absolute confidence.

MATERIALS AND METHODS

DNA extraction

The human remains were sampled in the aDNA facilities at the Archaeological Research Laboratory, Stockholm University (Sweden).

All samples were decontaminated before analysis with ultraviolet irradiation (6 J/cm^2 at 254 nm). After removing the surface, bone was drilled to powder, and the root tip of the teeth was cut with a multitool drill (Dremel) to get approximately 80 to 150 mg of bone powder/root tip.

The root tip samples were placed in an Eppendorf tube in 1 ml of predigestion buffer [0.45 M EDTA (pH 8.0)] at 37°C in a hybridization oven with rotation. After 30 min, the supernatant was removed to reduce the exogenous DNA contamination. Following this predigestion step, 1 ml of extraction buffer [0.45 M EDTA (pH 8.0) and proteinase K (0.25 mg/ml)] was added to all the samples, and they were incubated at 37°C in the hybridization oven with rotation for 1 to 4 days until all powder/root tip was dissolved. The extraction was conducted following (*56*), with 1 ml of digested extract being combined with 13 ml of binding buffer containing 5 M guanidine hydrochloride, 40% (v/v) isopropanol, 0.05% Tween 20, and 90 mM sodium acetate (pH 5.2). Fifty-milliliter silica columns (Roche, High Pure Viral Nucleic Acid Large Volume Kit) were used for purification, and the DNA was eluted in 45 μl of Elution Buffer (EB; Qiagen). Blank controls were used along all extraction steps.

Library preparation and sequencing

Twenty microliters of extract was used to prepare blunt-end ligation DNA libraries coupled with P5 and P7 adapters and double indexes as described in (*57*). Blank controls were used during every step of library preparation and amplification. The optimal number of polymerase chain reaction (PCR) cycles for library amplification was determined with quantitative PCR. The amplification reactions had a total volume of 50 μl, with 5 μl of DNA library, and the following in final concentrations: 1× AmpliTaq Gold Buffer, 2.5 mM $MgCl_2$, 25 μM of each deoxynucleotide triphosphate, 2.5 U of AmpliTaq Gold (Thermo Fisher Scientific, Waltham, MA), and 200 nM of each of the index primers (Meyer and Kircher, 2010). PCR was done with the following conditions: an activation step at 94°C for 10 min followed by 8 to 20 cycles of 94°C for 30 s, 60°C for 30 s, and 72°C for 45 s and a final elongation step of 72°C for 10 min. Four amplification reactions with the same indexing primer were made for each library to increase complexity. Last, the amplified libraries were pooled and purified with AMPure XP beads (Agencourt; Beckman Coulter, Brea, CA), and the fragment size and concentration were checked using BioAnalyzer with the High Sensitivity Kit (Agilent Technologies, Cary, NC).

Processing and alignment of sequencing reads

Purified libraries were pooled in equimolar concentration and sequenced on an Illumina HiSeq X10 and/or on NovaSeq 6000 at the SciLifeLab National Genomics Infrastructure in Stockholm. Sequencing reads were demultiplexed according to the index of each sample sequence. Cutadapt v. 2.3 was used for trimming adapters and FLASH v. 1.2.11 for merging of fastq reads. The reads were mapped against human reference genome build 37 (hs37d5) using the Burrows-Wheeler Algorithm, as implemented by Burrows-Wheeler Alignment (BWA) v. 0.7.17, (*58*) with the following parameters aln (-l 16500 -n 0.01 -o 2). The fastq files from different sequencing runs of the same library were merged using Samtools v. 1.17. merge option (*59*). Next, a slightly modified version of FilterUniqueSAMCons.py (*60*) was used to condense the reads with identical start and end position into a consensus read. Last, reads shorter than 35 bp and reads with less than 90% identity with the reference were filtered out using percidentity_threshold.py (*61*). Because of the low coverage of the ancient samples, we built pseudo-haploid genomes by randomly choosing one read with a minimum mapping quality of 30 and a base quality of 30.

Data validation

All libraries yielded short read lengths, and patterns of cytosine deamination were estimated using PMDtools (table S1) (*62*). We have applied two methods based on the mitochondrial DNA (mtDNA) to estimate contamination in our samples (*63*, *64*). In addition, we used the "Contamination" program in ANGSD (*65*) v.0.911 to estimate X chromosome contamination in males, as described in (*66*). Contamination estimates are shown in table S2.

Metagenomic analysis

Metagenomic analysis of the DNA libraries was performed using aMeta v.1.0.0 (*24*) which yielded authentication scores for each detected microbial organism and authentication plots. We then retained microbes with an authentication score of ≥8 as a proxy for true presence and ancient status. We assigned the status of environmental contaminants to microbes from various isolation sources, including soil, storage, and lab contamination referenced in the BacDive database (*67*) or presence in more than 1% of soil samples (excluding farm and unknown types) from the Microbe Atlas Project Database (*68*). Several bacteria were further excluded on the basis of the literature (*69*, *70*) or due to insufficient information. We further classified the remaining microbes as pathogens or as part of the human oral microbiome, the latter based on the expanded Human Oral Microbiome Database and the literature (*26*–*28*). To evaluate *Y. enterocolitica*'s pathogenicity, we aligned it to its nearest reference genome per KrakenUniq (*25*) (type O:5, strain YE53/03, reference: NZ_HF571988.1) and visualized the coverage of genes of virulence *inv*, *yst*, and *myf* using Integrative Genomics Viewer (IGV; figs. S15 to S17) (*71*).

In addition, we assessed the presence of pathogens below aMeta default thresholds by calculating *k*-mers to read ratios, using this as a proxy for evenness of coverage and filtering for known pathogenic species. After alignment with bwa aln v.0.7.17 and removal of reads shorter than 30 bp and duplicates, we generated coverage, edit distance, and damage pattern plots using aDNA-BAMPlotter (*72*) and MapDamage v.2.2.2 (*73*) for the pathogen hits showing the best ratios, namely, *B. recurrentis* and variola virus.

Last, we confirmed bacterial pathogens identification through competitive mapping (table S7). For the species detected with aMeta, we extracted species-specific reads with MaltExtract v.1.7 with the options --destackingOff and --downSampOff to retain all reads (*74*). We then aligned each pathogen hit with Bowtie 2 v.2.5.2 (*75*) against databases containing the first representative assembly from NCBI for each species of their respective genus.

Phylogenetic analyses

To determine the phylogenetic position of the detected aVARV, we constructed a tree incorporating the conserved regions of 90 poxviruses based on the multiple sequence alignment from (*32*, *76*). We completed it with more recent discoveries, one historical VARV from 18th century England (*77*) and one aVARV from Early Medieval Germany (*52*). We preprocessed these new samples using fastp v.0.23.4 (*78*) for adapter removal, merged sequences with

FLASH v.1.2.11 (*79*), and conducted quality checks with FastQC v.0.11.9 (*80*). We then aligned these samples and our sample to VK382, a Viking aVARV strain from Sweden (GCA_903469545) (*32*), modern variola virus (NC_001611.1), and taterapox virus (NC_008291.1), using bwa aln v.0.7.17 (bwa aln -n 0.01 -l 16500 -o 2). We identified the closest reference genome for the aVARV strain through edit distance comparison among these genomes. Before generating consensus sequences, we filtered the BAM files for a mapping quality and read length of 30, removed duplicates using samtools markdup v.1.19 (*81*), and minimized postmortem damage by soft clipping 10 bp on both sides of the reads with BamUtil v.1.0.15 (*82*). We then generated consensus sequences using ANGSD v.0.940 with a minimum depth of 1 (*65*). Last, we generated a maximum likelihood tree using IQ-Tree v2.2.2.6 (*83*), with 100 classical bootstraps (option -b 100). The implemented program ModelFinder (*84*) identified the best substitution model according to Bayesian Information Criterion (BIC) as TVM + F + I + R4 (option -m MFP), and the tree was visualized using the Figtree software (http://tree.bio.ed.ac.uk/software/figtree/).

To generate a phylogenetic tree for *Erysipelothrix* species, we first extracted all reads from seven human samples that were classified as *E. rhusiopathiae* with MaltExtract. Next, we downloaded all available *E. rhusiopathiae* ($n = 46$), *Erysipelothrix inopinata* ($n = 1$), and *Erysipelothrix tonsillarum* ($n = 1$) assemblies (not including the ones removed by NCBI) (download: 19 March 2024) and aligned the modern genomes together using Sibeliaz v1.2.5 (*85*) (-k15). The extracted reads were mapped to their closest reference genome (based on the result of the competitive mapping), namely, *E. rhusiopathiae* (GCA_900637845) for individuals 7A, 32, 28, 34, and 37; *E. inopinata* (GCF_014396165) for individual 43; and *E. tonsillarum* (GCF_000373785.1) for individual 14. Mapped reads were filtered using the same parameters as for aVARV. The final alignment consisted of 2,415,066 sites and 41,700 parsimony-informative sites. The number of sites covered by the seven ancient *Erysipelothrix* candidate species ranged from 8505 (individual 14) to 1,357,672 (individual 37) sites. Last we generated a maximum likelihood tree using IQ-Tree v2.2.2.6 (*83*), with 1000 UFboot and SH-aLRT bootstraps (options -bb 1000 -alrt 1000) (*83*). Values of UFboot ≥ 95% and SH-aLRT ≥ 80% were considered as reliable as recommended by IQ-Tree authors. The implemented program ModelFinder (*84*) identified the best substitution model according to Bayesian Information Criterion (BIC) as TVM + F + I + R4 (option -m MFP). To rule out an attraction effect between the five Las Gobas *E. rhusiopathiae* hits, a phylogeny of each of them was inferred. This was performed by building five independent phylogenies with the same parameters as the general phylogeny but including only one Las Gobas *E. rhusiopathiae* sequence per phylogeny (see all newick files in table S8).

Population genomics

Datasets

We used TrimBam of BamUtils (*82*) to trim 10 bp at the end of each read in our ancient individuals to remove the damage patterns characteristic of ancient and degraded DNA. Next, we merged the ancient samples with two different datasets from (*23*) version 54.1.p1: (i) "1240K+HO" and (ii) "1240K." In the ADMIXTURE and PCA analyses, we use a pseudohaploidized version of the dataset to mitigate bias when analyzing both haploid (ancient) and diploid (modern) individuals.

We applied additional filtering for the PCA analyses, excluding five individuals with genome coverage < 0.17 (table S1), resulting in 28 remaining individuals. In the ADMIXTURE and *f*-statistic analyses, we further exclude nine individuals; one with the lowest coverage from each pair of genetically related individuals with a k_0 value < 0.8 and pi_HAT > 0.06 (~third-degree kinship) (table S2). Therefore, 19 unrelated individuals were used in the ADMIXTURE and *f*-statistic analyses.

Mitochondrial haplogroups

We used Samtools v. 1.17 (*59*) to filter out mtDNA reads with mapping and base quality of minimum 30. Next, we used bcftools to call the consensus and generated fasta files using consfastQ2fasta.py. Last, we use HaploGrep 2.1.16 (*86*, *87*) to assign mtDNA haplogroups based on PhyloTree Build 17 phylogeny (table S2) (*88*).

Y chromosome haplogroups

We determined Y chromosome haplogroups in the male individuals using pathPhynder v.1a (*89*). The "BigTree" Y chromosome dataset (included with pathPhynder) was used as the reference phylogeny, minimum base quality was set to 30, and maximum tolerance was set to 100. Both "default" and "transversions" filtering modes were used, which generated comparable results (table S3).

Regions of homozygosity

We used the hapROH software (*22*) to compute and plot the ROH, adhering to the guidelines provided by the authors (https://pypi.org/project/hapROH/).

Kinship analysis

We inferred genetic kinship for all pairs of the Las Gobas individuals using genomic data in combination with additional information (e.g., uniparental markers) and using four different methods: lcMLkin (*90*), KIN (*91*), NgsRelate (*92*), and LocalNgsRelate (*93*) as described below. We jointly analyzed the results of the four software to assess consistency among the outcomes, using this as a confidence measure for kinship estimation. We used a SNP panel of 1,681,497 transversion SNPs from the Estonian Genome Diversity Project (EGDP) (*94*) for the autosomal kinship analysis. For X chromosome–based kinship estimation, we again included only transversion SNPs from the EGDP dataset, with at least 5% minor allele frequency (MAF) and filtered the pseudo-autosomal regions from the X chromosomes as described in the human reference genome (hs37d5). After this filtering, 74,045 SNPs remained.

lcMLkin

We estimated kinship relations between the individuals analyzed in this study using the software lcMLkin (*90*) and our BAM files. We selected the SNPs from the EGDP (*94*) with a MAF greater than or equal to 0.15 and using only the transversions to avoid postmortem damage bias in our ancient samples. The genotype likelihoods of the selected SNP positions were called with "SNPbam2vcf.py" (*90*) using the population allele frequencies from the Las Gobas individual plus 104 present-day Spanish individuals (IBS) from the 1K genomes phase 3 (*95*). Following the developer recommendations, we first run lcMLkin assuming all individuals are unrelated to detect pairs of related individuals. Then, we removed all related and ancient individuals and run it again to obtain more reliable estimates of kinship coefficients using the founder flag -u with only the modern Spanish individuals.

KIN
As a second approach, we used the software KIN (*91*) which estimates the genetic kin relationship between individuals using a hidden Markov model–based approach. This software differentiates between parent-offspring and siblings and also considers inbreeding from runs of homozygosity (ROH) while classifying relatedness between individuals. KIN does not depend on population allele frequencies which distinguishes it from the other three tools. The software estimates the most likely degree of relatedness based on the highest likelihood. We run KIN with default parameters, using BAM files as input.

NgsRelate
We estimated the autosomal kinship coefficient ($\theta = k_1/4 + k_2/2$) and the probabilities of sharing 0, 1, and 2 alleles Identical by Descent (IBD) between each pair of the Las Gobas individuals (Cotterman coefficients, $k_0 + k_1 + k_2 = 1$) using the NgsRelate software (*92*). For this, we used BAM files as input and ran NgsRelate with default parameters, and population allele frequencies were calculated from $n = 33$ Las Gobas individuals (table S2). We restricted the analysis to a minimum of 5000 overlapping SNPs for a pair of individuals which was earlier suggested as a measure of confidence for kinship estimation down to third-degree (*96*).

LocalNgsRelate
LocalNgsRelate (*93*) also applies a maximum likelihood approach, using genotype likelihoods and population allele frequencies, similar to NgsRelate. The authors suggest that it can accurately estimate kinship from low coverage genomic data and is used to identify pedigree relationships between the putative kin pairs inferred by NgsRelate. We implemented LocalNgsRelate on BAM files as input with default parameters and used population allele frequencies calculated from Las Gobas individuals ($n = 33$; table S2). We computed 100 optimization rounds for each related pair using the parameter -N 100.

Kinship estimation on the X chromosome
We further estimated the X chromosomal kinship coefficient to infer different pedigree relationships between the related pairs (e.g., siblings, mother-son, and father-daughter). For this, we used NgsRelate with the X chromosome SNP panel, following the same method described above. Last, we combined kinship coefficients calculated from autosomal and X chromosome loci with the uniparental markers to identify the most probable pedigree relationships between related pairs.

Principal components analysis
Principal components were calculated on the basis of 1126 modern-day individuals from different populations from North Africa, Middle East, Caucasus, and Europe from 1240K+HO dataset (*23*) using the smartpca module in EIGENSOFT (v.6.0.1) (*97*) with options lsqprojec and shrinkmode set to YES, thus enabling projection of ancient individuals onto PC space calculated using modern reference data.

Admixture estimations
Unsupervised ADMIXTURE v.1.3 (*33*) was run on 135 ancient samples and 397 modern individuals from the dataset 1240K+HO (table S9) (*23*). We excluded one pair of each related individual up to third-degree relation. The dataset underwent pruning for linkage disequilibrium between markers with Plink v1.90 –indep-pairwise 200 25 0.4 (*98*), resulting in a final set of 494,315 SNPs used for calculating ADMIXTURE proportions. The results were parsed, aligned, and plotted with PONG (*99*).

qpAdm
Admixture modeling was performed with qpAdm_wrapper (https://github.com/pontussk/qpAdm_wrapper) and Admixtools v5.0 (*19*, *100*). We test different combinations of individuals and/or populations from the 1240K dataset using as references (or right populations) the set define in (*101*) (Russia_Ust_Ishim.DG, Russia_Kostenki14.SG, Russia_MA1_HG.SG, Han.DG, Papuan.DG, ONG.SG, Chukchi.DG, Karitiana.DG, and Mbuti.DG) and one and/or two source models using different populations [north_Spain_Visigoths (*1*), north_Spain_IA (*1*, *102*), Basque.DG, Canary Islands aborigines (*40*, *41*), and Mozabite.DG] (table S11). Aboriginal Canary individuals from (*41*) were processed in the same manner as our Las Gobas individuals and subsequently merged with the 1240K dataset. All feasible models with P values > 0.01 are displayed in table S11. Figures 6B and 7B show the results obtained using North Iberian Visigoths and Guanches as either single or double sources. When both single and double source models are feasible, we selected the model with the highest P value (see complete results in table S11).

f-Statistics
Patterson's f_3-statistics were calculated using the ADMIXTOOLS package v. 3.0 (*39*). We excluded one pair of each related individual up to third-degree relation.

Supplementary Materials
The PDF file includes:
Figs. S1 to S24
Legends for tables S1 to S12
References

Other Supplementary Material for this manuscript includes the following:
Tables S1 to S12

REFERENCES AND NOTES
1. I. Olalde, S. Mallick, N. Patterson, N. Rohland, V. Villalba-Mouco, M. Silva, K. Dulias, C. J. Edwards, F. Gandini, M. Pala, P. Soares, M. Ferrando-Bernal, N. Adamski, N. Broomandkhoshbacht, O. Cheronet, B. J. Culleton, D. Fernandes, A. M. Lawson, M. Mah, J. Oppenheimer, K. Stewardson, Z. Zhang, J. M. Jiménez Arenas, I. J. Toro Moyano, D. C. Salazar-García, P. Castanyer, M. Santos, J. Tremoleda, M. Lozano, P. García Borja, J. Fernández-Eraso, J. A. Mujika-Alustiza, C. Barroso, F. J. Bermúdez, E. Viguera Mínguez, J. Burch, N. Coromina, D. Vivó, A. Cebrià, J. M. Fullola, O. García-Puchol, J. I. Morales, F. X. Oms, T. Majó, J. M. Vergès, A. Díaz-Carvajal, I. Ollich-Castanyer, F. J. López-Cachero, A. M. Silva, C. Alonso-Fernández, G. Delibes de Castro, J. Jiménez Echevarría, A. Moreno-Márquez, G. Pascual Berlanga, P. Ramos-García, J. Ramos-Muñoz, E. Vijande Vila, G. Aguilella Arzo, Á. Esparza Arroyo, K. T. Lillios, J. Mack, J. Velasco-Vázquez, A. Waterman, L. Benítez de Lugo Enrich, M. B. Sánchez, B. Agustí, F. Codina, G. de Prado, A. Estalrrich, Á. F. Flores, C. Finlayson, G. Finlayson, S. Finlayson, F. Giles-Guzmán, A. Rosas, V. B. González, G. G. Atiénzar, M. S. H. Pérez, A. Llanos, Y. C. Marco, I. C. Beneyto, D. López-Serrano, M. S. Tormo, A. C. Valera, C. Blasco, C. Liesau, P. Ríos, J. Daura, M. J. de Pedro Michó, A. A. Diez-Castillo, R. F. Fernández, J. F. Farré, R. Garrido-Pena, V. S. Gonçalves, E. Guerra-Doce, A. M. Herrero-Corral, J. Juan-Cabanilles, D. López-Reyes, S. B. McClure, M. M. Pérez, A. O. Foix, M. S. Borràs, A. C. Sousa, J. M. V. Encinas, D. J. Kennett, M. B. Richards, K. W. Alt, W. Haak, R. Pinhasi, C. Lalueza-Fox, D. Reich, The genomic history of the Iberian Peninsula over the past 8000 years. *Science* **363**, 1230–1234 (2019).
2. G. Gonzalez-Fortes, F. Tassi, E. Trucchi, K. Henneberger, J. L. A. Paijmans, D. Dıez-del-Molino, H. Schroeder, R. R. Susca, C. Barroso-Ruız, F. J. Bermudez, C. Barroso-Medina, A. M. S. Bettencourt, H. A. Sampaio, A. Grandal-d'Anglade, A. Salas, A. de Lombera-Hermida, R. F. Valcarce, M. Vaquero, S. Alonso, M. Lozano, X. P. Rodrıguez-Alvarez, C. Fernandez-Rodrıguez, A. Manica, M. Hofreiter, G. Barbujani, A western route of prehistoric human migration from Africa into the Iberian Peninsula. *Proc. R. Soc. B.* **286**, 20182288 (2019).

3. R. Fregel, F. L. Mendez, Y. Bokbot, D. Martin-Socas, M. D. Camalich-Massieu, J. Santana, J. Morales, M. C. Avila-Arcos, P. A. Underhill, B. Shapiro, G. L. Wojcik, M. Rasmussen, A. E. R. Soares, J. Kapp, A. Sockell, F. J. Rodriguez-Santos, A. Mikdad, A. Trujillo-Mederos, C. D. Bustamante, Ancient genomes from North Africa evidence prehistoric migrations to the Maghreb from both the Levant and Europe. *Proc. Natl. Acad. Sci. U.S.A.* **115**, 6774–6779 (2018).
4. L. G. Simões, T. Günther, R. M. Martínez-Sánchez, J. C. Vera-Rodríguez, E. Iriarte, R. Rodríguez-Varela, Y. Bokbot, C. Valdiosera, M. Jakobsson, Northwest African Neolithic initiated by migrants from Iberia and Levant. *Nature* **618**, 550–556 (2023).
5. P. Moorjani, N. Patterson, J. N. Hirschhorn, A. Keinan, L. Hao, G. Atzmon, E. Burns, H. Ostrer, A. L. Price, D. Reich, The history of african gene flow into Southern Europeans, Levantines, and Jews. *PLOS Genet.* **7**, e1001373 (2011).
6. M. Silva, G. Oteo-García, R. Martiniano, J. Guimarães, M. Von Tersch, A. Madour, T. Shoeib, A. Fichera, P. Justeau, M. G. B. Foody, K. McGrath, A. Barrachina, V. Palomar, K. Dulias, B. Yau, F. Gandini, D. J. Clarke, A. Rosa, A. Brehm, A. Flaquer, T. Rito, A. Olivieri, A. Achilli, A. Torroni, A. Gómez-Carballa, A. Salas, J. Bryk, P. W. Ditchfield, M. Alexander, M. Pala, P. A. Soares, C. J. Edwards, M. B. Richards, Biomolecular insights into North African-related ancestry, mobility and diet in eleventh-century Al-Andalus. *Sci. Rep.* **11**, 18121 (2021).
7. F. García Fitz, *La guerra contra el Islam peninsular en la Edad Media* (Madrid, Editorial Síntesis, 2019), pp. 278.
8. L. A. G. Moreno, *España 702–719: La Conquista Musulmana* (Universidad de Sevilla, 2013).
9. A. García Sanjuán, *La conquista islámica de la península ibérica y la tergiversación del pasado: Del catastrofismo al negacionismo* (Marcial Pons Historia, 2013), pp. 1–496.
10. A. Marsham, *The Umayyad World* (Routledge, 2020).
11. C. Bycroft, C. Fernandez-Rozadilla, C. Ruiz-Ponte, I. Quintela, Á. Carracedo, P. Donnelly, S. Myers, Patterns of genetic differentiation and the footprints of historical migrations in the Iberian Peninsula. *Nat. Commun.* **10**, 551 (2019).
12. A. Azkarate, Arqueología cristiana de la antigüedad tardía en Álava, Guipúzcoa y Vizcaya. *Diputacion Foral Alava* (Servicio de Publicaciones,1988).
13. A. Azkarate, J. Solaun, Excavaciones arqueológicas en el exterior de los conjuntos rupestres de Las Gobas (Laño, Burgos). *Arch. Esp. Arqueol.* **81**, 133–149 (2008).
14. A. Azkarate, J. L. Solaun, "Espacios domésticos, urbanos y rurales, de época medieval en el País Vasco" in *La Casa Medieval En La Península Ibérica* (Sílex, 2015), pp. 541–576.
15. I. Guede, L. A. Ortega, M. C. Zuluaga, A. Alonso-Olazabal, X. Murelaga, J. L. Solaun, I. Sanchez-Pinto, A. Azkarate, Isotopic evidence for the reconstruction of diet and mobility during village formation in the Early Middle Ages: Las Gobas (Burgos, northern Spain). *Archaeol. Anthropol. Sci.* **10**, 2047–2058 (2018).
16. L. Herrasti, I. Sánchez-Pinto, I. Etxeberria, A. Azkarate, F. Etxeberria, "Traumatic pathology and violence between the 7th and 11th centuries in the Hermitic Necropolis of Las Gobas (Laño, Treviño, Burgos)" in *Bioarchaeology of Injuries and Violence in Early Medieval Europe* (BAR Publishing, 2022), pp. 155–169.
17. P. Skoglund, J. Storå, A. Götherström, M. Jakobsson, Accurate sex identification of ancient human remains using DNA shotgun sequencing. *J. Archaeol. Sci.* **40**, 4477–4482 (2013).
18. P. Balaresque, G. R. Bowden, S. M. Adams, H.-Y. Leung, T. E. King, Z. H. Rosser, J. Goodwin, J.-P. Moisan, C. Richard, A. Millward, A. G. Demaine, G. Barbujani, C. Previderè, I. J. Wilson, C. Tyler-Smith, M. A. Jobling, A predominantly neolithic origin for european paternal lineages. *PLOS Biol.* **8**, e1000285 (2010).
19. W. Haak, I. Lazaridis, N. Patterson, N. Rohland, S. Mallick, B. Llamas, G. Brandt, S. Nordenfelt, E. Harney, K. Stewardson, Massive migration from the steppe was a source for Indo-European languages in Europe. *Nature* **522**, 207–211 (2015).
20. D. E. Platt, H. Artinian, F. Mouzaya, W. Khalil, F. G. Kamar, E. Matisoo-Smith, F. Calafell, N. N. Taleb, P. Zalloua, Autosomal genetics and Y-chromosome haplogroup L1b-M317 reveal Mount Lebanon Maronites as a persistently non-emigrating population. *Eur. J. Hum. Genet.* **29**, 581–592 (2021).
21. F. C. Ceballos, P. K. Joshi, D. W. Clark, M. Ramsay, J. F. Wilson, Runs of homozygosity: Windows into population history and trait architecture. *Nat. Rev. Genet.* **19**, 220–234 (2018).
22. H. Ringbauer, J. Novembre, M. Steinrücken, Parental relatedness through time revealed by runs of homozygosity in ancient DNA. *Nat. Commun.* **12**, 5425 (2021).
23. S. Mallick, A. Micco, M. Mah, H. Ringbauer, I. Lazaridis, I. Olalde, N. Patterson, D. Reich, The Allen Ancient DNA Resource (AADR): A curated compendium of ancient human genomes. *Sci. Data* **11**, 182 (2024).
24. Z. Pochon, N. Bergfeldt, E. Kırdök, M. Vicente, T. Naidoo, T. Van Der Valk, N. E. Altınışık, M. Krzewińska, L. Dalén, A. Götherström, aMeta: An accurate and memory-efficient ancient metagenomic profiling workflow. *Genome Biol.* **24**, 242 (2023).
25. F. P. Breitwieser, D. N. Baker, S. L. Salzberg, KrakenUniq: Confident and fast metagenomics classification using unique k-mer counts. *Genome Biol.* **19**, 198 (2018).
26. R. F. Irby, M. Kandula, R. Zadikany, R. L. Sandin, J. N. Greene, Yarrowia lipolytica as normal human flora. *Infect. Dis. Clin. Pract.* **22**, 207–209 (2014).
27. I. F. Escapa, T. Chen, Y. Huang, P. Gajare, F. E. Dewhirst, K. P. Lemon, New insights into human nostril microbiome from the expanded human oral microbiome database (eHOMD): A resource for the microbiome of the human aerodigestive tract. *mSystems* **3**, e00187–18 (2018).
28. B. Krawczyk, P. Wityk, M. Gałęcka, M. Michalik, The many faces of enterococcus spp.—Commensal, probiotic and opportunistic pathogen. *Microorganisms* **9**, 1900 (2021).
29. J. N. Weiser, D. M. Ferreira, J. C. Paton, Streptococcus pneumoniae: Transmission, colonization and invasion. *Nat. Rev. Microbiol.* **16**, 355–367 (2018).
30. A. Herbig, F. Maixner, K. I. Bos, A. Zink, J. Krause, D. H. Huson, MALT: Fast alignment and analysis of metagenomic DNA sequence data applied to the Tyrolean Iceman. bioRxiv 050559 [Preprint] (2016). https://doi.org/10.1101/050559.
31. M. Guellil, O. Kersten, A. Namouchi, E. L. Bauer, M. Derrick, A. Ø. Jensen, N. C. Stenseth, B. Bramanti, Genomic blueprint of a relapsing fever pathogen in 15th century Scandinavia. *Proc. Natl. Acad. Sci. U.S.A.* **115**, 10422–10427 (2018).
32. B. Mühlemann, L. Vinner, A. Margaryan, H. Wilhelmson, C. De La Fuente Castro, M. E. Allentoft, P. De Barros Damgaard, A. J. Hansen, S. Holtsmark Nielsen, L. M. Strand, J. Bill, A. Buzhilova, T. Pushkina, C. Falys, V. Khartanovich, V. Moiseyev, M. L. S. Jørkov, P. Østergaard Sørensen, Y. Magnusson, I. Gustin, H. Schroeder, G. Sutter, G. L. Smith, C. Drosten, R. A. M. Fouchier, D. J. Smith, E. Willerslev, T. C. Jones, M. Sikora, Diverse variola virus (smallpox) strains were widespread in northern Europe in the Viking Age. *Science* **369**, eaaw8977 (2020).
33. D. H. Alexander, J. Novembre, K. Lange, Fast model-based estimation of ancestry in unrelated individuals. *Genome Res.* **19**, 1655–1664 (2009).
34. J. M. R. Punzón, *El Mundo Funerario Rural En La Provincia de Granada Durante La Antigüedad Tardía* (Universidad de Granada, 2004).
35. J. L. Boone, Tribalism, ethnicity, and islamization in the Baixo Alentejo of Portugal: Preliminary results of investigation into transitional period (AD 550-850) rural settlements. *Era-Arqueol. Rev. Divulg. Cient. Estud. Arqueol.* **4**, 104–121 (2001).
36. T. Glick, "Islamic and Christian Spain in the Early Middle Ages: Second" in *Islamic and Christian Spain in the Early Middle Ages* (Brill, 2005).
37. H. Kennedy, *Muslim Spain and Portugal: A Political History of al-Andalus* (Routledge, 2014).
38. S. Inskip, Islam in Iberia or Iberian Islam: Bioarchaeology and the analysis of emerging Islamic identity in Early Medieval Iberia. *J. Post Classic. Archaeol.* **3**, 63–93 (2013).
39. N. Patterson, P. Moorjani, Y. Luo, S. Mallick, N. Rohland, Y. Zhan, T. Genschoreck, T. Webster, D. Reich, Ancient admixture in human history. *Genetics* **192**, 1065–1093 (2012).
40. R. Rodríguez-Varela, T. Günther, M. Krzewińska, J. Storå, T. H. Gillingwater, M. MacCallum, J. L. Arsuaga, K. Dobney, C. Valdiosera, M. Jakobsson, A. Götherström, L. Girdland-Flink, Genomic analyses of pre-european conquest human remains from the canary islands reveal close affinity to modern North Africans. *Curr. Biol.* **27**, 3396–3402.e5 (2017).
41. J. G. Serrano, The genomic history of the indigenous people of the Canary Islands. *Nat. Commun.* **14**, 4641 (2023).
42. L. R. Arauna, J. Mendoza-Revilla, A. Mas-Sandoval, H. Izaabel, A. Bekada, S. Benhamamouch, K. Fadhlaoui-Zid, P. Zalloua, G. Hellenthal, D. Comas, Recent historical migrations have shaped the gene pool of Arabs and Berbers in North Africa. *Mol. Biol. Evol.* **34**, 318–329 (2016).
43. E. Prevedorou, M. D.-Z. Bonilla, A. Romero, J. E. Buikstra, M. P. de Miguel Ibáñez, K. J. Knudson, Residential mobility and dental decoration in Early Medieval Spain: Results from the eighth century site of Plaza del Castillo, Pamplona. *Dent. Anthropol. J.* **23**, 42–52 (2010).
44. L. Jesús, E. P. de Garayo, "Al-Andalus?` en la periferia de Vasconia?: Sistemas de dominación de bilad Banbaluna y de Alaba wa-l-Qila en la octava centuria" in *Vasconia En La Alta Edad Media, 450-1000: Poderes y Comunidades Rurales En El Norte Peninsular* (Servicio de Publicaciones, 2011), pp. 55–70.
45. A. Flores-Bello, F. Bauduer, J. Salaberria, B. Oyharçabal, F. Calafell, J. Bertranpetit, L. Quintana-Murci, D. Comas, Genetic origins, singularity, and heterogeneity of Basques. *Curr. Biol.* **31**, 2167–2177.e4 (2021).
46. M. Romney, S. Cheung, V. Montessori, Erysipelothrix rhusiopathiae endocarditis and presumed osteomyelitis. *Can. J. Infect. Dis.* **12**, 912086 (1900).
47. M. Spiteri, A. Taylor-Robinson, *Erysipelothrix rhusiopathiae*: An important cause of bacterial disease in farmed pigs and an occupational pathogen of humans. *Int. J. Clin. Med. Microbiol.* **3**, 134 (2018).
48. T. L. Forde, K. Orsel, R. N. Zadoks, R. Biek, L. G. Adams, F. van der Meer, S. J. Kutz, Bacterial genomics reveal the complex epidemiology of an emerging pathogen in arctic and boreal ungulates. *Front. Microbiol.* **7**, 226383 (2016).
49. D. Chandler, J. Craven, Persistence and distribution of *Erysipelothrix rhusiopathiae* and bacterial indicator organisms on land used for disposal of piggery effluent. *J. Appl. Microbiol.* **48**, 367–375 (1980).
50. M. Sikora, E. Canteri, A. Fernandez-Guerra, N. Oskolkov, R. Agren, L. Hansson, E. K. Irving-Pease, B. Mühlemann, S. Holtsmark Nielsen, G. Scorrano, M. E. Allentoft, F. V. Seersholm, H. Schroeder, C. Gaunitz, J. Stenderup, L. Vinner, T. C. Jones, B. Nystedt, J. Parkhill, L. Fugger, F. Racimo, K. Kristiansen, A. K. N. Iversen, E. Willerslev, The landscape of ancient human pathogens in Eurasia from the Stone Age to historical times. bioRxiv 561165 [Preprint] (2023). https://doi.org/10.1101/2023.10.06.561165.

51. N. Bergfeldt, E. Kırdök, N. Oskolkov, C. Mirabello, P. Unneberg, H. Malmström, M. Fraser, F. Sanchez-Quinto, R. Jorgensen, B. Skar, Identification of microbial pathogens in Neolithic Scandinavian humans. *Sci. Rep.* **14**, 5630 (2024).
52. J. H. Bonczarowska, J. Susat, B. Mühlemann, I. Jasch-Boley, S. Brather, B. Höke, S. Brather-Walter, V. Schoenenberg, J. Scheschkewitz, G. Graenert, D. Krausse, M. Francken, T. C. Jones, J. Wahl, A. Nebel, B. Krause-Kyora, Pathogen genomics study of an early medieval community in Germany reveals extensive co-infections. *Genome Biol.* **23**, 250 (2022).
53. C. Thèves, E. Crubézy, P. Biagini, History of smallpox and its spread in human populations. *Microbiol. Spectr.* **4**, 4.4.05 (2016).
54. F. Fenner, D. Henderson, I. Arita, Z. Ježek, I. D. Ladnyi, *Smallpox and its Eradication* (World Health Organization, 1987), pp. 210.
55. E. C. Griffiths, A. B. Pedersen, A. Fenton, O. L. Petchey, The nature and consequences of coinfection in humans. *J. Infect.* **63**, 200–206 (2011).
56. J. Dabney, M. Knapp, I. Glocke, M.-T. Gansauge, A. Weihmann, B. Nickel, C. Valdiosera, N. Garcia, S. Paabo, J.-L. Arsuaga, M. Meyer, Complete mitochondrial genome sequence of a Middle Pleistocene cave bear reconstructed from ultrashort DNA fragments. *Proc. Natl. Acad. Sci. U.S.A.* **110**, 15758–15763 (2013).
57. M. Meyer, M. Kircher, Illumina sequencing library preparation for highly multiplexed target capture and sequencing. *Cold Spring Harb. Protoc.* **2010**, pdb.prot5448 (2010).
58. H. Li, R. Durbin, Fast and accurate short read alignment with Burrows–Wheeler transform. *Bioinformatics* **25**, 1754–1760 (2009).
59. H. Li, B. Handsaker, A. Wysoker, T. Fennell, J. Ruan, N. Homer, G. Marth, G. Abecasis, R. Durbin, The sequence alignment/map format and SAMtools. *Bioinformatics* **25**, 2078–2079 (2009).
60. M. Kircher, "Analysis of high-throughput ancient DNA sequencing data" in *Ancient DNA*, B. Shapiro, M. Hofreiter, Eds. (Humana Press, 2012), vol. 840, pp. 197–228.
61. P. Skoglund, H. Malmstrom, M. Raghavan, J. Stora, P. Hall, E. Willerslev, M. T. P. Gilbert, A. Gotherstrom, M. Jakobsson, Origins and genetic legacy of neolithic farmers and hunter-gatherers in Europe. *Science* **336**, 466–469 (2012).
62. P. Skoglund, B. H. Northoff, M. V. Shunkov, A. P. Derevianko, S. Pääbo, J. Krause, M. Jakobsson, Separating endogenous ancient DNA from modern day contamination in a Siberian Neandertal. *Proc. Natl. Acad. Sci. U.S.A.* **111**, 2229–2234 (2014).
63. R. E. Green, A.-S. Malaspinas, J. Krause, A. W. Briggs, P. L. F. Johnson, C. Uhler, M. Meyer, J. M. Good, T. Maricic, U. Stenzel, K. Prüfer, M. Siebauer, H. A. Burbano, M. Ronan, J. M. Rothberg, M. Egholm, P. Rudan, D. Brajković, Ž. Kućan, I. Gušić, M. Wikström, L. Laakkonen, J. Kelso, M. Slatkin, S. Pääbo, A complete neandertal mitochondrial genome sequence determined by high-throughput sequencing. *Cell* **134**, 416–426 (2008).
64. Q. Fu, A. Mittnik, P. L. F. Johnson, K. Bos, M. Lari, R. Bollongino, C. Sun, L. Giemsch, R. Schmitz, J. Burger, A. M. Ronchitelli, F. Martini, R. G. Cremonesi, J. Svoboda, P. Bauer, D. Caramelli, S. Castellano, D. Reich, S. Pääbo, J. Krause, A revised timescale for human evolution based on ancient mitochondrial genomes. *Curr. Biol.* **23**, 553–559 (2013).
65. T. S. Korneliussen, A. Albrechtsen, R. Nielsen, ANGSD: Analysis of next generation sequencing data. *BMC Bioinformatics* **15**, 356 (2014).
66. M. Rasmussen, X. Guo, Y. Wang, K. E. Lohmueller, S. Rasmussen, A. Albrechtsen, L. Skotte, S. Lindgreen, M. Metspalu, T. Jombart, T. Kivisild, W. Zhai, A. Eriksson, A. Manica, L. Orlando, F. M. De La Vega, S. Tridico, E. Metspalu, K. Nielsen, M. C. Avila-Arcos, J. V. Moreno-Mayar, C. Muller, J. Dortch, M. T. P. Gilbert, O. Lund, A. Wesolowska, M. Karmin, L. A. Weinert, B. Wang, J. Li, S. Tai, F. Xiao, T. Hanihara, G. van Driem, A. R. Jha, F.-X. Ricaut, P. de Knijff, A. B. Migliano, I. Gallego Romero, K. Kristiansen, D. M. Lambert, S. Brunak, P. Forster, B. Brinkmann, O. Nehlich, M. Bunce, M. Richards, R. Gupta, C. D. Bustamante, A. Krogh, R. A. Foley, M. M. Lahr, F. Balloux, T. Sicheritz-Ponten, R. Villems, R. Nielsen, J. Wang, E. Willerslev, An aboriginal australian genome reveals separate human dispersals into Asia. *Science* **334**, 94–98 (2011).
67. L. C. Reimer, J. Sardà Carbasse, J. Koblitz, C. Ebeling, A. Podstawka, J. Overmann, BacDive in 2022: The knowledge base for standardized bacterial and archaeal data. *Nucleic Acids Res.* **50**, D741–D746 (2022).
68. Microbe Atlas Project Database; https://microbeatlas.org.
69. T. Sasi Jyothsna, L. Tushar, C. Sasikala, C. V. Ramana, *Paraclostridium benzoelyticum* gen. nov., sp. nov., isolated from marine sediment and reclassification of *Clostridium bifermentans* as *Paraclostridium bifermentans* comb. nov. Proposal of a new genus *Paeniclostridium* gen. nov. to accommodate *Clostridium sordellii* and *Clostridium ghonii*. *Int. J. Syst. Evol. Microbiol.* **66**, 1268–1274 (2016).
70. V. G. Krishnaswamy, R. Sridharan, P. S. Kumar, M. J. Fathima, Cellulase enzyme catalyst producing bacterial strains from vermicompost and its application in low-density polyethylene degradation. *Chemosphere* **288**, 132552 (2022).
71. H. Thorvaldsdottir, J. T. Robinson, J. P. Mesirov, Integrative Genomics Viewer (IGV): High-performance genomics data visualization and exploration. *Brief. Bioinform.* **14**, 178–192 (2013).
72. M. Guellil, MeriamGuellil/aDNA-BAMPlotter:, version v2.0.1. *Zenodo* (2021); https://doi.org/10.5281/zenodo.5702679.
73. H. Jónsson, A. Ginolhac, M. Schubert, P. L. F. Johnson, L. Orlando, mapDamage2.0: Fast approximate Bayesian estimates of ancient DNA damage parameters. *Bioinformatics* **29**, 1682–1684 (2013).
74. R. Hübler, F. M. Key, C. Warinner, K. I. Bos, J. Krause, A. Herbig, HOPS: Automated detection and authentication of pathogen DNA in archaeological remains. *Genome Biol.* **20**, 280 (2019).
75. B. Langmead, S. L. Salzberg, Fast gapped-read alignment with Bowtie 2. *Nat. Methods* **9**, 357–359 (2012).
76. A. T. Duggan, M. F. Perdomo, D. Piombino-Mascali, S. Marciniak, D. Poinar, M. V. Emery, J. P. Buchmann, S. Duchêne, R. Jankauskas, M. Humphreys, G. B. Golding, J. Southon, A. Devault, J.-M. Rouillard, J. W. Sahl, O. Dutour, K. Hedman, A. Sajantila, G. L. Smith, E. C. Holmes, H. N. Poinar, 17th century variola virus reveals the recent history of smallpox. *Curr. Biol.* **26**, 3407–3412 (2016).
77. G. Ferrari, J. Neukamm, H. T. Baalsrud, A. M. Breidenstein, M. Ravinet, C. Phillips, F. Rühli, A. Bouwman, V. J. Schuenemann, Variola virus genome sequenced from an eighteenth-century museum specimen supports the recent origin of smallpox. *Philos. Trans. R. Soc. B* **375**, 20190572 (2020).
78. S. Chen, Y. Zhou, Y. Chen, J. Gu, fastp: An ultra-fast all-in-one FASTQ preprocessor. *Bioinformatics* **34**, i884–i890 (2018).
79. T. Magoč, S. L. Salzberg, FLASH: Fast length adjustment of short reads to improve genome assemblies. *Bioinformatics* **27**, 2957–2963 (2011).
80. S. Andrews, FastQC: A quality control tool for high throughput sequence data. BibSonomy. (2010); www.bioinformatics.babraham.ac.uk/projects/fastqc/ [accessed 17 March 2022]
81. P. Danecek, J. K. Bonfield, J. Liddle, J. Marshall, V. Ohan, M. O. Pollard, A. Whitwham, T. Keane, S. A. McCarthy, R. M. Davies, H. Li, Twelve years of SAMtools and BCFtools. *Gigascience* **10**, giab008 (2021).
82. G. Jun, M. K. Wing, G. R. Abecasis, H. M. Kang, An efficient and scalable analysis framework for variant extraction and refinement from population-scale DNA sequence data. *Genome Res.* **25**, 918–925 (2015).
83. B. Q. Minh, H. A. Schmidt, O. Chernomor, D. Schrempf, M. D. Woodhams, A. von Haeseler, R. Lanfear, IQ-TREE 2: New models and efficient methods for phylogenetic inference in the genomic era. *Mol. Biol. Evol.* **37**, 1530–1534 (2020).
84. S. Kalyaanamoorthy, B. Q. Minh, T. K. Wong, A. Von Haeseler, L. S. Jermiin, ModelFinder: Fast model selection for accurate phylogenetic estimates. *Nat. Methods* **14**, 587–589 (2017).
85. I. Minkin, P. Medvedev, Scalable multiple whole-genome alignment and locally collinear block construction with SibeliaZ. *Nat. Commun.* **11**, 6327 (2020).
86. A. Kloss-Brandstätter, D. Pacher, S. Schönherr, H. Weissensteiner, R. Binna, G. Specht, F. Kronenberg, HaploGrep: A fast and reliable algorithm for automatic classification of mitochondrial DNA haplogroups. *Hum. Mutat.* **32**, 25–32 (2011).
87. H. Weissensteiner, D. Pacher, A. Kloss-Brandstätter, L. Forer, G. Specht, H.-J. Bandelt, F. Kronenberg, A. Salas, S. Schönherr, HaploGrep 2: Mitochondrial haplogroup classification in the era of high-throughput sequencing. *Nucleic Acids Res.* **44**, W58–W63 (2016).
88. M. van Oven, M. Kayser, Updated comprehensive phylogenetic tree of global human mitochondrial DNA variation. *Hum. Mutat.* **30**, E386–E394 (2009).
89. R. Martiniano, B. D. Sanctis, P. Hallast, R. Durbin, Placing ancient DNA sequences into reference phylogenies. *Mol. Biol. Evol.* **39**, msac017 (2022).
90. M. Lipatov, K. Sanjeev, R. Patro, K. Veeramah, Maximum likelihood estimation of biological relatedness from low coverage sequencing data. bioRxiv 023374 [Preprint] (2015). https://doi.org/10.1101/023374.
91. D. Popli, S. Peyrégne, B. M. Peter, KIN: A method to infer relatedness from low-coverage ancient DNA. *Genome Biol.* **24**, 10 (2023).
92. K. Hanghøj, I. Moltke, P. A. Andersen, A. Manica, T. S. Korneliussen, Fast and accurate relatedness estimation from high-throughput sequencing data in the presence of inbreeding. *Gigascience* **8**, giz034 (2019).
93. A. L. Severson, T. S. Korneliussen, I. Moltke, LocalNgsRelate: A software tool for inferring IBD sharing along the genome between pairs of individuals from low-depth NGS data. *Bioinformatics* **38**, 1159–1161 (2022).
94. L. Pagani, D. J. Lawson, E. Jagoda, A. Mörseburg, A. Eriksson, M. Mitt, F. Clemente, G. Hudjashov, M. DeGiorgio, L. Saag, J. D. Wall, A. Cardona, R. Mägi, M. A. W. Sayres, S. Kaewert, C. Inchley, C. L. Scheib, M. Järve, M. Karmin, G. S. Jacobs, T. Antao, F. M. Iliescu, A. Kushniarevich, Q. Ayub, C. Tyler-Smith, Y. Xue, B. Yunusbayev, K. Tambets, C. B. Mallick, L. Saag, E. Pocheshkhova, G. Andriadze, C. Muller, M. C. Westaway, D. M. Lambert, G. Zoraqi, S. Turdikulova, D. Dalimova, Z. Sabitov, G. N. N. Sultana, J. Lachance, S. Tishkoff, K. Momynaliev, J. Isakova, L. D. Damba, M. Gubina, P. Nymadawa, I. Evseeva, L. Atramentova, O. Utevska, F.-X. Ricaut, N. Brucato, H. Sudoyo, T. Letellier, M. P. Cox, N. A. Barashkov, V. Škaro, L. Mulahasanović, D. Primorac, H. Sahakyan, M. Mormina, C. A. Eichstaedt, D. V. Lichman, S. Abdullah, G. Chaubey, J. T. S. Wee, E. Mihailov, A. Karunas, S. Litvinov, R. Khusainova, N. Ekomasova, V. Akhmetova, I. Khidiyatova, D. Marjanović, L. Yepiskoposyan, D. M. Behar, E. Balanovska, A. Metspalu, M. Derenko,

B. Malyarchuk, M. Voevoda, S. A. Fedorova, L. P. Osipova, M. M. Lahr, P. Gerbault, M. Leavesley, A. B. Migliano, M. Petraglia, O. Balanovsky, E. K. Khusnutdinova, E. Metspalu, M. G. Thomas, A. Manica, R. Nielsen, R. Villems, E. Willerslev, T. Kivisild, M. Metspalu, Genomic analyses inform on migration events during the peopling of Eurasia. *Nature* **538**, 238–242 (2016).

95. 1000 Genomes Project Consortium, A global reference for human genetic variation. *Nature* **526**, 68–74 (2015).
96. R. Yaka, I. Mapelli, D. Kaptan, A. Doğu, M. Chyleński, Ö. D. Erdal, D. Koptekin, K. B. Vural, A. Bayliss, C. Mazzucato, E. Fer, S. S. Çokoğlu, V. K. Lagerholm, M. Krzewińska, C. Karamurat, H. C. Gemici, A. Sevkar, N. D. Dağtaş, G. M. Kılınç, D. Adams, A. R. Munters, E. Sağlıcan, M. Milella, E. M. J. Schotsmans, E. Yurtman, M. Çetin, S. Yorulmaz, N. E. Altınışık, A. Ghalichi, A. Juras, C. C. Bilgin, T. Günther, J. Storå, M. Jakobsson, M. de Kleijn, G. Mustafaoğlu, A. Fairbairn, J. Pearson, İ. Togan, N. Kayacan, A. Marciniak, C. S. Larsen, I. Hodder, Ç. Atakuman, M. Pilloud, E. Sürer, F. Gerritsen, R. Özbal, D. Baird, Y. S. Erdal, G. Duru, M. Özbaşaran, S. D. Haddow, C. J. Knüsel, A. Götherström, F. Özer, M. Somel, Variable kinship patterns in Neolithic Anatolia revealed by ancient genomes. *Curr. Biol.* **31**, 2455–2468.e18 (2021).
97. N. Patterson, A. L. Price, D. Reich, Population structure and eigenanalysis. *PLOS Genet.* **2**, e190 (2006).
98. S. Purcell, B. Neale, K. Todd-Brown, L. Thomas, M. A. R. Ferreira, D. Bender, J. Maller, P. Sklar, P. I. W. de Bakker, M. J. Daly, P. C. Sham, PLINK: A tool set for whole-genome association and population-based linkage analyses. *Am. J. Hum. Genet.* **81**, 559–575 (2007).
99. A. A. Behr, K. Z. Liu, G. Liu-Fang, P. Nakka, S. Ramachandran, Pong: Fast analysis and visualization of latent clusters in population genetic data. *Bioinformatics* **32**, 2817–2823 (2016).
100. É. Harney, N. Patterson, D. Reich, J. Wakeley, Assessing the performance of qpAdm: A statistical tool for studying population admixture. *Genetics* **217**, iyaa045 (2021).
101. I. Lazaridis, D. Nadel, G. Rollefson, D. C. Merrett, N. Rohland, S. Mallick, D. Fernandes, M. Novak, B. Gamarra, K. Sirak, S. Connell, K. Stewardson, E. Harney, Q. Fu, G. Gonzalez-Fortes, E. R. Jones, S. A. Roodenberg, G. Lengyel, F. Bocquentin, B. Gasparian, J. M. Monge, M. Gregg, V. Eshed, A.-S. Mizrahi, C. Meiklejohn, F. Gerritsen, L. Bejenaru, M. Blüher, A. Campbell, G. Cavalleri, D. Comas, P. Froguel, E. Gilbert, S. M. Kerr, P. Kovacs, J. Krause, D. McGettigan, M. Merrigan, D. A. Merriwether, S. O'Reilly, M. B. Richards, O. Semino, M. Shamoon-Pour, G. Stefanescu, M. Stumvoll, A. Tönjes, A. Torroni, J. F. Wilson, L. Yengo, N. A. Hovhannisyan, N. Patterson, R. Pinhasi, D. Reich, Genomic insights into the origin of farming in the ancient Near East. *Nature* **536**, 419–424 (2016).
102. N. Patterson, M. Isakov, T. Booth, L. Büster, C.-E. Fischer, I. Olalde, H. Ringbauer, A. Akbari, O. Cheronet, M. Bleasdale, N. Adamski, E. Altena, R. Bernardos, S. Brace, N. Broomandkhoshbacht, K. Callan, F. Candilio, B. Culleton, E. Curtis, L. Demetz, K. S. D. Carlson, C. J. Edwards, D. M. Fernandes, M. G. B. Foody, S. Freilich, H. Goodchild, A. Kearns, A. M. Lawson, I. Lazaridis, M. Mah, S. Mallick, K. Mandl, A. Micco, M. Michel, G. B. Morante, J. Oppenheimer, K. T. Özdoğan, L. Qiu, C. Schattke, K. Stewardson, J. N. Workman, F. Zalzala, Z. Zhang, B. Agustí, T. Allen, K. Almássy, L. Amkreutz, A. Ash, C. Baillif-Ducros, A. Barclay, L. Bartosiewicz, K. Baxter, Z. Bernert, J. Blažek, M. Bodružić, P. Boissinot, C. Bonsall, P. Bradley, M. Brittain, A. Brookes, F. Brown, L. Brown, R. Brunning, C. Budd, J. Burmaz, S. Canet, S. Carnicero-Cáceres, M. Čaušević-Bully, A. Chamberlain, S. Chauvin, S. Clough, N. Čondić, A. Coppa, O. Craig, M. Črešnar, V. Cummings, S. Czifra, A. Danielisová, R. Daniels, A. Davies, P. de Jersey, J. Deacon, C. Deminger, P. W. Ditchfield, M. Dizdar, M. Dobeš, M. Dobisíková, L. Domboróczki, G. Drinkall, A. Đukić, M. Ernée, C. Evans, J. Evans, M. Fernández-Götz, S. Filipović, A. Fitzpatrick, H. Fokkens, C. Fowler, A. Fox, Z. Gallina, M. Gamble, M. R. G. Morales, B. González-Rabanal, A. Green, K. Gyenesei, D. Habermehl, T. Hajdu, D. Hamilton, J. Harris, C. Hayden, J. Hendriks, B. Hernu, G. Hey, M. Horňák, G. Ilon, E. Istvánovits, A. M. Jones, M. B. Kavur, K. Kazek, R. A. Kenyon, A. Khreisheh, V. Kiss, J. Kleijne, M. Knight, L. M. Kootker, P. F. Kovács, A. Kozubová, G. Kulcsár, V. Kulcsár, C. Le Pennec, M. Legge, M. Leivers, L. Loe, O. López-Costas, T. Lord, D. Los, J. Lyall, A. B. Marín-Arroyo, P. Mason, D. Matošević, A. Maxted, L. McIntyre, J. McKinley, K. McSweeney, B. Meijlink, B. G. Mende, M. Menđušić, M. Metlička, S. Meyer, K. Mihovilić, L. Milasinovic, S. Minnitt, J. Moore, G. Morley, G. Mullan, M. Musilová, B. Neil, R. Nicholls, M. Novak, M. Pala, M. Papworth, C. Paresys, R. Patten, D. Perkić, K. Pesti, A. Petit, K. Petriščáková, C. Pichon, C. Pickard, Z. Pilling, T. D. Price, S. Radović, R. Redfern, B. Resutík, D. T. Rhodes, M. B. Richards, A. Roberts, J. Roefstra, P. Sankot, A. Šefčáková, A. Sheridan, S. Skae, M. Šmolíková, K. Somogyi, Á. Somogyvári, M. Stephens, G. Szabó, A. Szécsényi-Nagy, T. Szeniczey, J. Tabor, K. Tankó, C. T. Maria, R. Terry, B. Teržan, M. Teschler-Nicola, J. F. Torres-Martínez, J. Trapp, R. Turle, F. Ujvári, M. van der Heiden, P. Veleminsky, B. Veselka, Z. Vytlačil, C. Waddington, P. Ware, P. Wilkinson, L. Wilson, R. Wiseman, E. Young, J. Zaninović, A. Žitňan, C. Lalueza-Fox, P. de Knijff, I. Barnes, P. Halkon, M. G. Thomas, D. J. Kennett, B. Cunliffe, M. Lillie, N. Rohland, R. Pinhasi, I. Armit, D. Reich, Large-scale migration into Britain during the Middle to Late Bronze Age. *Nature* **601**, 588–594 (2022).
103. J. M. Monsalvo Antón, *Atlas Histórico de la España Medieval* (Síntesis, 2010).
104. S. Brunel, E. A. Bennett, L. Cardin, D. Garraud, H. Barrand Emam, A. Beylier, B. Boulestin, F. Chenal, E. Ciesielski, F. Convertini, B. Dedet, S. Desbrosse-Degobertiere, S. Desenne, J. Dubouloz, H. Duday, G. Escalon, V. Fabre, E. Gailledrat, M. Gandelin, Y. Gleize, S. Goepfert, J. Guilaine, L. Hachem, M. Ilett, F. Lambach, F. Maziere, B. Perrin, S. Plouin, E. Pinard, I. Praud, I. Richard, V. Riquier, R. Roure, B. Sendra, C. Thevenet, S. Thiol, E. Vauquelin, L. Vergnaud, T. Grange, E.-M. Geigl, M. Pruvost, Ancient genomes from present-day France unveil 7,000 years of its demographic history. *Proc. Natl. Acad. Sci. U.S.A.* **117**, 12791–12798 (2020).

Acknowledgments: We acknowledge support from the National Genomics Infrastructure in Stockholm funded by Science for Life Laboratory, the Knut and Alice Wallenberg Foundation, and the Swedish Research Council, and the computations were enabled by resources in project (NAISS 2023/23-75, NAISS 2023-22-156, NAISS 2023/5-252, NAISS 2023/22-612, NAISS 2023/6-36, and NAISS 2022/6-144) provided by the National Academic Infrastructure for Supercomputing in Sweden (NAISS) at UPPMAX, funded by the Swedish Research Council through grant agreement no. 2022-06725. The archaeological excavation has been funded by the Research Project HAR2010-20903 "Cave habitat in the north of the Iberian Peninsula. Systematic analysis of the complex of Las Gobas, (Condado de Treviño, Burgos)" granted to the GPAC Group of the University of the Basque Country by the Ministry of Economy and Competitiveness and Research of the Government of Spain. We would like to thank P. Swali, A. Sevkar, and E. Kırdök for comments and suggestions. **Funding:** This work was supported by Swedish Research Council project ID 2019-00849_VR and ATLAS (Riksbankens Jubileumsfond). **Author contributions:** Conceptualization: R.R.-V., A.G., A.A., I.S.-P., L.H., and J.L.S. Methodology: R.R.-V., R.Y., Z.P., B.G., M.K., T.N., V.A.P., and V.K.L. Investigation: R.R.-V., A.G., A.A., I.S.-P., L.H., M.K., R.Y., Z.P., B.G., J.L.S., and P.P.-.R. Metagenomics: Z.P., B.G., R.R.-V., and A.G. Visualization: R.R.-V., Z.P., B.G., I.S.-P., and M.K. Supervision: R.R.-V., A.G., A.A., L.H., I.S.-P., and J.L.S. Writing—original draft: R.R.-V. Writing—review and editing: R.R.-V., A.G., R.Y., I.S.-P., J.L.S., Z.P., B.G., T.N., P.P.-.R., V.K.L., C.V., M.K., L.H., and A.A. **Competing interests:** The authors declare that they have no competing interests. **Data and materials availability:** All data needed to evaluate the conclusions in the paper are present in the paper and/or the Supplementary Materials. Fastq files are available at the European Nucleotide Archive (ENA) with accession number PRJEB72246.

Submitted 15 April 2024
Accepted 19 July 2024
Published 28 August 2024
10.1126/sciadv.adp8625
