# Supplementary material for: Five centuries of consanguinity, isolation, health, and conflict in Las Gobas: A Northern Medieval Iberian necropolis
Source: Sci Adv. 2024 Aug 28;10(35):eadp8625. doi: 10.1126/sciadv.adp8625 (PMC11352919; doi:10.1126/sciadv.adp8625)
Supplement: Supplementary file 2 — Figs. S1 to S24 Legends for tables S1 to S12 References [file sciadv.adp8625_sm.v2.pdf]

CORRECTED 31 OCTOBER 2025; SEE BELOW

Science Advances
AAAS

Supplementary Materials for

Five centuries of consanguinity, isolation, health, and conflict in Las Gobas: A Northern Medieval Iberian necropolis

Ricardo Rodríguez-Varela *et al.*

Corresponding author: Ricardo Rodríguez-Varela, ricardo.rodriguez.varela@arklab.su.se;
Anders Götherström, anders.gotherstrom@arklab.su.se

Sci. Adv. **10**, eadp8625 (2024)
DOI: 10.1126/sciadv.adp8625

The PDF file includes:

Figs. S1 to S24
Legends for tables S1 to S12
References

Other Supplementary Material for this manuscript includes the following:

Tables S1 to S12

Correction (31 October 2025): In the original table S2 file, column M was incorrectly labeled "gender." In the revised version, column M is now labeled "genetic sex," consistent with the designation used in table S1. The authors' results and conclusions are not affected by this change. The supplementary tables file has been replaced with the corrected version of table S2.

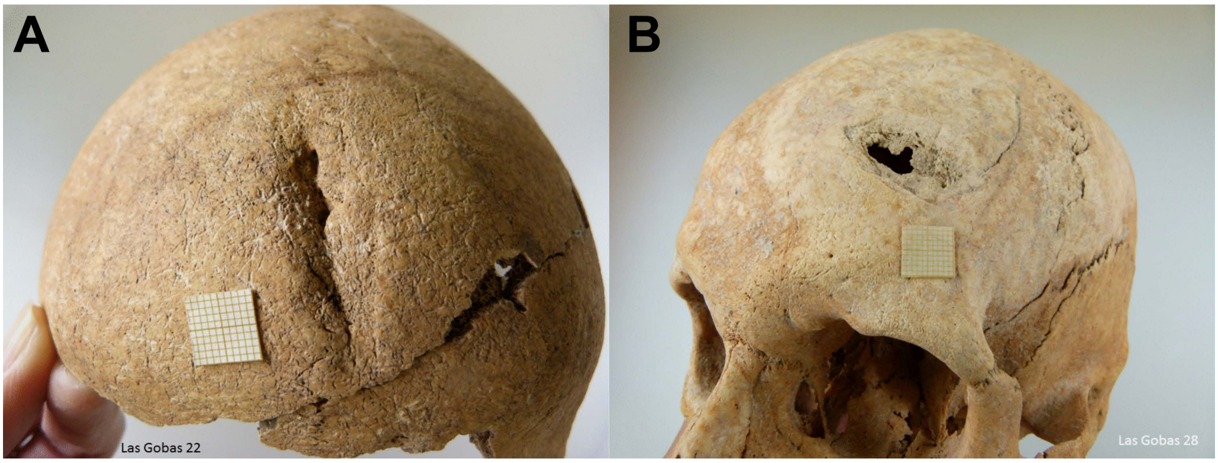

Fig. S1. **Violence signs.** (**A**) Individual 22 was struck with a sharp weapon, presumably a sword judging from the length of the injury. The attacker was facing the victim and inflicted the incision from his left, which explains its diagonal disposition. Despite the notable injury, this individual survived. (**B**) Individual 28. Injury of an incised-contuse type, caused by a sharp-bladed weapon that struck the posterior part of the frontal bone. The individual was struck on their left side by a posterior-anterior motion. He died from the deep wound (Photos: L. Herrasti, F. Etxeberria).

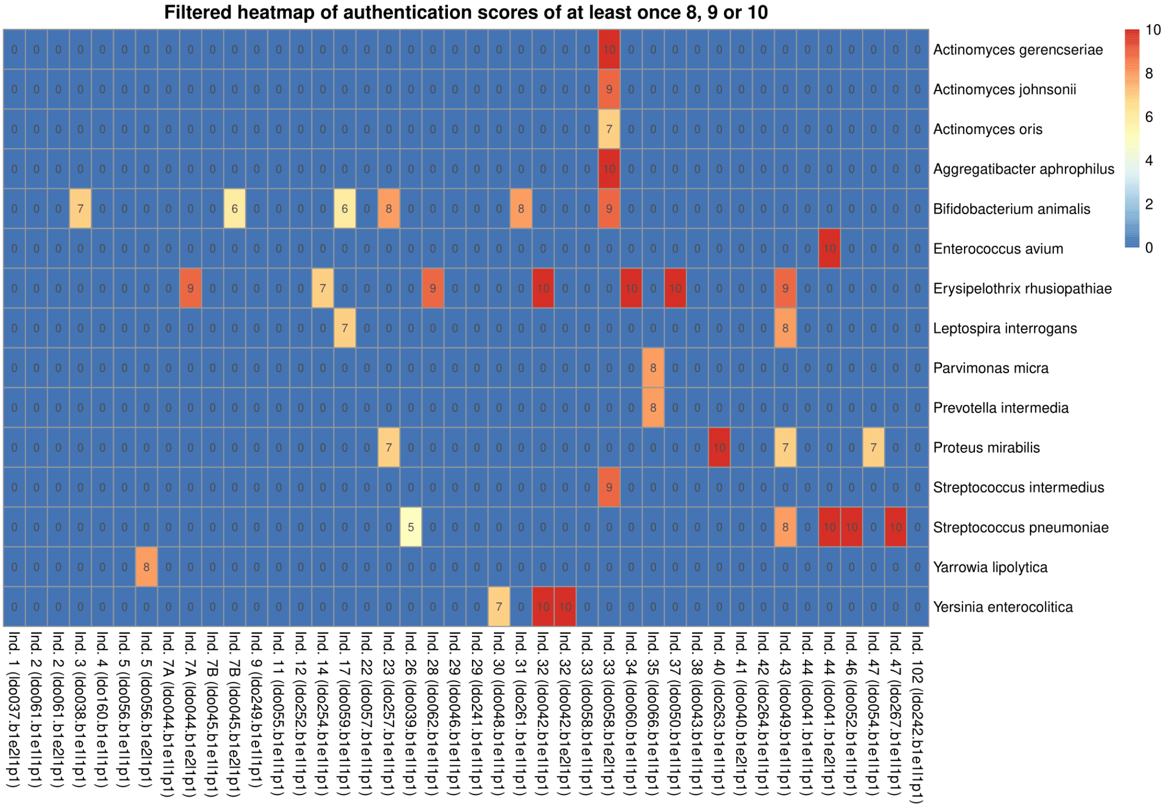

Fig. S2. Heatmap of the authentication scores per microbe and individual. Authentication scores are based on 9 metrics (edit distance, edit distance of ancient reads, deamination on both read ends, mean read length, PMD score, ANI, amount of reads and evenness of coverage, the latter adding 2 points) and range from 1 to 10.

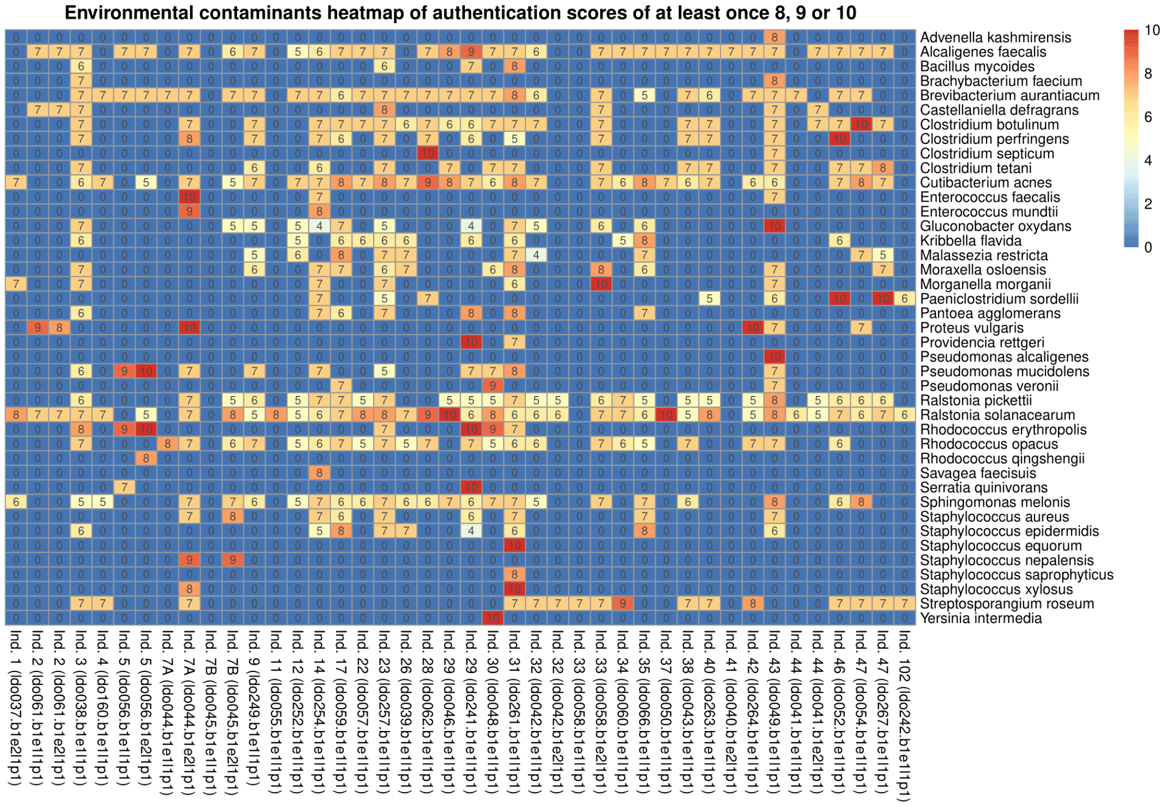

Fig. S3. Heatmap of the authentication scores per microbe and individual for likely environmental contaminants. Authentication scores are based on 9 metrics (edit distance, edit distance of ancient reads, deamination on both read ends, mean read length, PMD score, ANI, amount of reads and evenness of coverage, the latter adding 2 points) and range from 1 to 10.

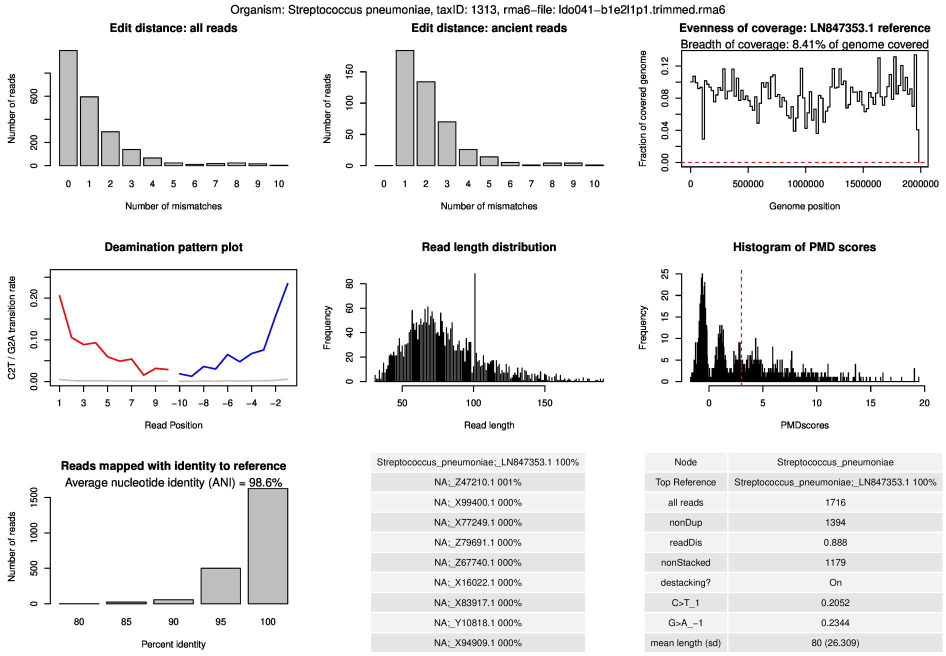

Fig. S4. Authentication plots for *Streptococcus pneumoniae*. Individual 44 (library: ldo041-b1e2l1p1) representing edit distance for all reads and ancient reads, breadth of coverage, deamination pattern, read length distribution, PMD scores, percent identity, table of top mapping reference and table of statistics. Authentication score = 10.

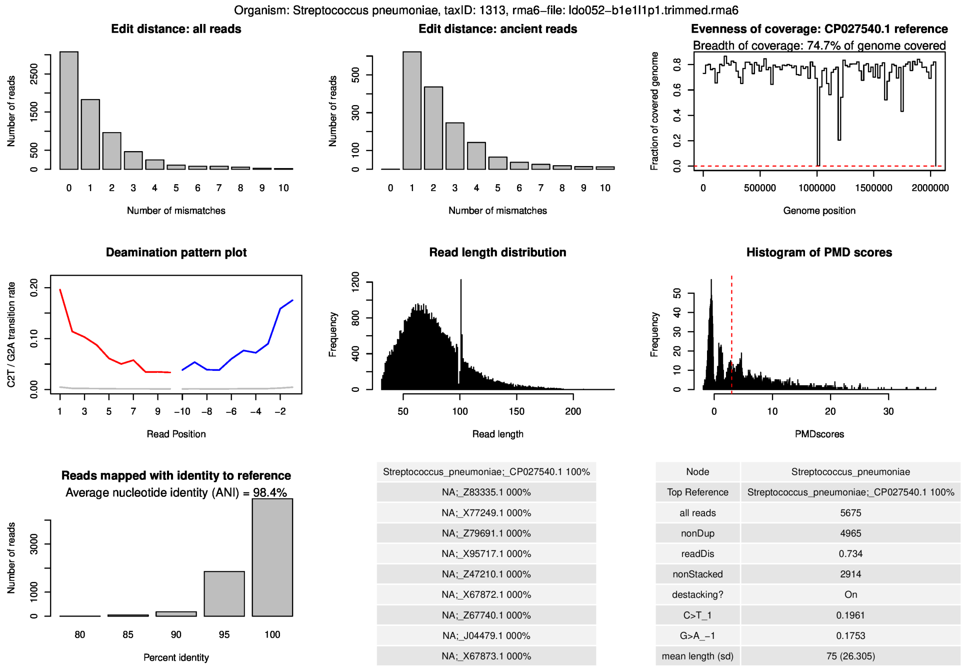

Fig. S5. Authentication plots for *Streptococcus pneumoniae*. Individual 46 (library: ldo052-b1e1l1p1) representing edit distance for all reads and ancient reads, breadth of coverage, deamination pattern, read

length distribution, PMD scores, percent identity, table of top mapping reference and table of statistics. Authentication score = 10.

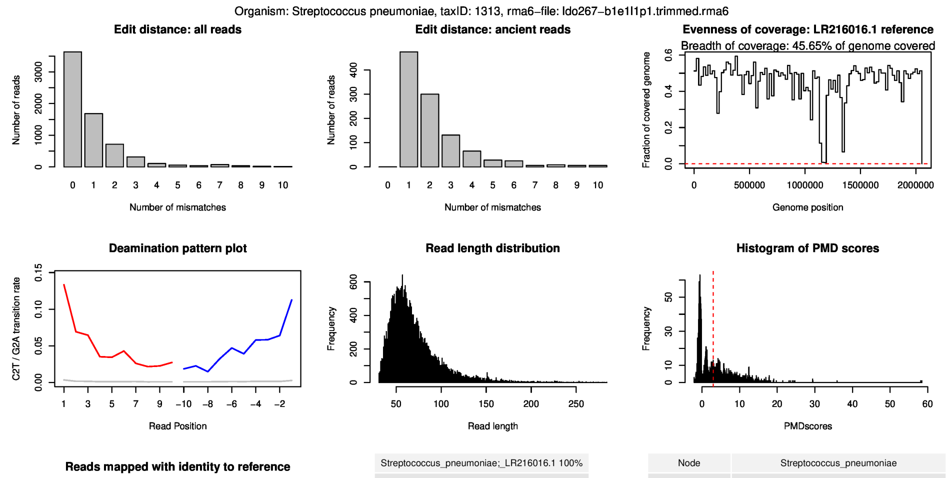

Streptococcus_pneumoniae;_LR216016.1 100%
NA;_X77249.1 000%
NA;_Z83335.1 000%
NA;_X95717.1 000%
NA;_Z79691.1 000%
NA;_Z82001.1 000%
NA;_Z47210.1 000%
NA;_X85787.1 000%
NA;_Z67740.1 000%
NA;_X67873.1 000%

Node	Streptococcus_pneumoniae
Top Reference	Streptococcus_pneumoniae;_LR216016.1 100%
all reads	5916
nonDup	4864
readDis	0.77
nonStacked	3038
destacking?	On
C>T_1	0.1333
G>A_-1	0.1127
mean length (sd)	69 (25.993)

Fig. S6. Authentication plots for *Streptococcus pneumoniae*. Individual 47 (library: ldo267-b1e1l1p1) representing edit distance for all reads and ancient reads, breadth of coverage, deamination pattern, read length distribution, PMD scores, percent identity, table of top mapping reference and table of statistics. Authentication score = 10.

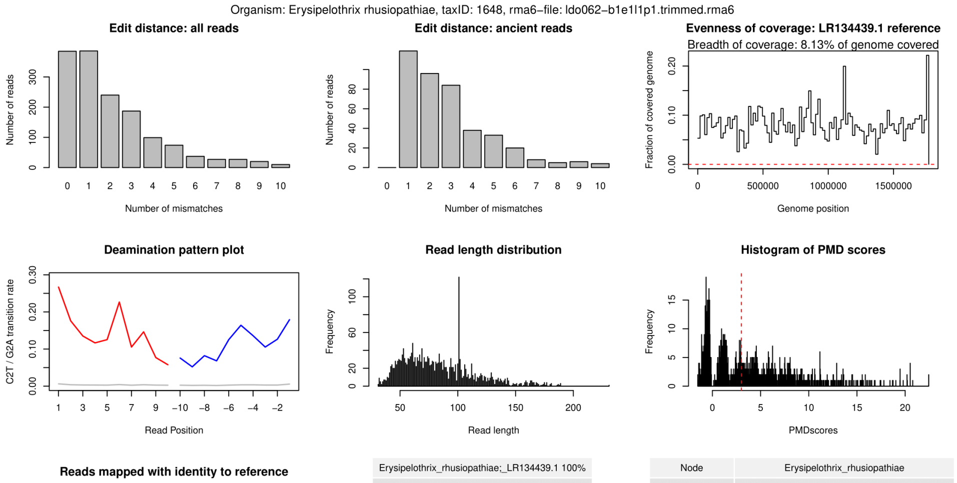

Erysipelothrix_rhusiopathiae;_LR134439.1 100%
NA;_M23728.1 000%
NA;_MH151264.1 000%
NA;_MH151296.1 000%
NA;_MN599993.1 000%
NA;_MH151227.1 000%
NA;_MF568073.1 000%
NA;_MF568072.1 000%
NA%
NA%

Node	Erysipelothrix_rhusiopathiae
Top Reference	Erysipelothrix_rhusiopathiae;_LR134439.1 100%
all reads	1878
nonDup	1586
readDis	0.889
nonStacked	1307
destacking?	On
C>T_1	0.2671
G>A_-1	0.1792
mean length (sd)	83 (30.702)

Fig. S7. Authentication plots for *Erysipelothrix rhusiopathiae*. Individual 28 (library: ldo062-b1e1l1p1) representing edit distance for all reads and ancient reads, breadth of coverage, deamination pattern, read length distribution, PMD scores, percent identity, table of top mapping reference and table of statistics. Authentication score = 9.

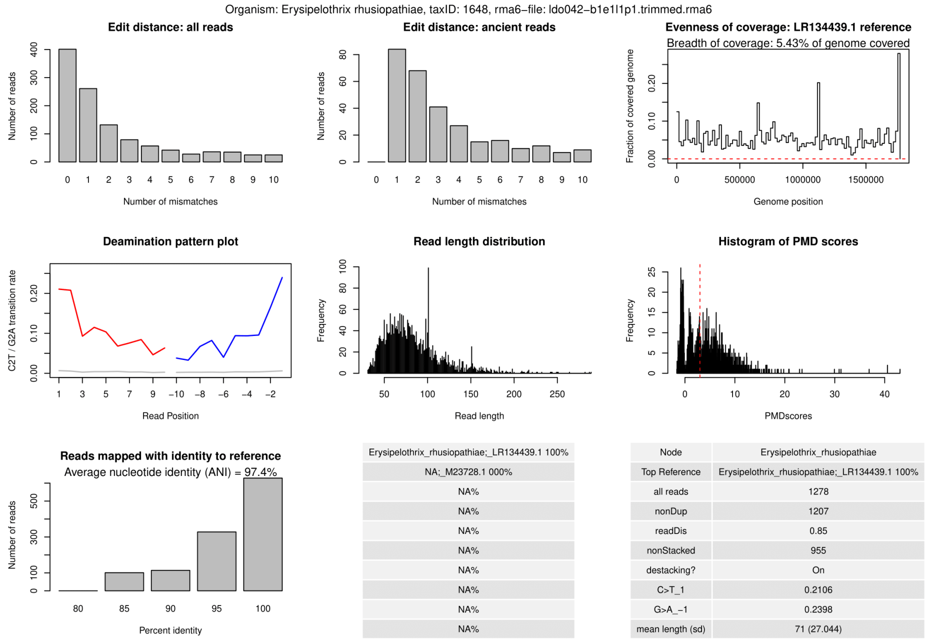

Erysipelothrix_rhusiopathiae;_LR134439.1 100%
NA;_M23728.1 000%
NA%
NA%
NA%
NA%
NA%
NA%
NA%
NA%

Node	Erysipelothrix_rhusiopathiae
Top Reference	Erysipelothrix_rhusiopathiae;_LR134439.1 100%
all reads	1278
nonDup	1207
readDis	0.85
nonStacked	955
destacking?	On
C>T_1	0.2106
G>A_−1	0.2398
mean length (sd)	71 (27.044)

Fig. S8. Authentication plots for *Erysipelothrix rhusiopathiae*. Individual 32 (library: ldo042-b1e1l1p1) representing edit distance for all reads and ancient reads, breadth of coverage, deamination pattern, read length distribution, PMD scores, percent identity, table of top mapping reference and table of statistics. Authentication score = 10.

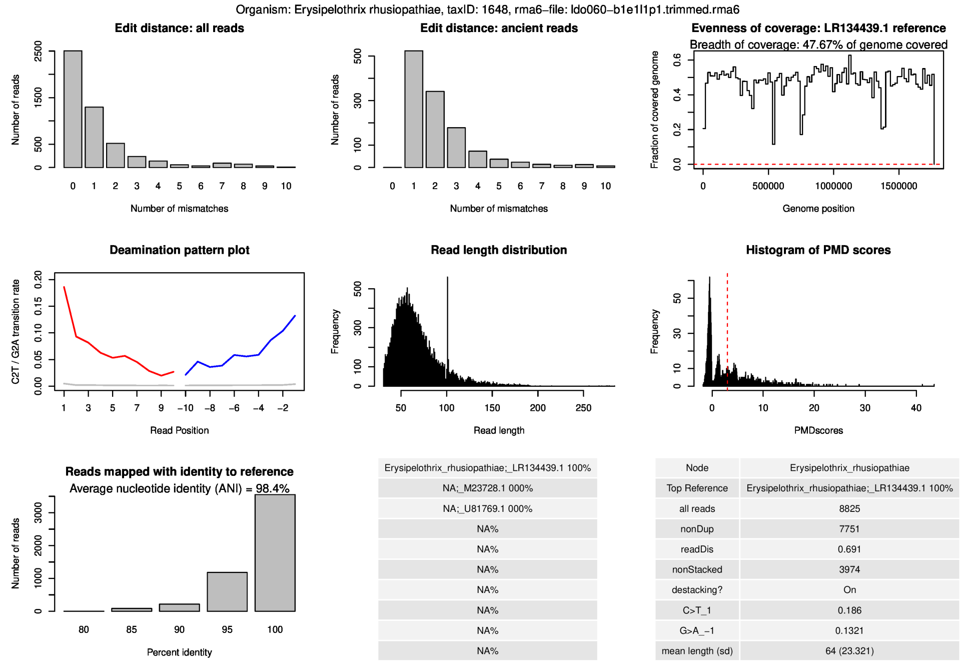

Fig. S9. Authentication plots for *Erysipelothrix rhusiopathiae*. Individual 34 (library: ldo060-b1e1l1p1) representing edit distance for all reads and ancient reads, breadth of coverage, deamination pattern, read length distribution, PMD scores, percent identity, table of top mapping reference and table of statistics. Authentication score = 10.

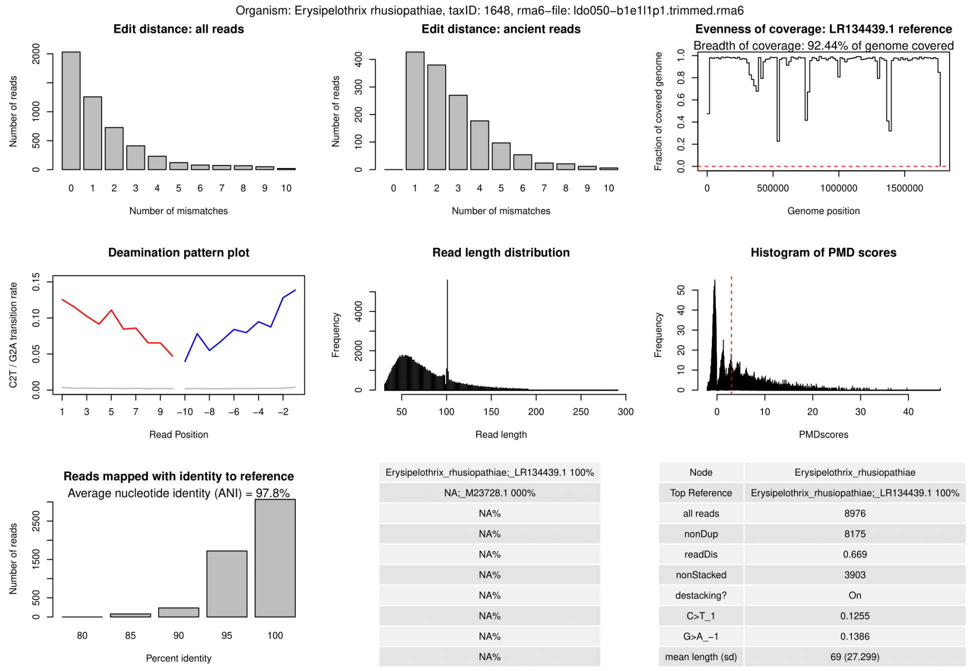

Fig. S10. Authentication plots for *Erysipelothrix rhusiopathiae*. Individual 37 (library: ldo050-b1e1l1p1) representing edit distance for all reads and ancient reads, breadth of coverage, deamination pattern, read

length distribution, PMD scores, percent identity, table of top mapping reference and table of statistics. Authentication score = 10.

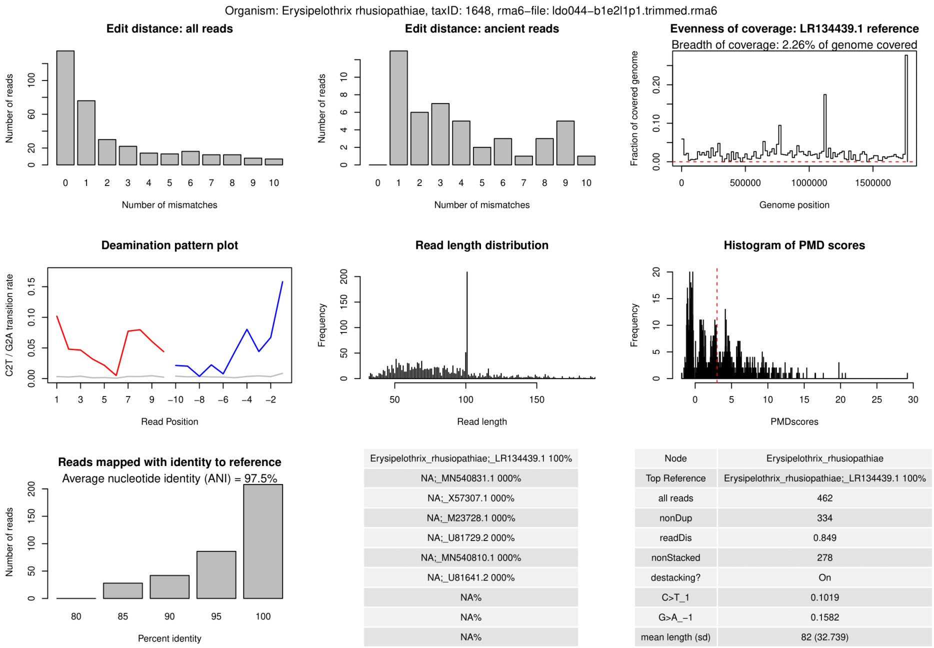

Fig. S11. Authentication plots for *Erysipelothrix rhusiopathiae*. Individual 7A (library: ldo044-b1e2l1p1) representing edit distance for all reads and ancient reads, breadth of coverage, deamination pattern, read length distribution, PMD scores, percent identity, table of top mapping reference and table of statistics. Authentication score = 9.

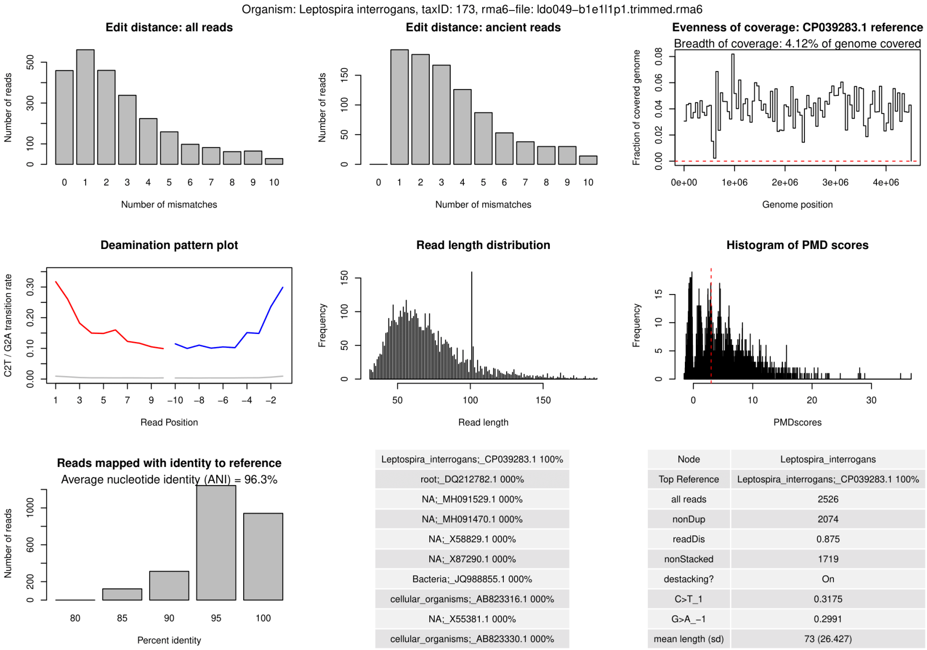

Fig. S12. Authentication plots for *Leptospira interrogans*. Individual 43 (library: ldo049-b1e1l1p1) representing edit distance for all reads and ancient reads, breadth of coverage, deamination pattern, read length distribution, PMD scores, percent identity, table of top mapping reference and table of statistics. Authentication score = 8.

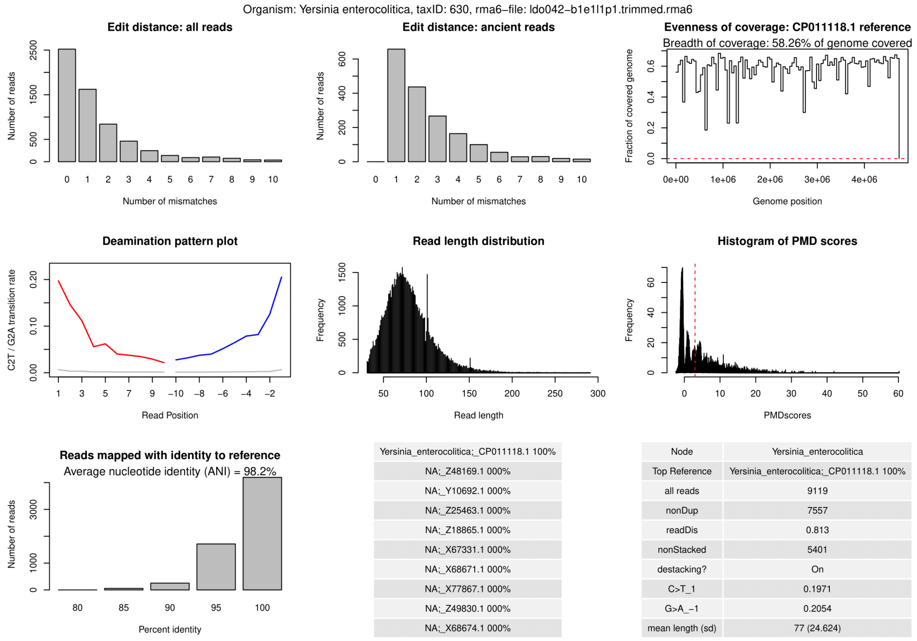

Fig. S13. Authentication plots for *Yersinia enterocolitica*. Individual 32 (library: ldo042-b1e1l1p1) representing edit distance for all reads and ancient reads, breadth of coverage, deamination pattern, read length distribution, PMD scores, percent identity, table of top mapping reference and table of statistics. Authentication score = 10.

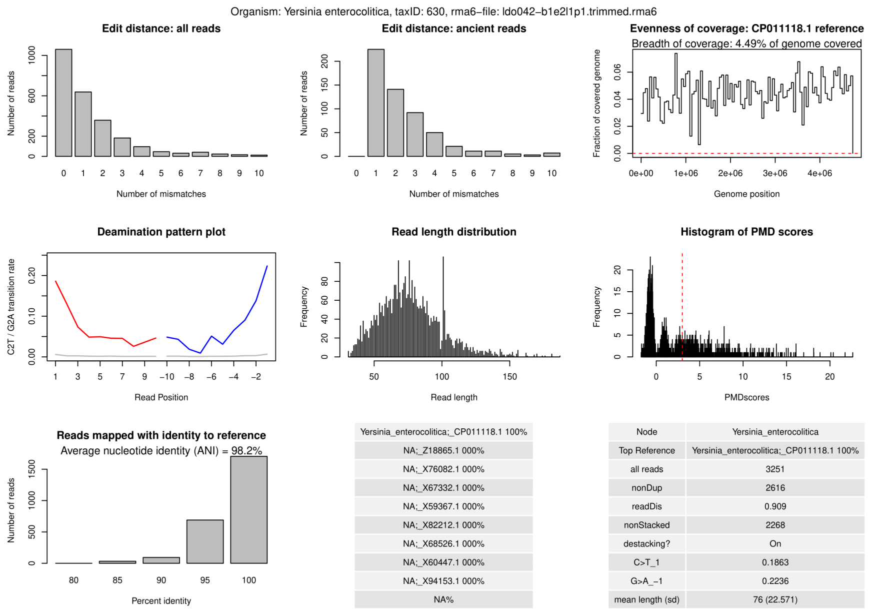

Fig. S14. Authentication plots for *Yersinia enterocolitica*. Individual 32 (library: ldo042-b1e2l1p1) representing edit distance for all reads and ancient reads, breadth of coverage, deamination pattern, read length distribution, PMD scores, percent identity, table of top mapping reference and table of statistics. Authentication score = 10.

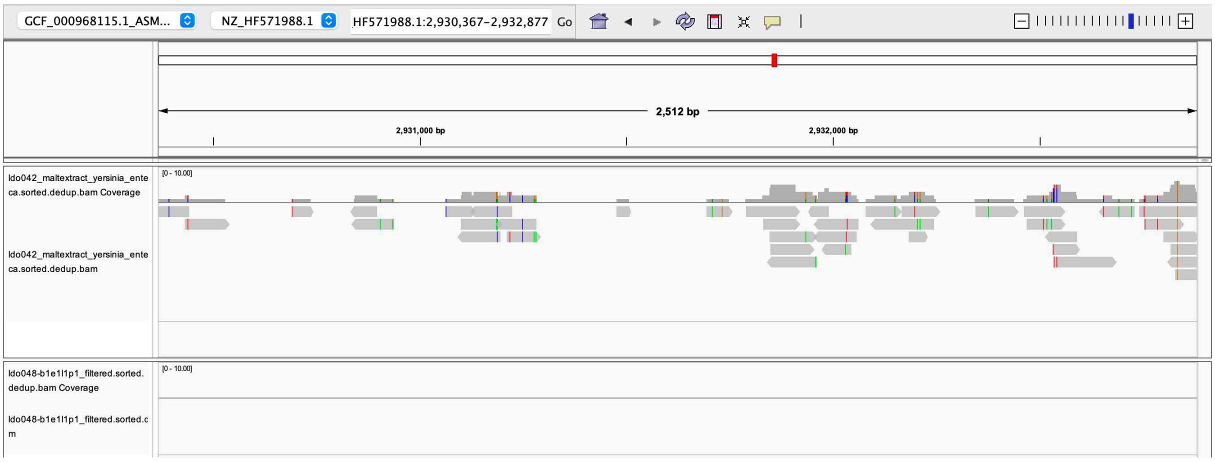

Fig. S15. Reads aligning to the virulence gene *invA* in individual 32 (ldo042-b1e1l1p1) using IGV (Integrative Genome Viewer). Individual 30 (ldo048-b1e1l1p1) actually harboring *Y. intermedia* and not *Y. enterocolitica* is kept as a control in the second window.

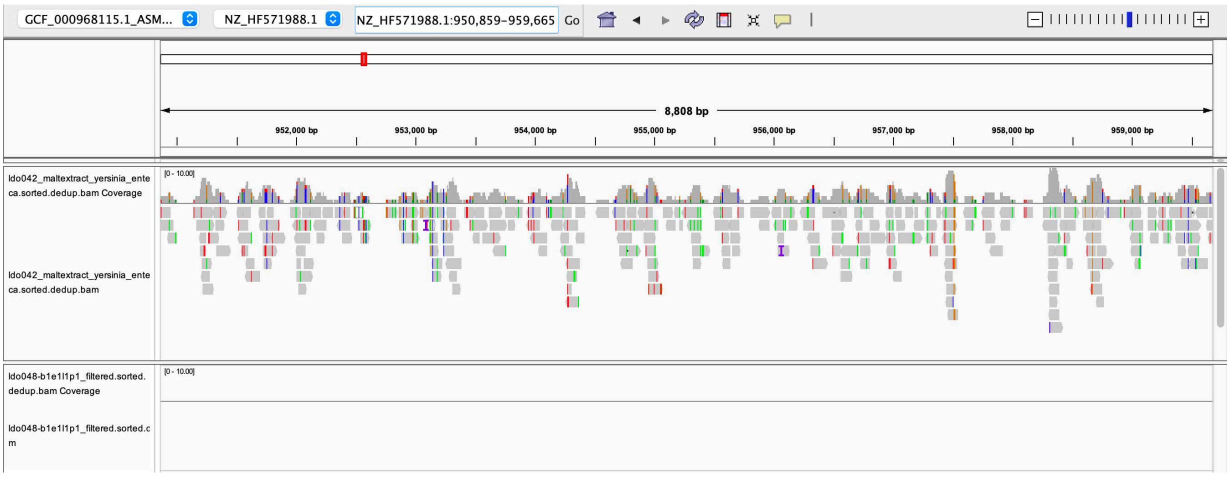

Fig. S16. Reads aligning to the virulence gene *yst* in individual 32 (ldo042-b1e1l1p1) using IGV (Integrative Genome Viewer). Individual 30 (ldo048-b1e1l1p1) actually harboring *Y. intermedia* and not *Y. enterocolitica* is kept as a control in the second window.

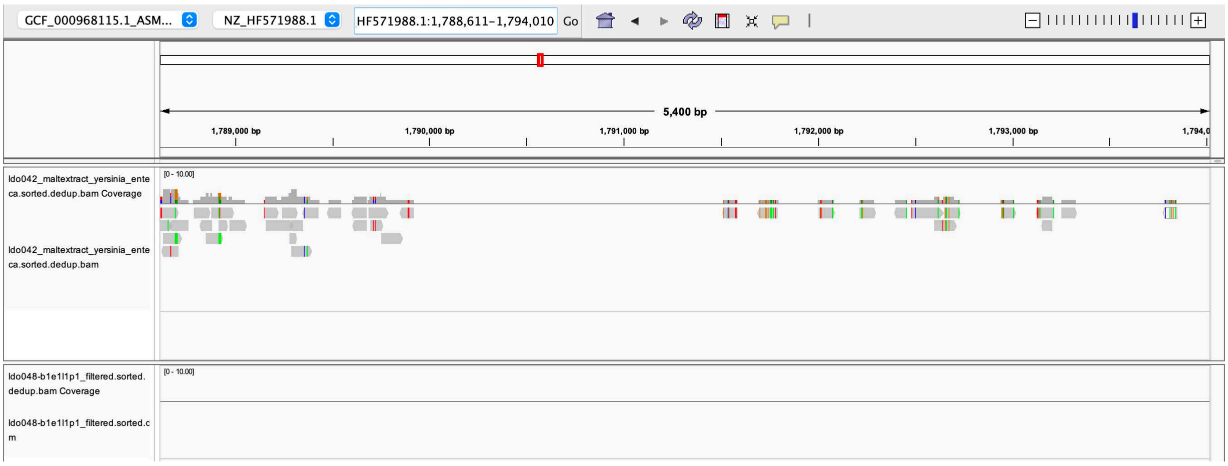

Fig. S17. Reads aligning to the virulence gene *myf* in individual 32 (ldo042-b1e1l1p1) using IGV (Integrative Genome Viewer). Individual 30 (ldo048-b1e1l1p1) actually harbouring *Y. intermedia* and not *Y. enterocolitica* is kept as a control in the second window.

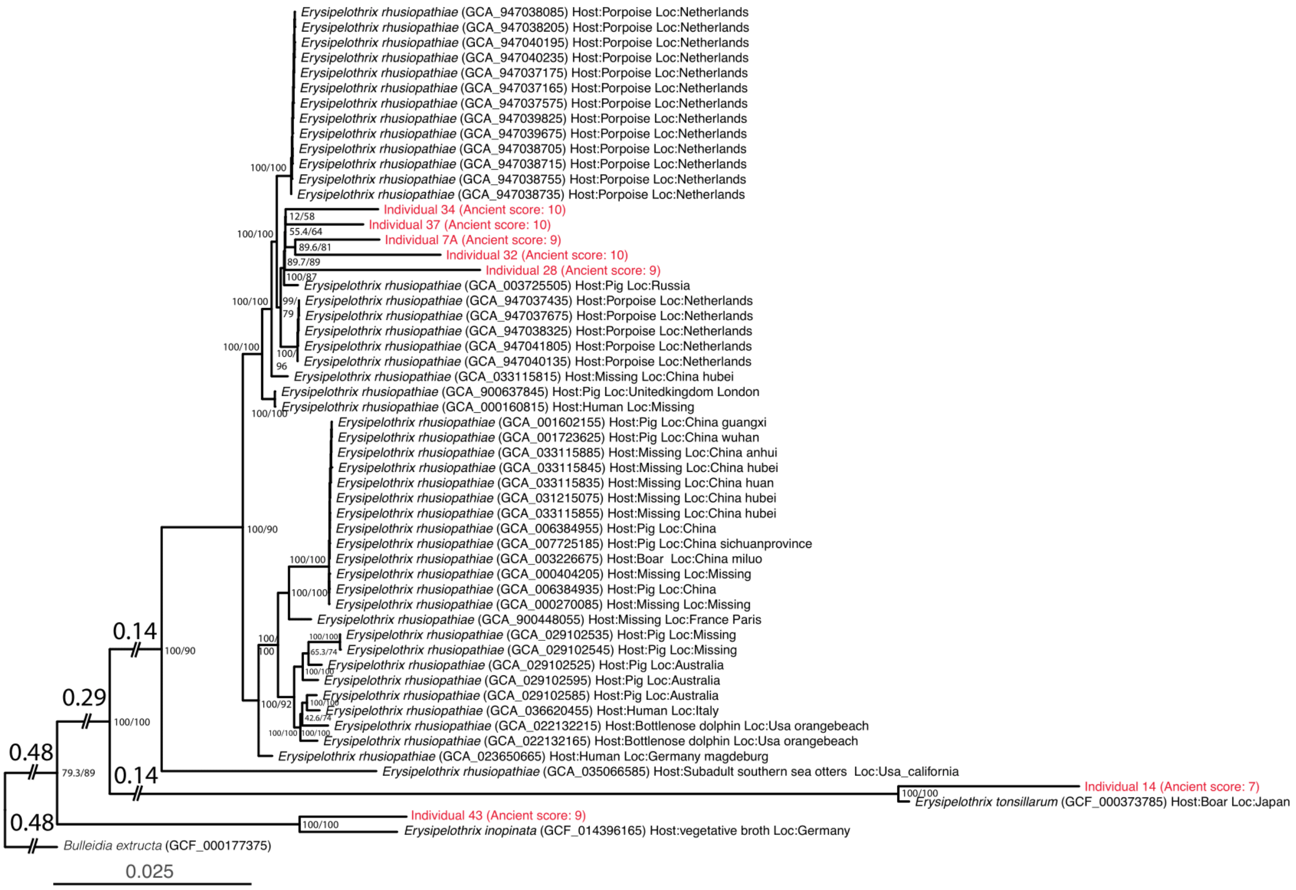

Fig. S18. Maximum likelihood tree of *Erysipelothrix* species. Sample names include species names, assembly accession numbers, host names, and sampling locations. Las Gobas samples are shown in red and the aMeta score of the read mapped against the *Erysipelothrix rhusiopathiae* genome is shown between parentheses. The tree is rooted to the species *Bulledia extructa*. Node confidence values (ultrafast-bootstrap support (%) / aLRT (%)) are shown at each node (bootstrap values in clades with too small genetic distances are not shown for practical display reasons). The scale bar shows the average number of nucleotide substitutions per site. Two slashes in branches represent branches that were shortened and the corresponding numbers represent the actual trimmed branch lengths.

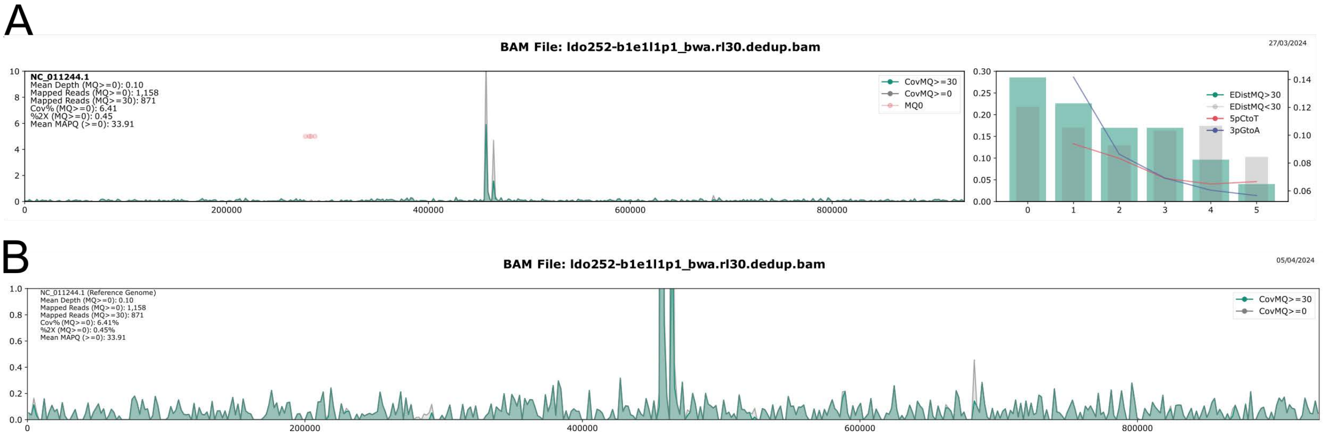

Fig. S19. Coverage plot for *Borrelia recurrentis* in individual 12. (**A**) Coverage plot and edit distance plot with damage patterns superposed. (**B**) Zoom coverage.

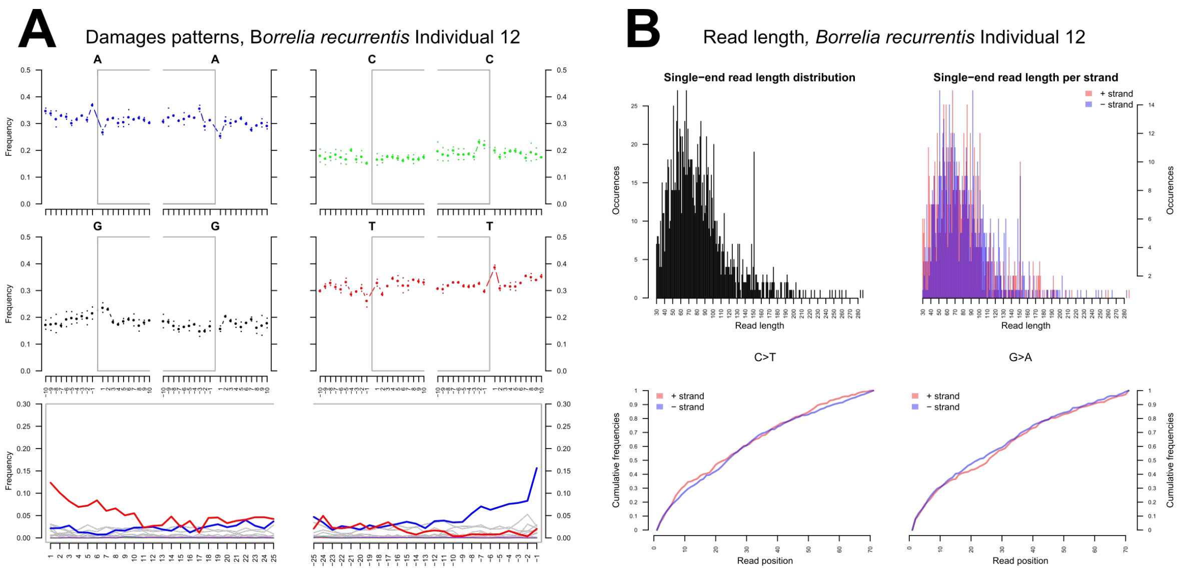

Fig. S20. Damages patterns and read length plots for *Borrelia recurrentis* in individual 12. (A) Damages patterns. (**B**) Read length.

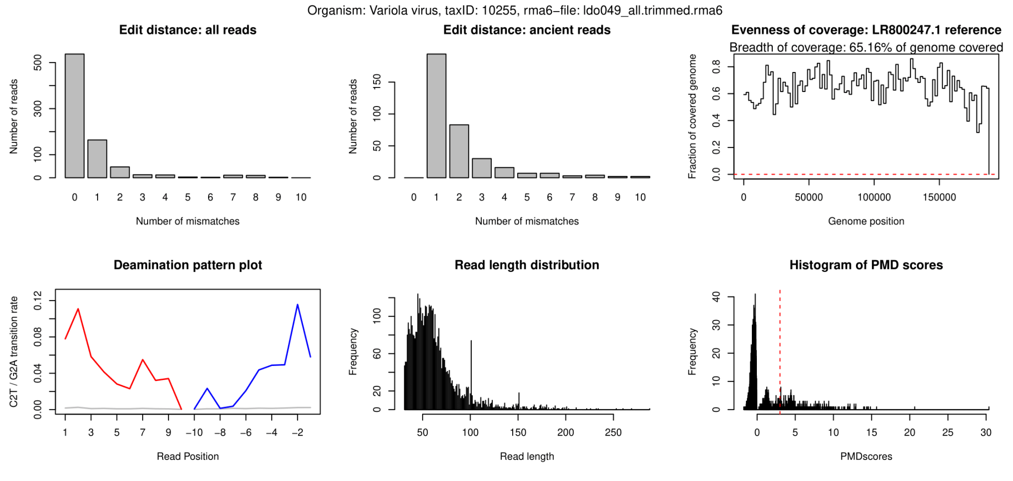

Variola_virus;_LR800247.1 100%
root;_U18338.1 013%
root;_X76268.1 011%
root;_U18339.1 007%
root;_X76265.1 007%
root;_X76267.1 006%
root;_X76266.1 005%
root;_X76264.1 004%
root;_X76263.1 003%
root;_X76262.1 001%

Node	Variola_virus
Top Reference	Variola_virus;_LR800247.1 100%
all reads	4302
nonDup	3719
readDis	0.233
nonStacked	353
destacking?	On
C>T_1	0.0779
G>A_-1	0.058
mean length (sd)	55 (21.888)

Fig. S21. Authentication plots for the ancient variola virus in individual 43. Plots representing edit distance for all reads and ancient reads, breadth of coverage, deamination pattern, read length distribution, PMD scores, percent identity, table of top mapping reference and table of statistics

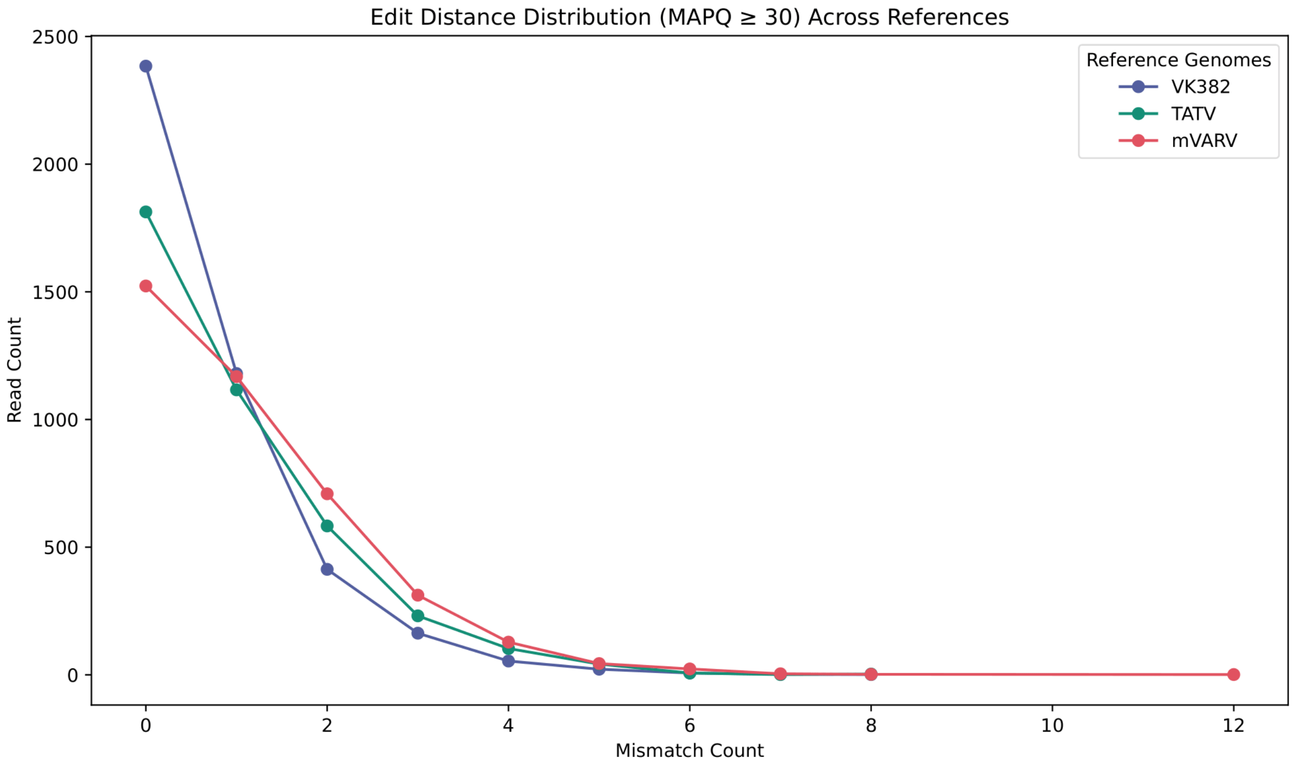

Fig. S22. Comparative edit distance plot for aVARV between reference genomes. Sequences from individual 43 were mapped using bwa aln non-competitively to the ancient consensus sequence for aVARV (VK382), the taterapox virus (closest virus to smallpox (TATV)) and the reference genome for modern smallpox strains (mVARV).

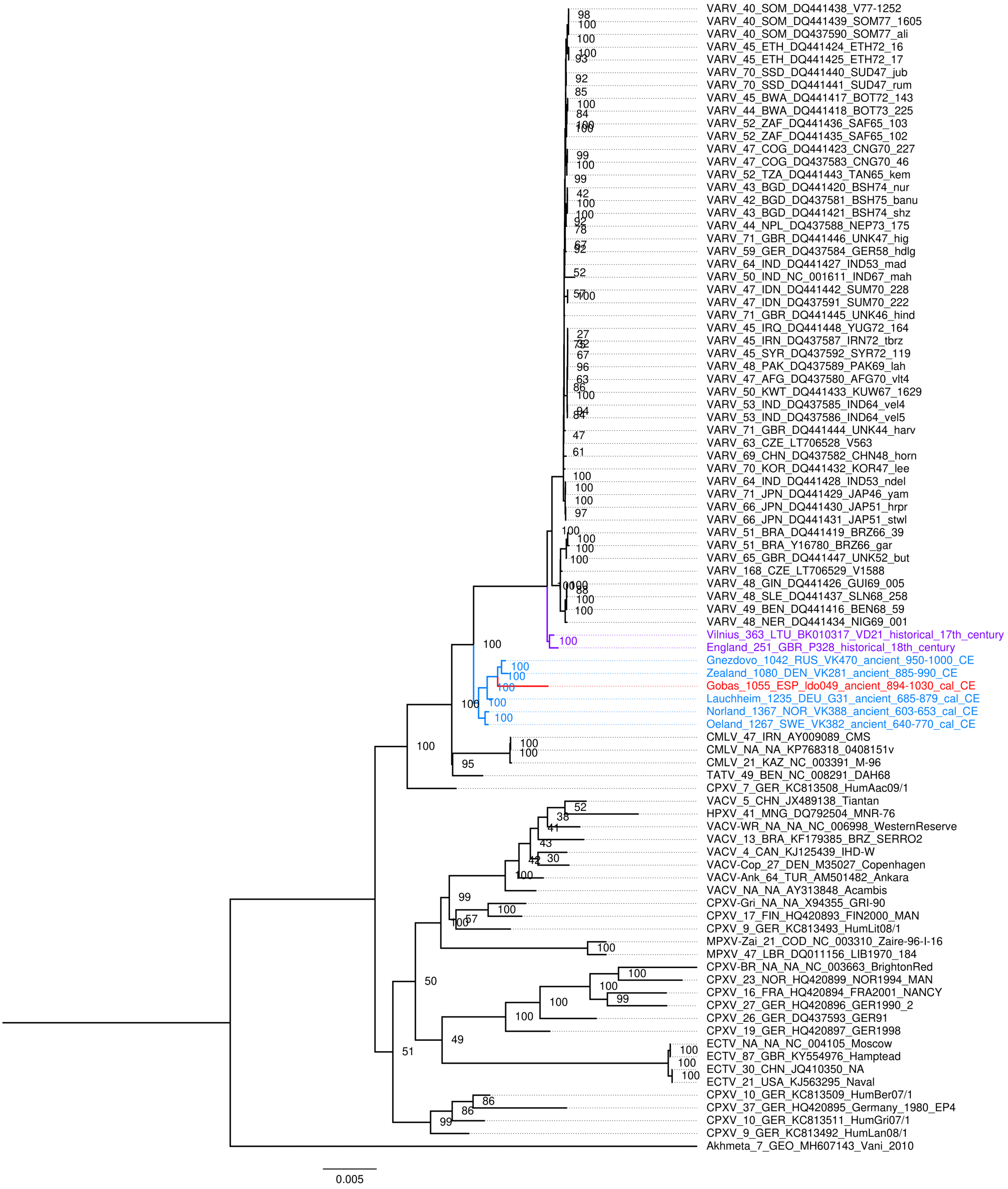

Figure S23. Maximum likelihood tree of poxviruses. All samples were mapped to VK382. Taxon name fields indicate: virus/sites, sample age relative to 2017, 3 letters country or region code, GenBank accession number when available, sample identifier and additional remarks. Ancient and historical samples are shown in blue and the Gobas sample is shown in red. The tree is midpoint-rooted. Node confidence values (bootstrap support) are shown at each node. The scale bar shows the average number of nucleotide substitutions per site.

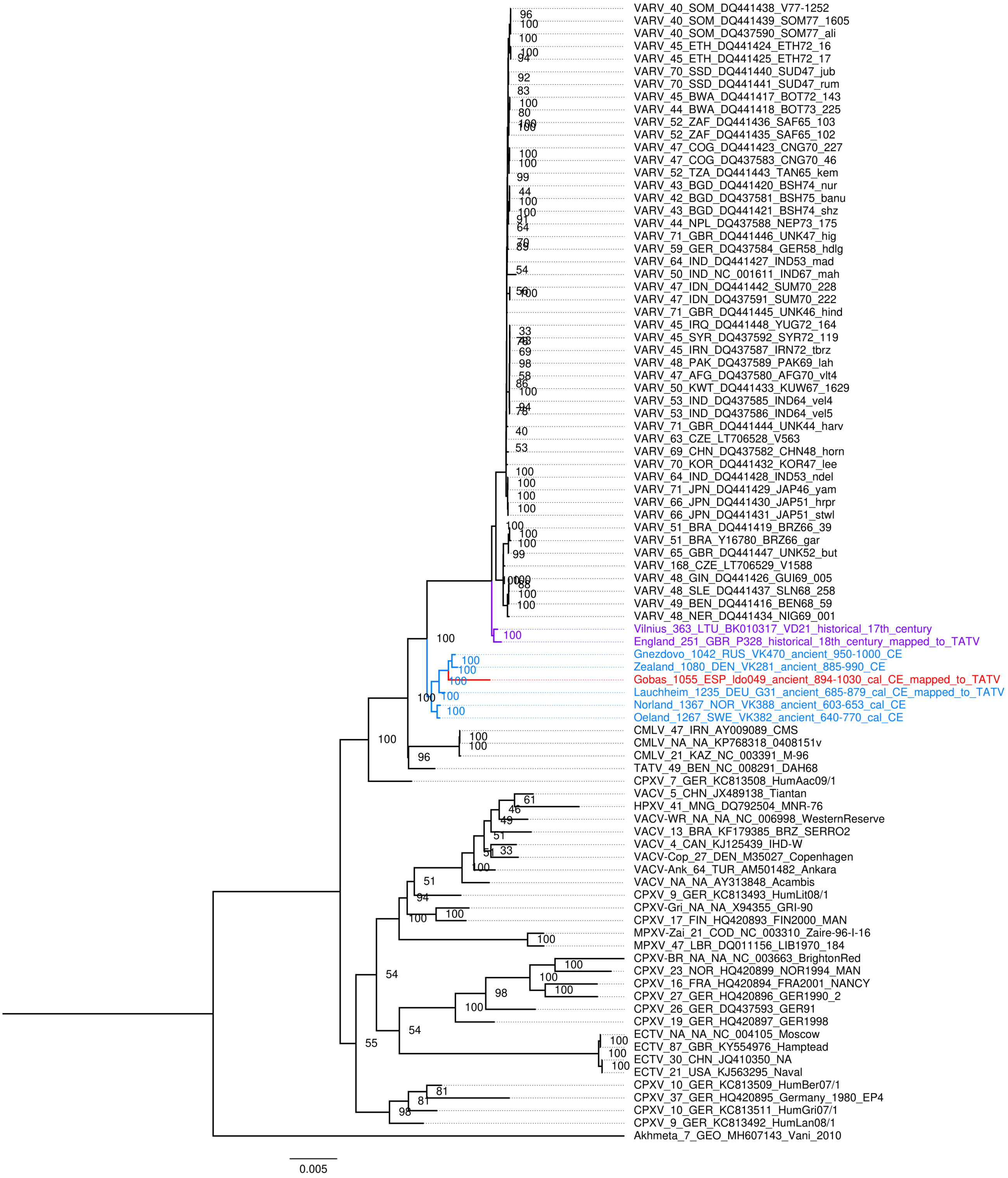

Figure S24. Maximum likelihood tree of poxviruses. All samples were mapped to VK382 except for 3 samples that were mapped to TATV to verify the certainty of their assignment. Taxon name fields indicate: virus/sites, sample age relative to 2017, 3 letters country or region code, GenBank accession number when available, sample identifier and additional remarks. Ancient and historical samples are shown in blue and the Gobas sample is shown in red. The tree is midpoint-rooted. Node confidence values (bootstrap support) are shown at each node. The scale bar shows the average number of nucleotide substitutions per site.

Table S1 to S12 (separate files).
Table S1. Sample information
Table S2. Sample statistic
Table S3. Y-chromosome haplogroups
Table S4. kinship results
Table S5. hapROH results
Table S6. krakenuniq pathogens
Table S7. Competitive mapping
Table S8. *E. rhusiopathiae* phylogeny
Table S9. Reference ancient individuals
Table S10. ADMIXTURE results
Table S11. qpAdm results
Table S12. *f*3 results

REFERENCES AND NOTES

1. I. Olalde, S. Mallick, N. Patterson, N. Rohland, V. Villalba-Mouco, M. Silva, K. Dulias, C. J. Edwards, F. Gandini, M. Pala, P. Soares, M. Ferrando-Bernal, N. Adamski, N. Broomandkhoshbacht, O. Cheronet, B. J. Culleton, D. Fernandes, A. M. Lawson, M. Mah, J. Oppenheimer, K. Stewardson, Z. Zhang, J. M. Jiménez Arenas, I. J. Toro Moyano, D. C. Salazar-García, P. Castanyer, M. Santos, J. Tremoleda, M. Lozano, P. García Borja, J. Fernández-Eraso, J. A. Mujika-Alustiza, C. Barroso, F. J. Bermúdez, E. Viguera Mínguez, J. Burch, N. Coromina, D. Vivó, A. Cebrià, J. M. Fullola, O. García-Puchol, J. I. Morales, F. X. Oms, T. Majó, J. M. Vergès, A. Díaz-Carvajal, I. Ollich-Castanyer, F. J. López-Cachero, A. M. Silva, C. Alonso-Fernández, G. Delibes de Castro, J. Jiménez Echevarría, A. Moreno-Márquez, G. Pascual Berlanga, P. Ramos-García, J. Ramos-Muñoz, E. Vijande Vila, G. Aguilella Arzo, Á. Esparza Arroyo, K. T. Lillios, J. Mack, J. Velasco-Vázquez, A. Waterman, L. Benítez de Lugo Enrich, M. Benito Sánchez, B. Agustí, F. Codina, G. de Prado, A. Estalrrich, Á. Fernández Flores, C. Finlayson, G. Finlayson, S. Finlayson, F. Giles-Guzmán, A. Rosas, V. Barciela González, G. García Atiénzar, M. S. Hernández Pérez, A. Llanos, Y. Carrión Marco, I. Collado Beneyto, D. López-Serrano, M. Sanz Tormo, A. C. Valera, C. Blasco, C. Liesau, P. Ríos, J. Daura, M. J. de Pedro Michó, A. A. Diez-Castillo, R. Flores Fernández, J. Francès Farré, R. Garrido-Pena, V. S. Gonçalves, E. Guerra-Doce, A. M. Herrero-Corral, J. Juan-Cabanilles, D. López-Reyes, S. B. McClure, M. Merino Pérez, A. Oliver Foix, M. Sanz Borràs, A. C. Sousa, J. M. Vidal Encinas, D. J. Kennett, M. B. Richards, K. Werner Alt, W. Haak, R. Pinhasi, C. Lalueza-Fox, D. Reich, The genomic history of the Iberian Peninsula over the past 8000 years. *Science* **363**, 1230–1234 (2019).

2. G. Gonzalez-Fortes, F. Tassi, E. Trucchi, K. Henneberger, J. L. A. Paijmans, D. Dıez-del-Molino, H. Schroeder, R. R. Susca, C. Barroso-Ruız, F. J. Bermudez, C. Barroso-Medina, A. M. S. Bettencourt, H. A. Sampaio, A. Grandal-d’Anglade, A. Salas, A. de Lombera-Hermida, R. F. Valcarce, M. Vaquero, S. Alonso, M. Lozano, X. P. Rodrıguez-Alvarez, C. Fernandez-Rodrıguez, A. Manica, M. Hofreiter, G. Barbujani, A western route of prehistoric human migration from Africa into the Iberian Peninsula. *Proc. R. Soc. B.* **286**, 20182288 (2019).

3. R. Fregel, F. L. Mendez, Y. Bokbot, D. Martin-Socas, M. D. Camalich-Massieu, J. Santana, J. Morales, M. C. Avila-Arcos, P. A. Underhill, B. Shapiro, G. L. Wojcik, M. Rasmussen, A. E. R. Soares, J. Kapp, A. Sockell, F. J. Rodriguez-Santos, A. Mikdad, A. Trujillo-Mederos, C. D. Bustamante, Ancient

genomes from North Africa evidence prehistoric migrations to the Maghreb from both the Levant and Europe. *Proc. Natl. Acad. Sci. U.S.A.* **115**, 6774–6779 (2018).

4. L. G. Simões, T. Günther, R. M. Martínez-Sánchez, J. C. Vera-Rodríguez, E. Iriarte, R. Rodríguez-Varela, Y. Bokbot, C. Valdiosera, M. Jakobsson, Northwest African Neolithic initiated by migrants from Iberia and Levant. *Nature* **618**, 550–556 (2023).

5. P. Moorjani, N. Patterson, J. N. Hirschhorn, A. Keinan, L. Hao, G. Atzmon, E. Burns, H. Ostrer, A. L. Price, D. Reich, The history of african gene flow into Southern Europeans, Levantines, and Jews. *PLOS Genet.* **7**, e1001373 (2011).

6. M. Silva, G. Oteo-García, R. Martiniano, J. Guimarães, M. Von Tersch, A. Madour, T. Shoeib, A. Fichera, P. Justeau, M. G. B. Foody, K. McGrath, A. Barrachina, V. Palomar, K. Dulias, B. Yau, F. Gandini, D. J. Clarke, A. Rosa, A. Brehm, A. Flaquer, T. Rito, A. Olivieri, A. Achilli, A. Torroni, A. Gómez-Carballa, A. Salas, J. Bryk, P. W. Ditchfield, M. Alexander, M. Pala, P. A. Soares, C. J. Edwards, M. B. Richards, Biomolecular insights into North African-related ancestry, mobility and diet in eleventh-century Al-Andalus. *Sci. Rep.* **11**, 18121 (2021).

7. F. García Fitz, La guerra contra el Islam peninsular en la Edad Media. (Madrid, Editorial Síntesis, 2019), p. 278.

8. L. A. G. Moreno, *España 702–719: La Conquista Musulmana* (Universidad de Sevilla, 2013).

9. A. García Sanjuán, *La conquista islámica de la península ibérica y la tergiversación del pasado: Del catastrofismo al negacionismo.* (Marcial Pons Historia, 2013), pp. 1–496.

10. A. Marsham, *The Umayyad World* (Routledge, 2020).

11. C. Bycroft, C. Fernandez-Rozadilla, C. Ruiz-Ponte, I. Quintela, Á. Carracedo, P. Donnelly, S. Myers, Patterns of genetic differentiation and the footprints of historical migrations in the Iberian Peninsula. *Nat. Commun.* **10**, 551 (2019).

12. A. Azkarate, Arqueología cristiana de la antigüedad tardía en Álava, Guipúzcoa y Vizcaya. *Diputacion Foral Alava* (Servicio de Publicaciones,1988).

13. A. Azkarate, J. Solaun, Excavaciones arqueológicas en el exterior de los conjuntos rupestres de Las Gobas (Laño, Burgos). *Arch. Esp. Arqueol.* **81**, 133–149 (2008).

14. A. Azkarate, J. L. Solaun, "Espacios domésticos, urbanos y rurales, de época medieval en el País Vasco" in *La Casa Medieval En La Península Ibérica* (Sílex, 2015), pp. 541–576.

15. I. Guede, L. A. Ortega, M. C. Zuluaga, A. Alonso-Olazabal, X. Murelaga, J. L. Solaun, I. Sanchez-Pinto, A. Azkarate, Isotopic evidence for the reconstruction of diet and mobility during village formation in the Early Middle Ages: Las Gobas (Burgos, northern Spain). *Archaeol. Anthropol. Sci.* **10**, 2047–2058 (2018).

16. L. Herrasti, I. Sánchez-Pinto, I. Etxeberria, A. Azkarate, F. Etxeberria, "Traumatic pathology and violence between the 7th and 11th centuries in the Hermitic Necropolis of Las Gobas (Laño, Treviño, Burgos)" in *Bioarchaeology of Injuries and Violence in Early Medieval Europe* (BAR Publishing, 2022), pp. 155-169.

17. P. Skoglund, J. Storå, A. Götherström, M. Jakobsson, Accurate sex identification of ancient human remains using DNA shotgun sequencing. *J. Archaeol. Sci.* **40**, 4477–4482 (2013).

18. P. Balaresque, G. R. Bowden, S. M. Adams, H.-Y. Leung, T. E. King, Z. H. Rosser, J. Goodwin, J.-P. Moisan, C. Richard, A. Millward, A. G. Demaine, G. Barbujani, C. Previderè, I. J. Wilson, C. Tyler-Smith, M. A. Jobling, A predominantly neolithic origin for european paternal lineages. *PLoS Biol.* **8**, e1000285 (2010).

19. W. Haak, I. Lazaridis, N. Patterson, N. Rohland, S. Mallick, B. Llamas, G. Brandt, S. Nordenfelt, E. Harney, K. Stewardson, Massive migration from the steppe was a source for Indo-European languages in Europe. *Nature* **522**, 207–211 (2015).

20. D. E. Platt, H. Artinian, F. Mouzaya, W. Khalil, F. G. Kamar, E. Matisoo-Smith, F. Calafell, N. N. Taleb, P. Zalloua, Autosomal genetics and Y-chromosome haplogroup L1b-M317 reveal Mount Lebanon Maronites as a persistently non-emigrating population. *Eur. J. Hum. Genet.* **29**, 581–592 (2021).

21. F. C. Ceballos, P. K. Joshi, D. W. Clark, M. Ramsay, J. F. Wilson, Runs of homozygosity: Windows into population history and trait architecture. *Nat. Rev. Genet.* **19**, 220–234 (2018).

22. H. Ringbauer, J. Novembre, M. Steinrücken, Parental relatedness through time revealed by runs of homozygosity in ancient DNA. *Nat. Commun.* **12**, 5425 (2021).

23. S. Mallick, A. Micco, M. Mah, H. Ringbauer, I. Lazaridis, I. Olalde, N. Patterson, D. Reich, The Allen Ancient DNA Resource (AADR): A curated compendium of ancient human genomes. *Sci. Data* **11**, 182 (2024).

24. Z. Pochon, N. Bergfeldt, E. Kırdök, M. Vicente, T. Naidoo, T. Van Der Valk, N. E. Altınışık, M. Krzewińska, L. Dalén, A. Götherström, aMeta: An accurate and memory-efficient ancient metagenomic profiling workflow. *Genome Biol.* **24**, 242 (2023).

25. F. P. Breitwieser, D. N. Baker, S. L. Salzberg, KrakenUniq: Confident and fast metagenomics classification using unique k-mer counts. *Genome Biol.* **19**, 198 (2018).

26. R. F. Irby, M. Kandula, R. Zadikany, R. L. Sandin, J. N. Greene, Yarrowia lipolytica as normal human flora. *Infect. Dis. Clin. Pract.* **22**, 207–209 (2014).

27. I. F. Escapa, T. Chen, Y. Huang, P. Gajare, F. E. Dewhirst, K. P. Lemon, New insights into human nostril microbiome from the expanded human oral microbiome database (eHOMD): A resource for the microbiome of the human aerodigestive tract. *Msystems* **3**, 10–1128 (2018).

28. B. Krawczyk, P. Wityk, M. Gałęcka, M. Michalik, The many faces of enterococcus spp.—Commensal, probiotic and opportunistic pathogen. *Microorganisms* **9**, 1900 (2021).

29. J. N. Weiser, D. M. Ferreira, J. C. Paton, Streptococcus pneumoniae: Transmission, colonization and invasion. *Nat. Rev. Microbiol.* **16**, 355–367 (2018).

30. A. Herbig, F. Maixner, K. I. Bos, A. Zink, J. Krause, D. H. Huson, MALT: Fast alignment and analysis of metagenomic DNA sequence data applied to the Tyrolean Iceman. bioRxiv 050559 [Preprint] (2016). https://doi.org/10.1101/050559.

31. M. Guellil, O. Kersten, A. Namouchi, E. L. Bauer, M. Derrick, A. Ø. Jensen, N. C. Stenseth, B. Bramanti, Genomic blueprint of a relapsing fever pathogen in 15th century Scandinavia. *Proc. Natl. Acad. Sci. U.S.A.* **115**, 10422–10427 (2018).

32. B. Mühlemann, L. Vinner, A. Margaryan, H. Wilhelmson, C. De La Fuente Castro, M. E. Allentoft, P. De Barros Damgaard, A. J. Hansen, S. Holtsmark Nielsen, L. M. Strand, J. Bill, A. Buzhilova, T. Pushkina, C. Falys, V. Khartanovich, V. Moiseyev, M. L. S. Jørkov, P. Østergaard Sørensen, Y. Magnusson, I. Gustin, H. Schroeder, G. Sutter, G. L. Smith, C. Drosten, R. A. M. Fouchier, D. J. Smith, E. Willerslev, T. C. Jones, M. Sikora, Diverse variola virus (smallpox) strains were widespread in northern Europe in the Viking Age. *Science* **369**, eaaw8977 (2020).

33. D. H. Alexander, J. Novembre, K. Lange, Fast model-based estimation of ancestry in unrelated individuals. *Genome Res.* **19**, 1655–1664 (2009).

34. J. M. R. Punzón, *El Mundo Funerario Rural En La Provincia de Granada Durante La Antigüedad Tardía* (Universidad de Granada, 2004).

35. J. L. Boone, Tribalism, ethnicity, and islamization in the Baixo Alentejo of Portugal: Preliminary results of investigation into transitional period (AD 550-850) rural settlements. *Era-Arqueol. Rev. Divulg. Cient. Estud. Arqueol.* **4**, 104–121 (2001).

36. T. Glick, “Islamic and Christian Spain in the Early Middle Ages: Second” in *Islamic and Christian Spain in the Early Middle Ages* (Brill, 2005).

37. H. Kennedy, *Muslim Spain and Portugal: A Political History of al-Andalus* (Routledge, 2014).

38. S. Inskip, Islam in Iberia or Iberian Islam: Bioarchaeology and the analysis of emerging Islamic identity in Early Medieval Iberia. *J. Post Classic. Archaeol.* **3**, 63–93 (2013).

39. N. Patterson, P. Moorjani, Y. Luo, S. Mallick, N. Rohland, Y. Zhan, T. Genschoreck, T. Webster, D. Reich, Ancient admixture in human history. *Genetics* **192**, 1065–1093 (2012).

40. R. Rodríguez-Varela, T. Günther, M. Krzewińska, J. Storå, T. H. Gillingwater, M. MacCallum, J. L. Arsuaga, K. Dobney, C. Valdiosera, M. Jakobsson, A. Götherström, L. Girdland-Flink, Genomic

analyses of pre-european conquest human remains from the canary islands reveal close affinity to modern North Africans. *Curr. Biol.* **27**, 3396–3402.e5 (2017).

41. J. G. Serrano, The genomic history of the indigenous people of the Canary Islands. *Nat. Commun.* **14**, 4641 (2023).

42. L. R. Arauna, J. Mendoza-Revilla, A. Mas-Sandoval, H. Izaabel, A. Bekada, S. Benhamamouch, K. Fadhlaoui-Zid, P. Zalloua, G. Hellenthal, D. Comas, Recent historical migrations have shaped the gene pool of Arabs and Berbers in North Africa. *Mol. Biol. Evol.* **34**, 318–329 (2016).

43. E. Prevedorou, M. D.-Z. Bonilla, A. Romero, J. E. Buikstra, M. P. de Miguel Ibáñez, K. J. Knudson, Residential mobility and dental decoration in Early Medieval Spain: Results from the eighth century site of Plaza del Castillo, Pamplona. *Dent. Anthropol. J.* **23**, 42–52 (2010).

44. L. Jesús, E. P. de Garayo, "Al-Andalus?` en la periferia de Vasconia?: Sistemas de dominación de bilad Banbaluna y de Alaba wa-l-Qila en la octava centuria" in *Vasconia En La Alta Edad Media, 450-1000: Poderes y Comunidades Rurales En El Norte Penínsular* (Servicio de Publicaciones, 2011), pp. 55–70.

45. A. Flores-Bello, F. Bauduer, J. Salaberria, B. Oyharçabal, F. Calafell, J. Bertranpetit, L. Quintana-Murci, D. Comas, Genetic origins, singularity, and heterogeneity of Basques. *Curr. Biol.* **31**, 2167–2177.e4 (2021).

46. M. Romney, S. Cheung, V. Montessori, Erysipelothrix rhusiopathiae endocarditis and presumed osteomyelitis. *Can. J. Infect. Dis.* **12**, 912086 (1900).

47. M. Spiteri, A. Taylor-Robinson, *Erysipelothrix rhusiopathiae*: An important cause of bacterial disease in farmed pigs and an occupational pathogen of humans. *Int. J. Clin. Med. Microbiol.* **3**, 134 (2018).

48. T. L. Forde, K. Orsel, R. N. Zadoks, R. Biek, L. G. Adams, F. van der Meer, S. J. Kutz, Bacterial genomics reveal the complex epidemiology of an emerging pathogen in arctic and boreal ungulates. *Front. Microbiol.* **7**, 226383 (2016).

49. D. Chandler, J. Craven, Persistence and distribution of *Erysipelothrix rhusiopathiae* and bacterial indicator organisms on land used for disposal of piggery effluent. *J. Appl. Microbiol.* **48**, 367–375 (1980).

50. M. Sikora, E. Canteri, A. Fernandez-Guerra, N. Oskolkov, R. Agren, L. Hansson, E. K. Irving-Pease, B. Mühlemann, S. Holtsmark Nielsen, G. Scorrano, M. E. Allentoft, F. V. Seersholm, H. Schroeder, C. Gaunitz, J. Stenderup, L. Vinner, T. C. Jones, B. Nystedt, J. Parkhill, L. Fugger, F. Racimo, K. Kristiansen, A. K. N. Iversen, E. Willerslev, The landscape of ancient human pathogens in Eurasia from the Stone Age to historical times. bioRxiv 561165 [Preprint] (2023). https://doi.org/10.1101/2023.10.06.561165.

51. N. Bergfeldt, E. Kırdök, N. Oskolkov, C. Mirabello, P. Unneberg, H. Malmström, M. Fraser, F. Sanchez-Quinto, R. Jorgensen, B. Skar, Identification of microbial pathogens in Neolithic Scandinavian humans. *Sci. Rep.* **14**, 5630 (2024).

52. J. H. Bonczarowska, J. Susat, B. Mühlemann, I. Jasch-Boley, S. Brather, B. Höke, S. Brather-Walter, V. Schoenenberg, J. Scheschkewitz, G. Graenert, D. Krausse, M. Francken, T. C. Jones, J. Wahl, A. Nebel, B. Krause-Kyora, Pathogen genomics study of an early medieval community in Germany reveals extensive co-infections. *Genome Biol.* **23**, 250 (2022).

53. C. Thèves, E. Crubézy, P. Biagini, History of smallpox and its spread in human populations. *Microbiol. Spectr.* **4**, 4.4.05 (2016).

54. F. Fenner, D. Henderson, I. Arita, Z. Ježek, I. D. Ladnyi, *Smallpox and its Eradication* (World Health Organization, 1987), p. 210.

55. E. C. Griffiths, A. B. Pedersen, A. Fenton, O. L. Petchey, The nature and consequences of coinfection in humans. *J. Infect.* **63**, 200–206 (2011).

56. J. Dabney, M. Knapp, I. Glocke, M.-T. Gansauge, A. Weihmann, B. Nickel, C. Valdiosera, N. Garcia, S. Paabo, J.-L. Arsuaga, M. Meyer, Complete mitochondrial genome sequence of a Middle Pleistocene cave bear reconstructed from ultrashort DNA fragments. *Proc. Natl. Acad. Sci. U.S.A.* **110**, 15758–15763 (2013).

57. M. Meyer, M. Kircher, Illumina sequencing library preparation for highly multiplexed target capture and sequencing. *Cold Spring Harb. Protoc.* **2010**, pdb.prot5448 (2010).

58. H. Li, R. Durbin, Fast and accurate short read alignment with Burrows–Wheeler transform. *Bioinformatics* **25**, 1754–1760 (2009).

59. H. Li, B. Handsaker, A. Wysoker, T. Fennell, J. Ruan, N. Homer, G. Marth, G. Abecasis, R. Durbin, The sequence alignment/map format and SAMtools. *Bioinformatics* **25**, 2078–2079 (2009).

60. M. Kircher, "Analysis of high-throughput ancient DNA sequencing data" in *Ancient DNA*, B. Shapiro, M. Hofreiter, Eds. (Humana Press, 2012), vol. 840, pp. 197–228.

61. P. Skoglund, H. Malmstrom, M. Raghavan, J. Stora, P. Hall, E. Willerslev, M. T. P. Gilbert, A. Gotherstrom, M. Jakobsson, Origins and genetic legacy of neolithic farmers and hunter-gatherers in Europe. *Science* **336**, 466–469 (2012).

62. P. Skoglund, B. H. Northoff, M. V. Shunkov, A. P. Derevianko, S. Pääbo, J. Krause, M. Jakobsson, Separating endogenous ancient DNA from modern day contamination in a Siberian Neandertal. *Proc. Natl. Acad. Sci. U.S.A.* **111**, 2229–2234 (2014).

63. R. E. Green, A.-S. Malaspinas, J. Krause, A. W. Briggs, P. L. F. Johnson, C. Uhler, M. Meyer, J. M. Good, T. Maricic, U. Stenzel, K. Prüfer, M. Siebauer, H. A. Burbano, M. Ronan, J. M. Rothberg, M. Egholm, P. Rudan, D. Brajković, Ž. Kućan, I. Gušić, M. Wikström, L. Laakkonen, J. Kelso, M. Slatkin, S. Pääbo, A complete neandertal mitochondrial genome sequence determined by high-throughput sequencing. *Cell* **134**, 416–426 (2008).

64. Q. Fu, A. Mittnik, P. L. F. Johnson, K. Bos, M. Lari, R. Bollongino, C. Sun, L. Giemsch, R. Schmitz, J. Burger, A. M. Ronchitelli, F. Martini, R. G. Cremonesi, J. Svoboda, P. Bauer, D. Caramelli, S. Castellano, D. Reich, S. Pääbo, J. Krause, A revised timescale for human evolution based on ancient mitochondrial genomes. *Curr. Biol.* **23**, 553–559 (2013).

65. T. S. Korneliussen, A. Albrechtsen, R. Nielsen, ANGSD: Analysis of next generation sequencing data. *BMC Bioinformatics* **15**, 356 (2014).

66. M. Rasmussen, X. Guo, Y. Wang, K. E. Lohmueller, S. Rasmussen, A. Albrechtsen, L. Skotte, S. Lindgreen, M. Metspalu, T. Jombart, T. Kivisild, W. Zhai, A. Eriksson, A. Manica, L. Orlando, F. M. De La Vega, S. Tridico, E. Metspalu, K. Nielsen, M. C. Avila-Arcos, J. V. Moreno-Mayar, C. Muller, J. Dortch, M. T. P. Gilbert, O. Lund, A. Wesolowska, M. Karmin, L. A. Weinert, B. Wang, J. Li, S. Tai, F. Xiao, T. Hanihara, G. van Driem, A. R. Jha, F.-X. Ricaut, P. de Knijff, A. B. Migliano, I. Gallego Romero, K. Kristiansen, D. M. Lambert, S. Brunak, P. Forster, B. Brinkmann, O. Nehlich, M. Bunce, M. Richards, R. Gupta, C. D. Bustamante, A. Krogh, R. A. Foley, M. M. Lahr, F. Balloux, T. Sicheritz-Ponten, R. Villems, R. Nielsen, J. Wang, E. Willerslev, An aboriginal australian genome reveals separate human dispersals into Asia. *Science* **334**, 94–98 (2011).

67. L. C. Reimer, J. Sardà Carbasse, J. Koblitz, C. Ebeling, A. Podstawka, J. Overmann, BacDive in 2022: The knowledge base for standardized bacterial and archaeal data. *Nucleic Acids Res.* **50**, D741–D746 (2022).

68. Microbe Atlas Project Database; https://microbeatlas.org.

69. T. Sasi Jyothsna, L. Tushar, C. Sasikala, C. V. Ramana, *Paraclostridium benzoelyticum* gen. nov., sp. nov., isolated from marine sediment and reclassification of *Clostridium bifermentans* as *Paraclostridium bifermentans* comb. nov. Proposal of a new genus *Paeniclostridium* gen. nov. to accommodate *Clostridium sordellii* and *Clostridium ghonii*. *Int. J. Syst. Evol. Microbiol.* **66**, 1268–1274 (2016).

70. V. G. Krishnaswamy, R. Sridharan, P. S. Kumar, M. J. Fathima, Cellulase enzyme catalyst producing bacterial strains from vermicompost and its application in low-density polyethylene degradation. *Chemosphere* **288**, 132552 (2022).

71. H. Thorvaldsdottir, J. T. Robinson, J. P. Mesirov, Integrative Genomics Viewer (IGV): High-performance genomics data visualization and exploration. *Brief. Bioinform.* **14**, 178–192 (2013).

72. M. Guellil, MeriamGuellil/aDNA-BAMPlotter:, version v2.0.1. *Zenodo* (2021); https://doi.org/10.5281/zenodo.5702679.

73. H. Jónsson, A. Ginolhac, M. Schubert, P. L. F. Johnson, L. Orlando, mapDamage2.0: Fast approximate Bayesian estimates of ancient DNA damage parameters. *Bioinformatics* **29**, 1682–1684 (2013).

74. R. Hübler, F. M. Key, C. Warinner, K. I. Bos, J. Krause, A. Herbig, HOPS: Automated detection and authentication of pathogen DNA in archaeological remains. *Genome Biol.* **20**, 280 (2019).

75. B. Langmead, S. L. Salzberg, Fast gapped-read alignment with Bowtie 2. *Nat. Methods* **9**, 357–359 (2012).

76. A. T. Duggan, M. F. Perdomo, D. Piombino-Mascali, S. Marciniak, D. Poinar, M. V. Emery, J. P. Buchmann, S. Duchêne, R. Jankauskas, M. Humphreys, G. B. Golding, J. Southon, A. Devault, J.-M. Rouillard, J. W. Sahl, O. Dutour, K. Hedman, A. Sajantila, G. L. Smith, E. C. Holmes, H. N. Poinar, 17th century variola virus reveals the recent history of smallpox. *Curr. Biol.* **26**, 3407–3412 (2016).

77. G. Ferrari, J. Neukamm, H. T. Baalsrud, A. M. Breidenstein, M. Ravinet, C. Phillips, F. Rühli, A. Bouwman, V. J. Schuenemann, Variola virus genome sequenced from an eighteenth-century museum specimen supports the recent origin of smallpox. *Philos. Trans. R. Soc. B* **375**, 20190572 (2020).

78. S. Chen, Y. Zhou, Y. Chen, J. Gu, fastp: An ultra-fast all-in-one FASTQ preprocessor. *Bioinformatics* **34**, i884–i890 (2018).

79. T. Magoč, S. L. Salzberg, FLASH: Fast length adjustment of short reads to improve genome assemblies. *Bioinformatics* **27**, 2957–2963 (2011).

80. S. Andrews, FastQC: A quality control tool for high throughput sequence data. BibSonomy. (accessed 17 March 2022) www.bioinformatics.babraham.ac.uk/projects/fastqc/ (2010).

81. P. Danecek, J. K. Bonfield, J. Liddle, J. Marshall, V. Ohan, M. O. Pollard, A. Whitwham, T. Keane, S. A. McCarthy, R. M. Davies, H. Li, Twelve years of SAMtools and BCFtools. *Gigascience* **10**, giab008 (2021).

82. G. Jun, M. K. Wing, G. R. Abecasis, H. M. Kang, An efficient and scalable analysis framework for variant extraction and refinement from population-scale DNA sequence data. *Genome Res.* **25**, 918–925 (2015).

83. B. Q. Minh, H. A. Schmidt, O. Chernomor, D. Schrempf, M. D. Woodhams, A. von Haeseler, R. Lanfear, IQ-TREE 2: New models and efficient methods for phylogenetic inference in the genomic era. *Mol. Biol. Evol.* **37**, 1530–1534 (2020).

84. S. Kalyaanamoorthy, B. Q. Minh, T. K. Wong, A. Von Haeseler, L. S. Jermiin, ModelFinder: Fast model selection for accurate phylogenetic estimates. *Nat. Methods* **14**, 587–589 (2017).

85. I. Minkin, P. Medvedev, Scalable multiple whole-genome alignment and locally collinear block construction with SibeliaZ. *Nat. Commun.* **11**, 6327 (2020).

86. A. Kloss-Brandstätter, D. Pacher, S. Schönherr, H. Weissensteiner, R. Binna, G. Specht, F. Kronenberg, HaploGrep: A fast and reliable algorithm for automatic classification of mitochondrial DNA haplogroups. *Hum. Mutat.* **32**, 25–32 (2011).

87. H. Weissensteiner, D. Pacher, A. Kloss-Brandstätter, L. Forer, G. Specht, H.-J. Bandelt, F. Kronenberg, A. Salas, S. Schönherr, HaploGrep 2: Mitochondrial haplogroup classification in the era of high-throughput sequencing. *Nucleic Acids Res.* **44**, W58–W63 (2016).

88. M. van Oven, M. Kayser, Updated comprehensive phylogenetic tree of global human mitochondrial DNA variation. *Hum. Mutat.* **30**, E386–E394 (2009).

89. R. Martiniano, B. D. Sanctis, P. Hallast, R. Durbin, Placing ancient DNA sequences into reference phylogenies. *Mol. Biol. Evol.* **39**, msac017 (2022).

90. M. Lipatov, K. Sanjeev, R. Patro, K. Veeramah, Maximum likelihood estimation of biological relatedness from low coverage sequencing data. bioRxiv 023374 [Preprint] (2015). https://doi.org/10.1101/023374.

91. D. Popli, S. Peyrégne, B. M. Peter, KIN: A method to infer relatedness from low-coverage ancient DNA. *Genome Biol.* **24**, 10 (2023).

92. K. Hanghøj, I. Moltke, P. A. Andersen, A. Manica, T. S. Korneliussen, Fast and accurate relatedness estimation from high-throughput sequencing data in the presence of inbreeding. *Gigascience* **8**, giz034 (2019).

93. A. L. Severson, T. S. Korneliussen, I. Moltke, LocalNgsRelate: A software tool for inferring IBD sharing along the genome between pairs of individuals from low-depth NGS data. *Bioinformatics* **38**, 1159–1161 (2022).

94. L. Pagani, D. J. Lawson, E. Jagoda, A. Mörseburg, A. Eriksson, M. Mitt, F. Clemente, G. Hudjashov, M. DeGiorgio, L. Saag, J. D. Wall, A. Cardona, R. Mägi, M. A. W. Sayres, S. Kaewert, C. Inchley, C. L. Scheib, M. Järve, M. Karmin, G. S. Jacobs, T. Antao, F. M. Iliescu, A. Kushniarevich, Q. Ayub, C. Tyler-Smith, Y. Xue, B. Yunusbayev, K. Tambets, C. B. Mallick, L. Saag, E. Pocheshkhova, G. Andriadze, C. Muller, M. C. Westaway, D. M. Lambert, G. Zoraqi, S. Turdikulova, D. Dalimova, Z. Sabitov, G. N. N. Sultana, J. Lachance, S. Tishkoff, K. Momynaliev, J. Isakova, L. D. Damba, M. Gubina, P. Nymadawa, I. Evseeva, L. Atramentova, O. Utevska, F.-X. Ricaut, N. Brucato, H. Sudoyo, T. Letellier, M. P. Cox, N. A. Barashkov, V. Škaro, L. Mulahasanović, D. Primorac, H. Sahakyan, M. Mormina, C. A. Eichstaedt, D. V. Lichman, S. Abdullah, G. Chaubey, J. T. S. Wee, E. Mihailov, A. Karunas, S. Litvinov, R. Khusainova, N. Ekomasova, V. Akhmetova, I. Khidiyatova, D. Marjanović, L. Yepiskoposyan, D. M. Behar, E. Balanovska, A. Metspalu, M. Derenko, B. Malyarchuk, M. Voevoda, S. A. Fedorova, L. P. Osipova, M. M. Lahr, P. Gerbault, M. Leavesley, A. B. Migliano, M. Petraglia, O. Balanovsky, E. K. Khusnutdinova, E. Metspalu, M. G. Thomas, A. Manica, R. Nielsen, R. Villems, E. Willerslev, T. Kivisild, M. Metspalu, Genomic analyses inform on migration events during the peopling of Eurasia. *Nature* **538**, 238–242 (2016).

95. 1000 Genomes Project Consortium, A global reference for human genetic variation. *Nature* **526**, 68–74 (2015).

96. R. Yaka, I. Mapelli, D. Kaptan, A. Doğu, M. Chyleński, Ö. D. Erdal, D. Koptekin, K. B. Vural, A. Bayliss, C. Mazzucato, E. Fer, S. S. Çokoğlu, V. K. Lagerholm, M. Krzewińska, C. Karamurat, H. C. Gemici, A. Sevkar, N. D. Dağtaş, G. M. Kılınç, D. Adams, A. R. Munters, E. Sağlıcan, M. Milella, E. M. J. Schotsmans, E. Yurtman, M. Çetin, S. Yorulmaz, N. E. Altınışık, A. Ghalichi, A. Juras, C. C. Bilgin, T. Günther, J. Storå, M. Jakobsson, M. de Kleijn, G. Mustafaoğlu, A. Fairbairn, J. Pearson, İ. Togan, N. Kayacan, A. Marciniak, C. S. Larsen, I. Hodder, Ç. Atakuman, M. Pilloud, E. Sürer, F. Gerritsen, R. Özbal, D. Baird, Y. S. Erdal, G. Duru, M. Özbaşaran, S. D. Haddow, C. J. Knüsel, A. Götherström, F. Özer, M. Somel, Variable kinship patterns in Neolithic Anatolia revealed by ancient genomes. *Curr. Biol.* **31**, 2455–2468.e18 (2021).

97. N. Patterson, A. L. Price, D. Reich, Population structure and eigenanalysis. *PLOS Genet.* **2**, e190 (2006).

98. S. Purcell, B. Neale, K. Todd-Brown, L. Thomas, M. A. R. Ferreira, D. Bender, J. Maller, P. Sklar, P. I. W. de Bakker, M. J. Daly, P. C. Sham, PLINK: A tool set for whole-genome association and population-based linkage analyses. *Am. J. Hum. Genet.* **81**, 559–575 (2007).

99. A. A. Behr, K. Z. Liu, G. Liu-Fang, P. Nakka, S. Ramachandran, Pong: Fast analysis and visualization of latent clusters in population genetic data. *Bioinformatics* **32**, 2817–2823 (2016).

100. É. Harney, N. Patterson, D. Reich, J. Wakeley, Assessing the performance of qpAdm: A statistical tool for studying population admixture. *Genetics* **217**, iyaa045 (2021).

101. I. Lazaridis, D. Nadel, G. Rollefson, D. C. Merrett, N. Rohland, S. Mallick, D. Fernandes, M. Novak, B. Gamarra, K. Sirak, S. Connell, K. Stewardson, E. Harney, Q. Fu, G. Gonzalez-Fortes, E. R. Jones, S. A. Roodenberg, G. Lengyel, F. Bocquentin, B. Gasparian, J. M. Monge, M. Gregg, V. Eshed, A.-S. Mizrahi, C. Meiklejohn, F. Gerritsen, L. Bejenaru, M. Blüher, A. Campbell, G. Cavalleri, D. Comas, P. Froguel, E. Gilbert, S. M. Kerr, P. Kovacs, J. Krause, D. McGettigan, M. Merrigan, D. A. Merriwether, S. O'Reilly, M. B. Richards, O. Semino, M. Shamoon-Pour, G. Stefanescu, M. Stumvoll, A. Tönjes, A. Torroni, J. F. Wilson, L. Yengo, N. A. Hovhannisyan, N. Patterson, R. Pinhasi, D. Reich, Genomic insights into the origin of farming in the ancient Near East. *Nature* **536**, 419–424 (2016).

102. N. Patterson, M. Isakov, T. Booth, L. Büster, C.-E. Fischer, I. Olalde, H. Ringbauer, A. Akbari, O. Cheronet, M. Bleasdale, N. Adamski, E. Altena, R. Bernardos, S. Brace, N. Broomandkhoshbacht, K. Callan, F. Candilio, B. Culleton, E. Curtis, L. Demetz, K. S. D. Carlson, C. J. Edwards, D. M. Fernandes, M. G. B. Foody, S. Freilich, H. Goodchild, A. Kearns, A. M. Lawson, I. Lazaridis, M. Mah, S. Mallick, K. Mandl, A. Micco, M. Michel, G. B. Morante, J. Oppenheimer, K. T. Özdoğan, L. Qiu, C. Schattke, K. Stewardson, J. N. Workman, F. Zalzala, Z. Zhang, B. Agustí, T. Allen, K. Almássy, L. Amkreutz, A. Ash, C. Baillif-Ducros, A. Barclay, L. Bartosiewicz, K. Baxter, Z. Bernert, J. Blažek, M. Bodružić, P. Boissinot, C. Bonsall, P. Bradley, M. Brittain, A. Brookes, F. Brown, L. Brown, R. Brunning, C. Budd, J. Burmaz, S. Canet, S. Carnicero-Cáceres, M. Čaušević-Bully, A. Chamberlain, S. Chauvin, S. Clough, N. Čondić, A. Coppa, O. Craig, M. Črešnar, V. Cummings, S. Czifra, A. Danielisová, R. Daniels, A. Davies, P. de Jersey, J. Deacon, C. Deminger, P. W. Ditchfield, M. Dizdar,

M. Dobeš, M. Dobisíková, L. Domboróczki, G. Drinkall, A. Đukić, M. Ernée, C. Evans, J. Evans, M. Fernández-Götz, S. Filipović, A. Fitzpatrick, H. Fokkens, C. Fowler, A. Fox, Z. Gallina, M. Gamble, M. R. González Morales, B. González-Rabanal, A. Green, K. Gyenesei, D. Habermehl, T. Hajdu, D. Hamilton, J. Harris, C. Hayden, J. Hendriks, B. Hernu, G. Hey, M. Horňák, G. Ilon, E. Istvánovits, A. M. Jones, M. B. Kavur, K. Kazek, R. A. Kenyon, A. Khreisheh, V. Kiss, J. Kleijne, M. Knight, L. M. Kootker, P. F. Kovács, A. Kozubová, G. Kulcsár, V. Kulcsár, C. Le Pennec, M. Legge, M. Leivers, L. Loe, O. López-Costas, T. Lord, D. Los, J. Lyall, A. B. Marín-Arroyo, P. Mason, D. Matošević, A. Maxted, L. McIntyre, J. McKinley, K. McSweeney, B. Meijlink, B. G. Mende, M. Menđušić, M. Metlička, S. Meyer, K. Mihovilić, L. Milasinovic, S. Minnitt, J. Moore, G. Morley, G. Mullan, M. Musilová, B. Neil, R. Nicholls, M. Novak, M. Pala, M. Papworth, C. Paresys, R. Patten, D. Perkić, K. Pesti, A. Petit, K. Petriščáková, C. Pichon, C. Pickard, Z. Pilling, T. D. Price, S. Radović, R. Redfern, B. Resutík, D. T. Rhodes, M. B. Richards, A. Roberts, J. Roefstra, P. Sankot, A. Šefčáková, A. Sheridan, S. Skae, M. Šmolíková, K. Somogyi, Á. Somogyvári, M. Stephens, G. Szabó, A. Szécsényi-Nagy, T. Szeniczey, J. Tabor, K. Tankó, C. T. Maria, R. Terry, B. Teržan, M. Teschler-Nicola, J. F. Torres-Martínez, J. Trapp, R. Turle, F. Ujvári, M. van der Heiden, P. Veleminsky, B. Veselka, Z. Vytlačil, C. Waddington, P. Ware, P. Wilkinson, L. Wilson, R. Wiseman, E. Young, J. Zaninović, A. Žitňan, C. Lalueza-Fox, P. de Knijff, I. Barnes, P. Halkon, M. G. Thomas, D. J. Kennett, B. Cunliffe, M. Lillie, N. Rohland, R. Pinhasi, I. Armit, D. Reich, Large-scale migration into Britain during the Middle to Late Bronze Age. *Nature* **601**, 588–594 (2022).

103. J. M. Monsalvo Antón, Atlas Histórico de la España Medieval (Síntesis, 2010).

104. S. Brunel, E. A. Bennett, L. Cardin, D. Garraud, H. Barrand Emam, A. Beylier, B. Boulestin,F. Chenal, E. Ciesielski, F. Convertini, B. Dedet, S. Desbrosse-Degobertiere, S. Desenne,J. Dubouloz, H. Duday, G. Escalon, V. Fabre, E. Gailledrat, M. Gandelin, Y. Gleize, S. Goepfert, J. Guilaine, L. Hachem, M. Ilett, F. Lambach, F. Maziere, B. Perrin, S. Plouin, E. Pinard, I. Praud, I. Richard, V. Riquier, R. Roure, B. Sendra, C. Thevenet, S. Thiol, E. Vauquelin, L. Vergnaud, T. Grange, E.-M. Geigl, M. Pruvost, Ancient genomes from present-day France unveil 7,000 years of its demographic history. *Proc. Natl. Acad. Sci.* **117**, 12791–12798 (2020).
